# Enabling the examination of long-term mortality trends by educational level for England and Wales in a time-consistent and internationally comparable manner

**DOI:** 10.1186/s12963-024-00324-2

**Published:** 2024-03-09

**Authors:** Fanny Janssen, Wanda Van Hemelrijck, Eva Kagenaar, Alison Sizer

**Affiliations:** 1 Netherlands Interdisciplinary Demographic Institute, KNAW/University of Groningen, Lange Houtstraat 19, 2511 CV The Hague, The Netherlands; 2 Population Research Centre, Faculty of Spatial Sciences, University of Groningen, Groningen, The Netherlands; 3 Centre for Longitudinal Study Information & User Support (CeLSIUS), Department of Information Studies, University College London (UCL), London, UK

**Keywords:** Educational attainment, Educational classification, Mortality, Socioeconomic inequalities, Trends, Data issues

## Abstract

**Background:**

Studying long-term trends in educational inequalities in health is important for monitoring and policy evaluation. Data issues regarding the allocation of people to educational groups hamper the study and international comparison of educational inequalities in mortality. For the UK, this has been acknowledged, but no satisfactory solution has been proposed.

**Objective:**

To enable the examination of long-term mortality trends by educational level for England and Wales (E&W) in a time-consistent and internationally comparable manner, we propose and implement an approach to deal with the data issues regarding mortality data by educational level.

**Methods:**

We employed 10-year follow-ups of individuals aged 20+ from the Office for National Statistics Longitudinal Study (ONS-LS), which include education information from each decennial census (1971–2011) linked to individual death records, for a 1% representative sample of the E&W population. We assigned the individual cohort data to single ages and calendar years, and subsequently obtained aggregate all-cause mortality data by education, sex, age (30+), and year (1972–2017). Our data adjustment approach optimised the available education information at the individual level, and adjusts—at the aggregate level—for trend discontinuities related to the identified data issues, and for differences with country-level mortality data for the total population.

**Results:**

The approach resulted in (1) a time-consistent and internationally comparable categorisation of educational attainment into the low, middle, and high educated; (2) the adjustment of identified data-quality related discontinuities in the trends over time in the share of personyears and deaths by educational level, and in the crude and the age-standardised death rate by and across educational levels; (3) complete mortality data by education for ONS-LS members aged 30+ in 1972–2017 which aligns with country-level mortality data for the total population; and (4) the estimation of inequality measures using established methods. For those aged 30+ , both absolute and relative educational inequalities in mortality first increased and subsequently decreased.

**Conclusion:**

We obtained additional insights into long-term trends in educational inequalities in mortality in E&W, and illustrated the potential effects of different data issues. We recommend the use of (part of) the proposed approach in other contexts.

**Supplementary Information:**

The online version contains supplementary material available at 10.1186/s12963-024-00324-2.

## Background

Monitoring long-term trends in socio-economic inequalities in health outcomes is of high societal and academic relevance [1]. Knowing whether mortality inequalities by socio-economic background are becoming smaller or larger is essential for understanding the health and social performance of a country [1], for informing political debates on social inequity and the sustainability of health care systems [2, 3], and for achieving equity in pension systems [4].

However, studying long-term trends in socio-economic inequalities in mortality is not straightforward, as it comes with large data requirements and many potential data quality issues. Data in which information on the socio-economic position of individuals is longitudinally linked to individual mortality records are preferred over cross-sectionally linked aggregate mortality and socio-economic data [5]. Even in the few countries that do have longitudinally linked data, these data are not always available for the whole national population [5]. Previous studies of long-term trends in socio-economic inequalities in mortality have mostly focused on mortality inequalities by educational attainment group [6–11]. This is because education is regarded as a more stable measure of socio-economic position than occupation and income, and is less prone to reverse causation at older ages [12]. Furthermore, long-term mortality data on individual income are generally lacking, and information on occupational class is considered to be less reliable for women [7]. Educational attainment is associated with different resources like knowledge, health awareness, and health literacy that prevent exposure to health risks (e.g. through employment type and/or healthy lifestyles) or are important for navigating the health care system, both of which minimize the risk of amenable death [13]. However, even when individually linked mortality data by educational level are available for a long time period, data issues can arise, including issues related to inconsistencies over time in how education information is collected and categorised.

In the UK, it has been acknowledged that the decennial census data used to allocate people to educational groups are not well-suited for the monitoring of long-term trends in socio-economic mortality inequalities, or for international comparisons of these trends [14–16]. Aside from issues related to the consistency of educational information over time (changes in the educational system,changes in the obtainment and valuation of qualifications), and issues related to the use of rather crude census data for educational information, three main issues relate specifically to the UK context (see [14]) and are potentially solvable. First, the educational information in the census is not available in a consistent manner over time, which hinders the study of time trends in educational inequalities in mortality. Second, the education information from the 1981 and 1991 Censuses cannot be classified according to the International Standard Classification of Education (ISCED), which impedes international comparisons. Third, the high proportion of respondents with missing educational level data makes it difficult to obtain reliable estimates of educational inequalities in mortality. These concerns regarding the data have contributed to doubts regarding the finding of earlier research of Mackenbach et al. 2016 [17] using these data that absolute educational inequalities in mortality have reduced from 1991 in England and Wales (E&W) and Scotland [15].

In addition to the aforementioned issues identified for the UK, there are two more general complications that are usually not discussed in research on educational inequalities in mortality. The first relates to the exclusion of emigrants to prevent bias when exact dates of emigration are missing for the whole time series, and the related identification of emigrants. When reporting of emigration is considered to be poor, individuals are ideally defined as emigrants if they were not present at the next census and did not die. If, for the last follow-up period, data for the next census is not yet available, this definition cannot be applied for the last follow-up period, which creates both inconsistencies in the time series, and potentially biased estimates for the last follow-up period. The second complication is that the sample-based mortality data used for the study of educational differences might not fully align with country–level mortality data for the total population, and that this alignment might change over time, thereby resulting in issues regarding the national representativeness of the outcomes.

No satisfactory solutions have been proposed to address these data issues, resulting in possibly biased estimates of educational inequalities in mortality, their trends over time, and differences therein between countries.

First, previous studies on long-term trends in educational inequalities in mortality for E&W ignored the 1981–2000 period altogether (e.g., [7, 18]), and could therefore only study the overall trend between 1971–1980 and 2001–2010, and not potential trend breaks therein.

Second, most previous comparative studies on trends in educational inequalities in mortality for different European countries that covered the 1990s and 2000s distinguished only two educational groups for E&W (low + middle versus high), while distinguishing three groups (low, middle, high) for other countries [17, 19]. Since in Europe, the mortality levels and trends of the middle educated generally differ from those of the low educated [7], this could lead to biased inequality levels and trends for E&W, and, consequently biased cross-national differences. Furthermore, not being able to separately study the low educated prevents the calculation of inequality measures in an internationally comparable manner. Moreover, comparing two educational groups compared to three may obscure inequalities.

Third, in previous studies, individuals with missing educational level were generally omitted, which could result in less robust outcomes when the number of individuals omitted is large. Fourth, possibly owing to large shares of missing information on education among the elderly, previous studies have generally used a maximum age of 69 or 74 (e.g., [7, 8, 14]). This results in limited information on socio-economic inequalities in mortality for the elderly, despite their importance [20], and in the available outcomes on socio-economic inequalities not being applicable for a large portion of the population with a high concentration of deaths [3]. We propose and implement an approach to address the data issues regarding mortality data by educational level for E&W, to enable the examination of long-term mortality trends by educational level for E&W in a time-consistent and internationally comparable manner. In doing so, we illustrate the potential effects of the different data issues involved, and show the resulting long-term trends in educational inequalities in mortality in E&W by means of internationally established inequality measures.

While our analysis, the implementation of our adjustment approach, and our results are specific to the study of trends in mortality by educational level for E&W, our study has added value beyond this particular context. First, increased knowledge of data issues and their potential effects could contribute to increased awareness and transparency regarding data issues in the field of educational inequalities in mortality and health. Second, the resulting internationally comparable long-term mortality trends by educational level for E&W will not only aid the monitoring of trends in educational inequalities in health outcomes for E&W but also for the wider European context. Third, the proposed adjustment methodology could be (partially) adopted to identify and/or deal with data-driven inconsistencies in educational health/mortality inequalities in other countries, and/or in other time series beyond the field of educational inequalities in mortality/health.

## Data and methods

We used information from the Office for National Statistics Longitudinal Study (ONS-LS) [21], which includes individual information on highest educational attainment obtained at the time of the different decennial censuses (1971, 1981, 1991, 2001, 2011), linked with individual death records, for an approximately 1% representative sample of the population of E&W [22]. We employed 10-year follow-ups of the ONS-LS sample members aged 20 years and older at the different censuses. We followed individuals until the next census, or until the date when vital status information was last linked to the ONS-LS (currently 31 December 2017). See Additional file 1: Section 1 for more detailed information regarding the data and data source.

In line with previous international comparative research (e.g., [7]), we wanted to distinguish between three educational attainment groups using the ISCED classification [23]: low (no, pre-primary, primary, and lower secondary education,ISCED-1997 0–2), middle (upper secondary and post-secondary non-tertiary education; ISCED-1997 3–4), and high (tertiary education; ISCED-1997 5–6).

Once we defined the educational variable in line with this classification—see the section “Optimising census information on educational attainment at the individual level” below, we rearranged the individual-level cohort data (20+) for the five different follow-up periods (1971–1981, 1981–1991, 1991–2001, 2001–2011, 2011–2017) into aggregate period data (30+ ; 1972–2017). First, we assigned the individual cohort data to single ages and single calendar years using person days at risk. Second, we aggregated the data to obtain all-cause mortality data by educational level, sex, and single year of age (30+) for single calendar years from 1972 up to 2017.

Four main data issues impeded the construction of consistent time trends in mortality by educational level (low, middle, high) for E&W (30+) across 1972–2017. We list them below. See Additional file 1: Section 2 for more detailed information.

1. **Across the censuses, the questions and variables regarding education vary, making the information on educational attainment inconsistent over time.** In the 1981 and 1991 censuses, respondents were asked to disregard any qualifications normally obtained at school. Consequently, individuals who obtained an upper secondary-level educational qualification (the cut-off between low and middle educated), but no other post-18 qualification, were misclassified as low educated. In addition, the main educational variable in the 1991 Census does not distinguish a separate category for no qualifications (i.e., low educated), but instead uses only one code for “not applicable or missing” combined. Furthermore, individuals with professional qualifications (i.e., teaching, nursing, midwifery, health visitors) but not a degree are assigned to the sub-degree level (i.e., middle educated) in 1971, 1981, and 1991, but to the degree level (i.e., high educated) in 2001 and 2011. Finally, in 2001 and 2011, a separate category for “other” exists that is not defined in the same way in both censuses.
2. **Information on educational attainment is not in all censuses available for all adults, resulting in important missing educational information.** Respondents aged 70 and older in the 1971 Census and respondents aged 75 and older in the 2001 Census were, respectively, not required to answer the question regarding their educational level or were not asked about their educational level. In addition, particularly in the 1971 Census, non-response resulted in missing educational information. See Additional file 1: Table S2 for the numbers of people with missing educational information at the time of the decennial censuses.
3. **Emigration could not be defined consistently over time, creating both inconsistencies in the time series, and potentially biased estimates for the last follow-up period. **In E&W, official emigration statistics with emigration dates are considered incomplete [24]. Therefore, in line with common practice, we defined emigrants as individuals who were absent at the next census and did not die. Since 2021 Census data are not yet available, we could, for the final follow-up period (2011–2017), only define, and subsequently exclude, emigrants based on the available data in the ONS-LS, which stem from the registered embarkations (emigrations) data from the NHS Central Register (NSHCR). However, it is estimated that only 50% of emigrations are reported to the NHSCR [24].
4. **The alignment of our ONS-LS data selection with country-level population and mortality data for the general E&W population is imperfect, and changes over time, resulting in issues regarding the national representativeness of our outcomes.** First, the increases in the sample size of the ONS-LS (see Additional file 1: Table S4) have not been in line with increases in the size of the national population of E&W (Fig. 2a). Second, our modifications to the data to avoid bias (e.g. the exclusion of emigrants (see previous point), and not counting immigrants until the census after they arrived in E&W to ensure complete information on educational attainment), resulted in additional differences with country-level data (Fig. 2a, b).

In order to address these data issues, we implemented a dual data adjustment approach with adjustments at the individual and aggregate level.

At the individual level, we optimised the available education information. That is, to generate a consistent educational classification over time that distinguishes the low, middle and high educated, and aligns well with the ISCED classification, we used information on the educational system in E&W compared to the ISCED classification (e.g. [25]), the original census data on education [26], and additional information on the educational variables in the ONS-LS [27]. Compared to previous research that merely used the summary educational variables in the ONS-LS, we used—as well—the more detailed underlying educational variables in the ONS-LS that more directly align with the census questions. Furthermore, we used the general strategy of using educational information from other censuses (e.g. [28]), to deal with missing educational information at the individual level.

At the aggregate level, we identified and adjusted for trend discontinuities related to the identified data issues, and for undesirable differences with country-level mortality data for the total population. The adjustment involves the reallocation of people (personyears) in line with information on the data issue at stake, and the consequent reallocation of deaths, by applying the relevant mortality rates. The adjustment, furthermore, includes the use of existing approaches to deal with data-quality related trend discontinuities in cause-specific mortality [29, 30]. In adjusting our data with administrative country-level mortality data, we maintained the differences between educational groups in the partly-adjusted ONS-LS data. In the absence of quantitative data that can—without problems—be used as external validation beyond the administrative country-level mortality data (see Additional file 1: Section 3.1), we regard the careful study of trends over time in different outcome measures as an important means of internal validation. Trend discontinuities that can be clearly linked to the identified data issues (from here onwards: “unrealistic trend discontinuities”) are—in our view—a sign of invalid data, and therefore should be adjusted. Hence, our choice for the dual data adjustment approach. The different elements of our approach are described in more detail in the section ‘Elements of the adjustment approach’ below.

We show the effects of our adjustments at the individual level on the frequencies of ONS-LS members by educational level at the different censuses. At the aggregate level, we studied discontinuities in and assessed the effects of our adjustments on trends over time in the share of personyears by educational level, the share of deaths by educational level, and the total and education-specific trends over time in the crude death rate (CDR) and the age-standardised death rate (SDR), for those aged 30 and over by sex from 1972 up until 2017. To estimate the SDRs, we applied the 2013 revision of the European Standard Population [31] to the age-specific mortality rates by year, sex and education group.

The overall outcome of our adjustments is the obtainment of time-consistent aggregate mortality data based on the internationally comparable categorisation of educational attainment into the low, middle, and high educated. Based on these adjusted data, we were able to study long-term trends in educational inequalities in mortality in E&W using internationally established measures. We show the trends over time in both absolute and relative educational inequalities in mortality by means of the slope index of inequality (SII) and the relative index of inequality (RII), respectively. Since these inequality measures account for the educational distribution of the population, they are considered well-suited for the comparison of inequalities over time and between countries [7, 32]. In calculating the RII, we applied Poisson regression as per Moreno-Betancur et al. [33] to the adjusted data by educational level (low, middle, high). The SII was calculated from the RII and the SDR in the general population [7]. We applied segmented regression to identify potential changes in the trends, using the R package *Segmented*.

## Elements of the adjustment approach

Below the different elements of our data adjustment approach are listed, starting with the adjustment made at the individual level, and subsequently the adjustments made at the aggregate level. See for more detailed information Additional file 1: Section 4 (individual-level adjustments) and Section 5 (aggegrate-level adjustments).

### Optimising census information on educational attainment at the individual level

To arrive at a consistent classification of educational level over time that distinguishes the low, middle and high educated, and aligns well with the ISCED-1997, we optimised the individual information regarding educational attainment that was available by using, where appropriate, the more detailed underlying educational variables in the ONS-LS in addition to the main ONS-LS educational variables. See Appendix for our final educational classification. First, using the more detailed education information in the 1991 Census, we were able to distinguish those ONS-LS members in the “missing or not applicable” category of the main educational variable who really did not have educational qualifications (i.e., were low educated) from those ONS-LS members who simply had missing education information due to, for example, non-response. Second, using the more detailed information in the 2001 and 2011 censuses, we were able to identify ONS-LS members with professional qualifications (i.e., teaching, nursing, midwifery, health visitors) but no degree, and classify them as middle educated, in line with the classification used in the 1971, 1981, and 1991 censuses. Third, we changed the classification of “other” in 2001 so that it better matched the “other” category in 2011, and categorised them as middle educated in line with the listed underlying qualifications (e.g. City and Guilds).

We dealt with the missing educational information as much as possible at the individual level. We used the general strategy of using educational information from other censuses (e.g. [28]). In doing so, we regarded the information from the previous census as the most reliable, and only used the education information from the 1991 Census for individuals aged 75 and over in the 2001 Census as they constituted a distinct group with no educational information at all based on the 2001 Census owing to the way that the question was asked in census form.

We rearranged the adjusted individual data (for ONS-LS members aged 20+ at the different censuses) into aggregate period data (for those aged 30+ ; 1972–2017) and refer to these aggregate data as our baseline data. Below, the adjustments to the aggregate data are listed.

### Proportional redistribution of missings at the aggregate level (“redistribution missings”)

Instances of missing educational information (i.e., the remaining missings among those aged 75 and older in 2001, missings among those aged 70 and older in 1971, and missings for the non-elderly) that could not adequately be addressed at the individual level were addressed at the aggregate level. That is, we proportionally redistributed the deaths and personyears with missing educational information to the low, middle, and high education categories according to the smoothed relative share of personyears and deaths of the respective educational category by year, sex, and age. The underlying assumption that those with missing education were missing at random is made implicitly in previous research that omitted those with missing educational information. We choose to directly implement this assumption to optimise our sample size, and to obtain complete period data for those aged 30 and over from 1972 onwards.

### Careful study of trends over time and comparison with national administrative data

An important element of the remaining adjustments at the aggregate level was the careful study of trends over time in the share of personyears and deaths by educational level (%), and in the crude death rate (CDR), and the age-standardised death rate (SDR) by and across educational levels for individuals aged 30+ , 30–74, and 75+ . Exploiting the yearly character of the data and the available information on the data issues, we identified discontinuities in these trends that can be linked to the identified data issues (“unrealistic trend discontinuities”), and therefore should be adjusted for.

In addition, we compared the levels and trends in the SDR, the CDR, the deaths and population numbers for the three educational groups combined based on our ONS-LS data selection, with those based on administrative country-level mortality and population data, for which we used data from the Human Mortality Database (HMD) [34]. We did so, to examine the extent of bias from the inevitably inconsistent definition of emigration (see before), and to obtain additional insights regarding the national representativeness of the overall mortality levels and trends based on the ONS-LS data, which we subsequently correct for.

### Adjustment of unrealistic trend discontinuities (“trend break 1981”)

**Fig. 1:**
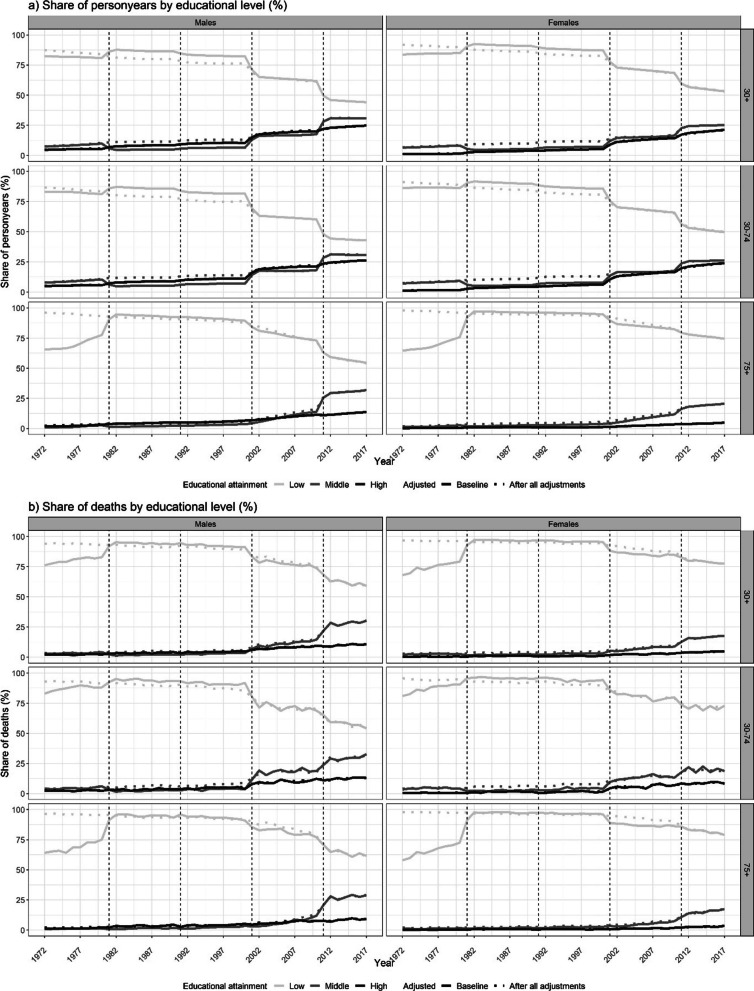
Trends in the share of personyears and deaths by educational attainment group (%) before and after adjustment of the unrealistic trend discontinuity in 1981, for the broad age groups (30+ , 30–74, 75+), by sex, England and Wales, 1972–2017

We adjusted for discontinuities in the trends due to inconsistent information on educational level over time by using existing approaches to deal with data quality-related trend breaks in cause-specific mortality [29, 30]. This was done by shifting (upwards or downwards) the levels before or after an unrealistic trend discontinuity to the levels following or preceding the discontinuity, respectively,thereby correcting for the underlying trends (see Additional file 1: Figure S18). Based on our careful study of trends over time we identified unrealistic discontinuities in the share of personyears and the share of deaths for the middle and the low educated in 1981, that lasted for the 1981–2001 period for those aged 30–74 and for the 1981–2011 period for those aged 75+ (see Results; see Fig. 1). These trend discontinuities could be attributed to the misclassification of some of the middle-educated individuals to the low educated category in the 1981 and 1991 censuses. We used a two-step approach to adjust for this unrealistic trend discontinuity in 1981. First, we adjusted, by sex, for the trend discontinuity in the age-specific shares of personyears by educational level for the middle educated in 1981 to obtain a yearly estimate of the age-specific personyears to be redistributed from the low to the middle educated. We selected the middle-educated for this purpose, because it is less likely that, for them, the educational expansion in E&W [35, 36] resulted in a sudden increase in the share of personyears in 1981. Second, we calculated the age-specific deaths to be redistributed from the low to the middle educated by year and sex by multiplying the redistributed age-specific personyears with the smoothed age-specific mortality rates for the middle educated.

### Correcting for the inevitably inconsistent definition of emigration over time (“emigration”)

To correct for the invevitably inconsistent definition of emigration, we estimated the number of “missed” emigrants (thus, overestimated personyears) in 2011–2017, by sex. We did so by subtracting the difference in the sample size between the 2011 and the 2001 ONS-LS follow-up periods (representing part of the discontinuity in 2011 not related to the inconsistent emigration definition) from the observed difference in personyears between 2012 and 2010 (representing the total trend discontinuity in 2011). We then estimated the sex-, age-, and education-specific overestimated personyears by applying the age- and education-specific distribution of reported emigrations in 2011–2017 to the “missed” emigrants. In doing so, we corrected for educational differences in the reporting of embarkations based on a comparison of the educational distribution of emigrants in 2001–2011 for the two emigration definitions. Next, we calculated by how much the deaths were overestimated by applying the observed strata-specific death rates to the age-, sex-, year-, and education-specific overestimated personyears.

### Alignment of our ONS-LS data selection with country-level administrative data (“alignment national data”)

We adjusted for the imperfect and time-varying alignment of our ONS-LS data selection for the three educational groups combined with administrative country-level population and mortality data [34] by matching the age-, sex-, and year-specific deaths and personyears for the three educational groups combined in the ONS-LS with the respective administrative numbers from the HMD divided by 100, in line with the ONS-LS being a 1% sample of the population of E&W. As such we obtained similar age-, sex- and year-specific death rates for our ONS-LS data selection as compared to the country-level administrative data. To maintain the education-specific differences observed in our adjusted ONS-LS data selection, we applied the existing age-, sex-, and year-specific shares of the deaths and personyears by educational level to the adjusted total deaths and personyears.

## Results

The optimisation of the individual data regarding education level, and the resulting classification of highest educational attainment into the low, middle and high educated (see Appendix) changed the educational distribution of ONS-LS members aged 20+ at the different censuses compared to the use of the main ONS-LS education variables (Additional file 1: Table S6). First, the 352,355 ONS-LS members with “missing or not applicable” values in the summary education variable in 1991, were redistributed into 350,658 without qualifications (i.e., low educated), and 1,697 with truly missing educational information (i.e., missing). Second, respondents in 2001 and 2011 with professional qualifications but without a degree (11,125 in 2001 aged 20–74 (74,826 minus 63,701); 31,524 in 2011 aged 20+ (128,648 minus 97,124)) were classified as middle educated (“Level 3”) instead of high educated (Level 4 and higher). Third, 5,900 ONS-LS members with “other professional qualifications” in 2001 (27,256 minus 21,356) were moved from the “other” to the middle-educated category, so that the “other” category in 2001 more closely resembled that in 2011. Finally, we treated the “other” educated (21,356 in 2001; 29,002 in 2011) as middle educated.

In addition, of the 41,960 ONS-LS members aged 75 and older in 2001, who all had missing educational information, 91.6% were assigned to either the low (35,564), middle (1,557), or high (1,297) educated category based on their education information in 1991 (Additional file 1: Table S7).

Table 1 shows the frequencies of the final educational variable, by sex, after optimising the individual data regarding education level. The high share of ONS-LS members with missing educational information at the 1971 Census is predominantly due to missing information for those aged 70 and over, for whom education information from an earlier census year is unavailable. The increase in the share of the low educated, and the concomitant decline in the share of the middle educated from 1971 to 1981 is not in line with the educational expansion in E&W [35, 36]. This issue is dealt with in our adjustment at the aggregate level. The levels and trends in the gender difference in the proportion of high educated are in line with the increased participation of women in higher education in E&W [35, 37].

**Table 1:** Educational distribution for ONS-LS members aged 20+ at the 1971–2011 censuses, according to our final educational variable, before applying adjustments at the aggregate level*, by sex

Census	Low (%)	Middle (%)	High (%)	Missing (%)	Total (N)
Males					
1971	80.01	9.34	5.08	5.57	175,172
1981	87.76	4.41	7.81	0.02	182,585
1991	84.02	5.84	9.66	0.49	193,109
2001	63.13	17.17	18.70	0.99	194,105
2011	45.10	31.35	23.23	0.32	215,763
Females					
1971	82.59	8.03	1.35	8.03	193,762
1981	91.64	4.82	3.53	0.00	200,524
1991	88.39	6.40	4.86	0.35	213,163
2001	69.48	15.82	13.40	1.30	214,110
2011	53.88	25.84	20.04	0.24	234,486

Source data: ONS-LS

*The proportional redistribution of missings at the aggregate level does not substantially alter the observed trends over time in the proportions, nor the observed sex differences and trends therein

After rearranging the final individual-level cohort data to aggregate period data, the people with missing educational level contributed—overall—183,771 personyears (1.3% of the total personyears) and 12,472 deaths (4.8% of the total deaths) (Additional file 1: Table S11). The proportional redistribution of missings across the different educational groups, resulted in assigning 167,712 personyears (91.3%) to the low educated, 10,191 personyears (5.5%) to the middle educated, and 5,868 personyears (3.2%) to the high educated category (Table 2; Additional file 1: Table S11). Of the deaths with missing educational level information, 11,948 (95.8%) were assigned to the low educated, 333 (2.7%) to the middle educated, and 191 (1.5%) to the high educated category (Additional file 1: Table S11). The proportional redistribution of missings primarily resulted in higher CDR levels for the low educated in 1972–1981 and 2001–2011 (Additional file 1: Figure S13). This was because in those years it were mainly the elderly (with high mortality rates) who had missing educational information, and most of them were assigned to the low educated group.

**Table 2:** Effect—compared to the baseline—of the different adjustments at the aggregate level on the number of personyears (PY) and deaths, and the final fully adjusted numbers, for those aged 30 and older, by educational attainment group and period, England and Wales, 1972–2017

	Educational attainment							
Period and adjustment	Low educated		Middle educated		High educated		Total*	
	PY	Deaths	PY	Deaths	PY	Deaths	PY	Deaths
**1972–1981**								
Baseline numbers	2,401,860	46,773	221,422	1,676	93,808	787	2,868,427	59,305
Redistribution missings	140,400	9,730	7,652	224	3,286	114	151,338	10,069
Trend break 1981	− 11,829	− 91	11,829	91	0	0	0	0
Emigration	0	0	0	0	0	0	0	0
Alignment national data	− 93,183	− 2,559	− 6,061	− 88	− 2,573	− 42	− 101,818	− 2,688
Fully adjusted numbers	2,437,247	53,854	234,842	1,903	94,520	860	2,766,608	56,617
**1982–1991**								
Baseline numbers	2,638,435	55,343	149,255	1,179	171,166	1,274	2,959,721	57,824
Redistribution missings	759	27	47	0	59	0	865	27
Trend break 1981	− 161,129	− 1,227	161,129	1,227	0	0	0	0
Emigration	0	0	0	0	0	0	0	0
Alignment national data	− 59,204	− 1,726	− 3,452	− 67	− 1,394	− 34	− 64,050	− 1,827
Fully adjusted numbers	2,418,861	52,417	306,978	2,339	169,832	1,240	2,895,671	55,997
**1992–2001**								
Baseline numbers	2,610,425	53,205	225,213	1,847	243,174	1,560	3,090,970	56,946
Redistribution missings	10,380	312	799	12	979	10	12,157	334
Trend break 1981	− 155,787	− 1,001	155,787	1,001	0	0	0	0
Emigration	0	0	0	0	0	0	0	0
Alignment national data	19,059	− 2,382	11,816	− 102	9,680	− 58	40,555	− 2,542
Fully adjusted numbers	2,484,077	50,134	393,615	2,757	253,834	1,512	3,131,525	54,404
**2002–2011**								
Baseline numbers	2,107,572	42,210	547,093	5,146	520,908	2,709	3,194,054	52,093
Redistribution missings	15,767	1,869	1,436	94	1,277	65	18,480	2,028
Trend break 1981	− 6,762	− 320	6,762	320	0	0	0	0
Emigration	− 8,610	− 65	− 5,626	− 20	− 6,678	− 12	− 20,915	− 97
Alignment national data	123,558	− 1,886	35,925	− 151	57,086	− 49	216,569	− 2,086
Fully adjusted numbers	2,231,525	41,808	585,590	5,388	572,593	2,714	3,389,708	49,910
**2012–2017**								
Baseline numbers	1,136,186	22,057	627,001	6,956	495,251	2,219	2,259,368	31,245
Redistribution missings	407	10	257	0	267	0	931	10
Trend break 1981	0	0	0	0	0	0	0	0
Emigration	− 67,586	− 493	− 44,163	− 159	− 52,419	− 84	− 164,169	− 736
Alignment national data	27,081	− 105	23,035	30	29,722	63	79,837	− 12
Fully adjusted numbers	1,096,087	21,469	606,130	6,827	472,820	2,198	2,175,037	30,494
**1972–2017**								
Redistribution missings	167,712	11,948	10,191	330	5,868	189	18,3771	12,467
Trend break 1981	− 335,507	− 2,638	335,507	2,638	0	0	0	0
Emigration	− 76,197	− 558	− 49,789	− 179	− 59,097	− 95	− 185,083	− 833

Source data: ONS-LS & HMD. *Total is the total population including the missings for our baseline data and the sum of the unrounded numbers for low, middle and high educated in the other cases

The previous steps of our adjustment resulted in mortality data by educational level disaggregated into the low, middle and high educated, in line with international practice. In addition, we obtained mortality data for all those aged 30 and over—so including the elderly—for the whole time series from 1972 up to 2017. In the next step we checked for discontinuities in the trends over time that can be linked to the identified data issues, and we compared our data with national administrative data to examine the extent of bias from the inevitably inconsistent definition of emigration, and to obtain additional insights regarding the national representativeness of the overall mortality levels and trends based on the ONS-LS data.

The careful study of the trends over time in the partly-adjusted data pointed to an unrealistic upward shift in the share of personyears for the low educated in 1981 that resulted in elevated shares of personyears for the 1981–2001 period for those aged 30–74 and for the 1981–2011 period for those aged 75+ (Fig. 1a). There was also a concurrent unrealistic downward shift in the share of personyears for the middle educated in 1981 that resulted in decreased shares of personyears for the 1981–2001 and 1981–2011 periods, respectively. A similar pattern was observed for the share of deaths (Fig. 1b). In addition, the CDR levels for low educated men (not women) appeared to be lower in the 1981–2001 period than in the preceding and following periods (Additional file 1: Figure S16). These trend discontinuities are a likely result of the misclassification of some of the middle-educated individuals (with lower mortality) to the low educated category in the 1981 and 1991 censuses.

**Fig. 2:**
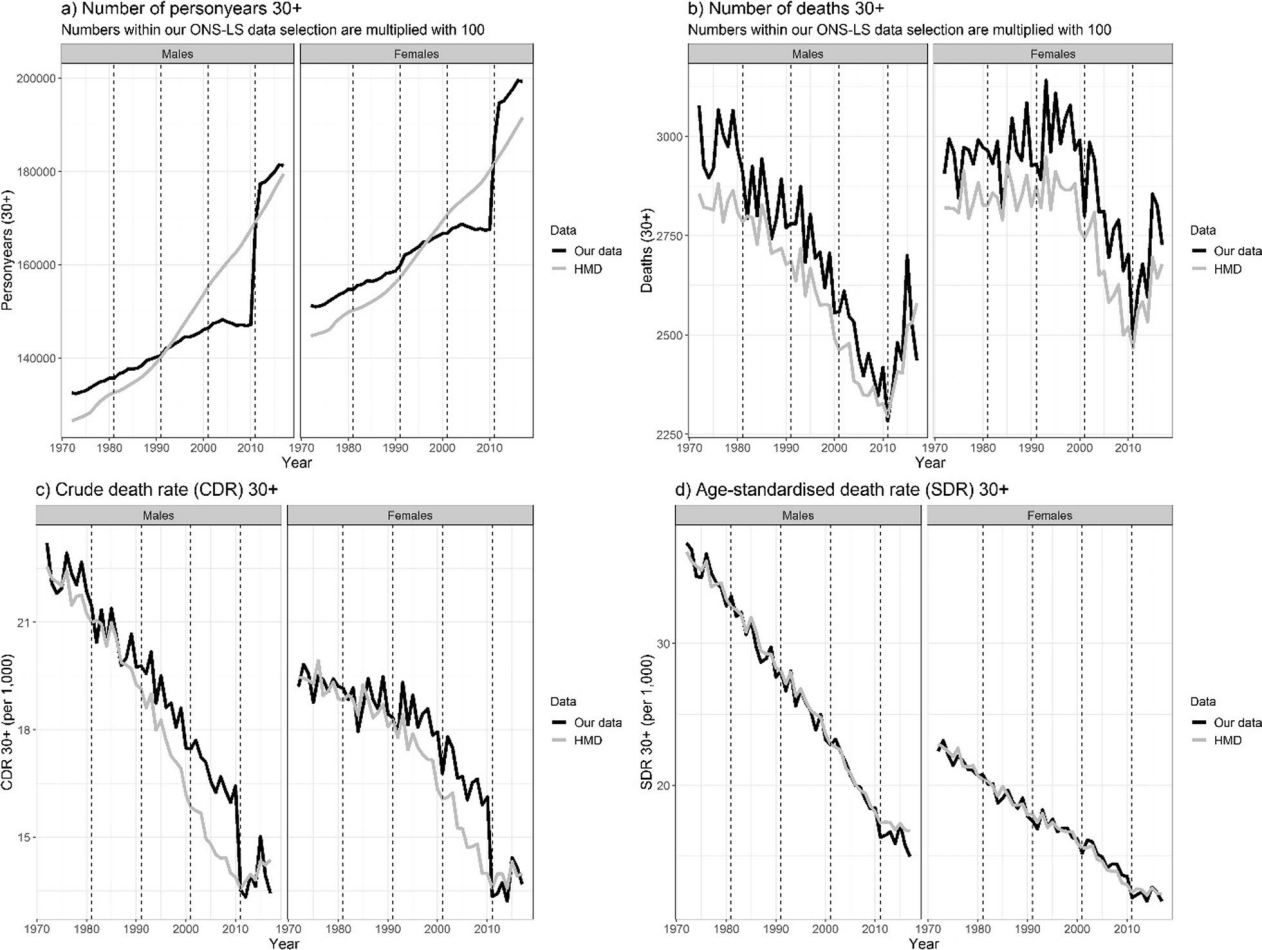
Trends in the number of personyears, the number of deaths, the crude death rate (CDR), and the age-standardised death rate (SDR), comparing our ONS-LS data selection with country-level administrative data [34], for those aged 30 and older, for the three educational groups combined, by sex, England and Wales, 1972–2017

Furthermore, for the three educational groups combined, we identified a sudden increase in the personyears between 2010 and 2012, resulting in an abrupt drop in CDR and SDR levels in 2011, which was not present in the administrative country-level data from HMD (Fig. 2). These trend discontinuities can partly be explained by the different definition of emigration from 2011 onwards, resulting in an underestimation of emigrations, and consequently an overestimation of personyears. In addition, the large increase in the sample size of the ONS-LS 2011 follow-up period compared to the ONS-LS 2001 follow-up period (Additional file 1: Table S4) contributed to the observed sudden increase in personyears.

Our comparison with administrative country-level mortality data from HMD (Fig. 2), furthermore illustrated some additional issues regarding the representativeness of our ONS-LS data selection. That is, the increase over time in the personyears based on our ONS-LS data selection differed from the national trend. Consequently, the CDR was substantially higher than that in the country-level data in 1990–2010. Moreover, the SDR among men was substantially higher than that of the country-level data from 2011 onwards.

In the subsequent steps of our adjustment we adjusted for the identified trend discontinuities and identified issues regarding the representativeness of our data.

The adjustment for the unrealistic trend discontinuities in 1981 in the share of personyears and the share of deaths for the low and middle educated resulted, at the aggregate level, for those aged ≥ 30, in the redistribution of 335,507 personyears and 2,638 deaths from the low educated to the middle educated category for the 1981–2011 period (Table 2; Additional file 1: Table S14). For 1982, the adjustment in personyears amounted to 6.4% for men and 4.7% for women (Additional file 1: Table S12). The adjustment addressed the unrealistic discontinuities in the age-specific share of personyears for both the middle and the low educated in 1981, and substantially reduced the unrealistic discontinuities in 2001 (for ages 30–74) and in 2011 (for ages 75+) (Fig. 1a; Additional file 1: Figure S19). Similarly, the trends in the age-specific shares of deaths by educational level no longer showed discontinuities in 1981 (Fig. 1b; Additional file 1: Figure S22), and the remaining discontinuities in 2001 (for ages 30–74) and in 2011 (for ages 75+) could be linked to the discontinuities in the share of personyears by educational level, and the educational expansion in E&W [35, 36]. For the low educated, the adjustment resulted in slightly higher CDR levels in the 1981–2001 period (Additional file 1: Figure S23),and for the middle educated, the underlying smoothing of the age-specific death rates resulted in slightly changed SDR and CDR levels (Additional file 1: Figure S23-S25).

The adjustment for the inevitably inconsistent definition of emigration for the periods before and after 2011 resulted, at the aggregate level, in a decrease of 185,085 personyears in total for the 2011–2017 period (95,913 for men; 89,170 for women) (Table 2; Additional file 1: Table S14). This resolved part of the unrealistic trend discontinuity in the sex-specific personyears in 2011 (Additional file 1: Figure S27). The remaining trend discontinuities in the number of personyears by educational level in 2011 were more in line with expectations given the impact of educational expansion in E&W [35, 36]. The effect on the death numbers was small (Table 2; Additional file 1: Figure S28), as expected. Consequently, the CDRs (both total and education-specific) increased slightly after 2011 (Additional file 1: Figure S29).

The alignment of the deaths and personyears with the country-level administrative data resulted, at the aggregate level, in slightly elevated deaths and personyears at younger ages from 1991 onwards (Additional file 1: Figures S32-S33), and—in general—in age-specific mortality rates that fluctuate less and are more in line with the exponential increase in mortality rates with age after age 30 [38] (Additional file 1: Figure S34). This alignment also resolved discontinuities in the CDR and the SDR between 2010 and 2012 for the total population (Fig. 2). Although differences between educational groups were maintained, the alignment resulted in lowered CDR levels for the low educated in the 1992–2011 period because of their high numbers (Additional file 1: Figure S35). While for women, the SDR by educational level was minimally affected, for men the SDR levels increased from 2011 onwards for all three educational groups (Additional file 1: Figure S36).

**Fig. 3:**
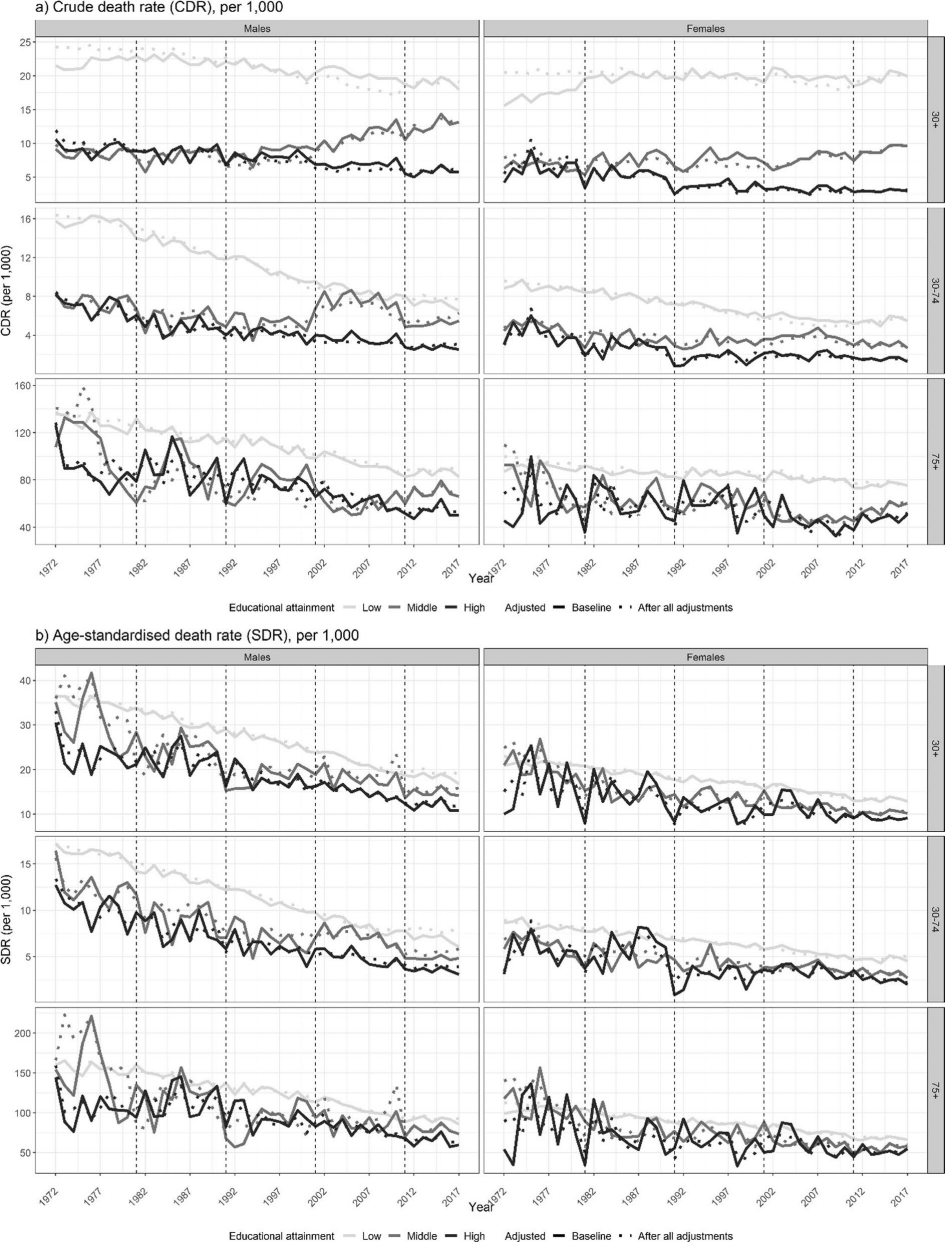
Trends in the crude death rate (CDR) and the age-standardised death rate (SDR) by educational attainment group before and after all of the adjustments at the aggregate level, for the broad age groups (30+ , 30–74, 75+), by sex, England and Wales, 1972–2017

In summary, the data adjustments at the aggregate level resolved the identified issues regarding the representativeness of our data and the identified unrealistic discontinuities in the trends over time for all the main outcome measures by and across the educational levels (Figs. 1, 2, 3). The higher CDR for the low educated in the 1972–1981 period resulted from the proportional redistribution of the “missings”, and the higher CDR for the low educated category in the 1982–2001 period resulted from the adjustment of the unrealistic trend discontinuity in 1981 (Fig. 3). The lower CDR levels for 2002–2011, particularly for the low educated category, resulted from the alignment of the data with the country-level administrative data. The impact of the adjustments on the SDR was small (Fig. 3) and was primarily the result of smoothing. However, the age-specific death rates by educational level changed substantially (Additional file 1: Figure S41).

**Fig. 4:**
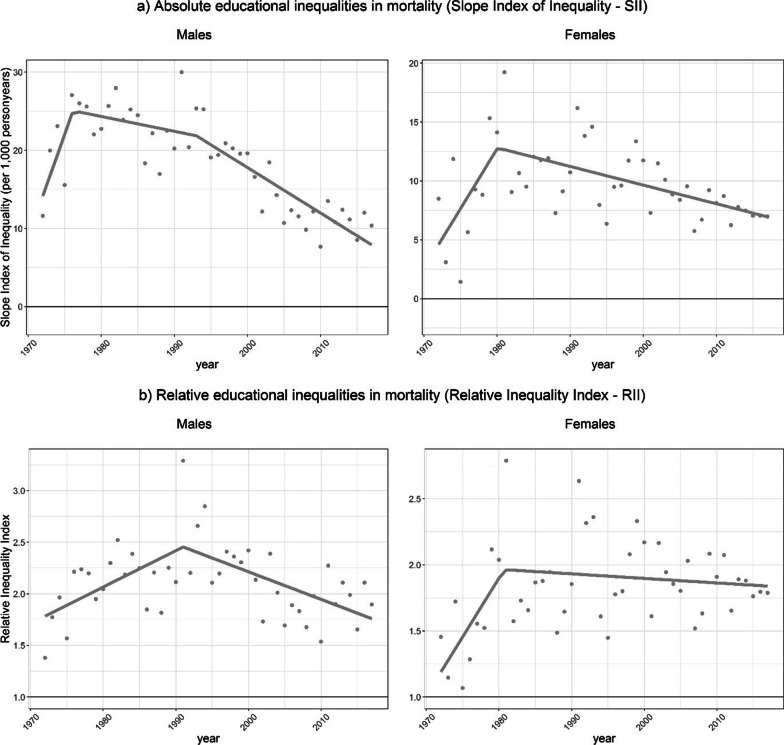
Trends in absolute and relative educational inequalities in mortality measured using data for the low-, middle- and high-educated, for those aged 30 and older, by sex, England and Wales, 1972–2017. Solid lines represent the outcomes of segmented regression

Based on the final adjusted data, involving both the adjustments at the individual level and the aggregate level, we were able to study trends over time (1972–2017) in educational inequalities in mortality for those aged 30 and older in E&W using internationally established methods. That is, we obtained trends over time in both the slope index of inequality (SII) and the relative index of inequality (RII) using data disaggregated into the low, middle and high-educated in line with common practice (e.g. [7, 8, 10]). The SII measures absolute inequalities and represents the rate difference of mortality between those with the very lowest and those with the very highest educational level, whereas the RII measures relative inequalities and represents the rate ratio of mortality among those with the very lowest educational level compared to those with the very highest educational level [7, 32]. We found that absolute educational inequalities in mortality—measured by the SII—first increased, but then decreased from 1977 onwards for men, and from 1980 onwards for women (Fig. 4). Relative educational inequalities in mortality—measured by the RII—also revealed an increase followed by a clear decrease from 1991 onwards for men and a slight decrease from 1981 onwards for women.

## Discussion

We proposed and implemented an approach to deal with the data issues regarding mortality data by educational level for E&W. The approach resulted in (i) a consistent and internationally comparable categorisation of educational attainment into the low, middle, and high educated; (ii) the adjustment for identified data-quality related discontinuities in the trends over time in the share of personyears and deaths by educational level, and in the CDR and the SDR by and across educational levels; (iii) complete mortality data by education for ONS-LS members aged 30+ in the 1972–2017 period, which aligns with country-level administrative mortality data for the total population; and (iv) the estimation of inequality measures using established methods. For those aged 30 and older, both absolute and relative educational inequalities in mortality first increased and subsequently decreased.

In adjusting the data, several assumptions were made to enable the examination of long-term trends in educational mortality inequalities in E&W in a time-consistent and internationally comparable manner for the first time (see [14]). In Additional file 1: Section 6.1 we have listed the different assumptions and provided our appraisal of them. The assumptions were made after careful consideration of alternatives. Nevertheless, caution is warranted particularly when analysing and interpreting the data regarding the elderly, specific to those aged 70 and over in 1972–1981, for which it was assumed that cases of missing education were distributed at random. Although this is a strong assumption, the same assumption is implicitly made in previous research that omitted those with missing educational information. Moreover, any other approach that deals with the absence of educational information for the elderly will be based on assumptions (e.g. [39]). Regarding the adjustment for the unrealistic trend discontinuity in 1981, the adjustment made is likely conservative. That is, we assumed that the educational expansion in E&W [35, 36] could not cause a sudden increase from 1980 to 1982 in the share of personyears for the middle educated. Moreover, after implementing this particular adjustment, we can still discern a small drop from 1980 to 1982 in the share of deaths for the middle educated for some age groups (Additional file 1: Figure S22).

It should also be noted that with the proposed approach we could not address all (potential) data issues relevant to the study of trends in educational inequalities in mortality. First, the consistency over time of the information provided in the education data is influenced by changes in the educational system (e.g. the changing structure and content of secondary schooling)[36] and changes in the obtainment (e.g. exam-based or not) and valuation of qualifications (e.g. a nursing qualification obtained in the 1970s is valued differently when compared to a nursing qualification obtained in the 2000s [40]. These data issues are inherent to the study of trends in educational mortality/health inequalities. Second, the use of decennial census data for educational information comes with some additional drawbacks aside from the addressed changes in coverage and wording of the census questions, in that the data is not provided annually, and the information provided tends to be crude [41]. On the other hand (i) changes in coverage and wording of questions are inherent to any long-term data collection, and the more detailed the questions the more likely their wording will change over time, (ii) the census covers the whole population including the institutionalised who are often omitted in surveys, and (iii) non-response is generally lower for a census compared to other data collection methods. Third, we were not able to avoid low numbers of people in certain strata, which can lead to fluctuations and uncertainty in inequality measures [14]. This mainly concerns the middle and high educated categories up to 2001 and mostly to high educated women in the 1970s and 1980s. Given these low numbers, we caution that visual inspection of the trends alone should not be relied upon. Rather, we recommend the use of more formal techniques (e.g., regression and/or time series modelling) to investigate the relevant trends.

The proposed and implemented adjustment facilitates the study of long-term trends in educational inequalities in mortality in E&W in a time-consistent and internationally comparable manner. First, the adjustment, which resulted in mortality data for three educational categories, allows for the study of long-term trends in educational inequalities in mortality in E&W using time-consistent and internationally comparable inequality measures. Whereas de Gelder et al. [7], for example, presented the SII and the RII for E&W only for the 1970–74, 1975–79, 2000–04, and 2005–09 periods, for which the available data was disaggregated by low, middle and high educated, we could obtain similar measures for all the years since the early 1970s using our adjusted data. Our findings revealed non-linear trends in both the SII and the RII, which, particularly for the RII, shed a different light on past trends in educational inequalities in mortality. Second, the adjustment resulted in complete mortality data by education for ONS-LS members aged 30+ in the 1972–2017 period. As such the adjustment allows for the analysis of educational inequalities in mortality to be extended from the 30–69 or 30–74 age group to the 30+ age group, which will help to paint a more complete picture of educational inequalities. Moreover, the adjusted data aligns—for the total population—with the country-level administrative mortality data. As such, the adjusted data provide a more solid basis for performing further analyses of educational mortality inequalities in E&W.

All things considered, our results indicate the narrowing of absolute educational mortality inequalities among those aged 30 and over in E&W from about 1980 to 2017, and—for men—as well a clear narrowing of relative educational mortality inequalities from 1991 to 2017. This conclusion, based on i) detailed educational data; ii) an internationally commonly used educational classification (low, middle, high); iii) the adjustment of several data issues, and iv) a formal trend analysis is largely in line with the observation of reducing absolute educational inequalities in mortality among those aged 35–79 in E&W from 1990–94 to 2005–09 by Mackenbach et al. 2016 [17] that was received with skepticism [15].

When using our results for the monitoring of trends in socio-economic inequalities in mortality, it needs to be acknowledged that education is but one operationalization of socio-economic status (SES). Moreover, given that SES can be defined as “a person’s position in a hierarchical social structure, encompassing notions of class, status, and power” [42], p. 239) educational attainment and other often used indicators of SES, such as income and occupational class, cannot provide the full story of a person’s socio-economic status, nor all changes therein over an individual’s life. Moreover, educational attainment, unlike income, can only be differentiated in a few levels, and generally only three (low, middle and high educated) are distinguished. Also, the distribution of educational attainment has shifted over time (see Table 1), therefore, being low educated or high educated might indicate something different in terms of SES over time. However, because educational attainment—compared to income and occupation—suffers less from reverse causation and can be measured for people outside the workforce, it is still considered the preferred indicator among the three indicators mentioned above [13].

Previous work on long-term trends in socio-economic mortality inequalities in E&W using information on occupational class (focusing on men) [7, 17, 43], or area-level information on income [44, 45] predominantly reported increasing inequalities. These studies, however, also show that the results depend on the operationalization of socio-economic status [7], on the age groups studied [43, 44], and on how inequalities are measured [43, 45]. For example, Bennett et al. [44] showed that for women aged 0–29 and for men aged 0–59 mortality inequalities between the richest and poorest areas were actually declining between 2001 and 2016. Zazueta et al. [46]—using our adjusted data for E&W—illustrated that the mortality patterns for men aged 65–79 contributed predominantly to the observed decline in the absolute difference in remaining life expectancy at age 30 between low and high educated men from 1976 up to 2008 in E&W. The results also depend on the time period studied. The most recent results from the Office for National Statistics [47] using individual occupation and employment information, show for example that whereas absolute inequalities—measured by SII—in life expectancy at birth increased from 1982–1986 up to 1997–2001 for men, they declined thereafter up to 2012–2016. For women, the inequalities increased from 1982–1986 to 1987–1991, with highly fluctuating levels thereafter. To come to a valid overall conclusion, we recommend—in line with previous research—the study of both absolute and relative inequalities [32, 48], and the use of the SII and RII as the inequality measures [7, 49]. In addition, we recommend i the reliance on a formal trend analysis (e.g. segmented regression; (ii) the interpretation of the results with the socio-economic measure used, age group examined, and period studied in mind; and iii) the examination of the trends in the underlying inequalities for the different age groups.

Based on our work, we make the following recommendations. First, in line with Flanagan and McCartney [14], we consider it undesirable to examine time trends in educational inequalities in E&W that rely only on the main educational variables in the ONS-LS. Instead, we recommend the use of the adjusted data presented in this paper for the study of long-term trends in educational inequalities in mortality for E&W. Second, for the study of trends in educational inequalities in other outcome measures at the aggregate level in E&W (e.g. fertility or health behaviours), we recommend the use of our education classification to researchers who want to adopt a more detailed classification than a binary classification. Third, we suggest the adoption of (part of) our approach for studies on trends in educational inequalities in mortality in other countries facing similar or related issues regarding the consistency over time of educational attainment data, and for studies on trends in other mortality inequalities (e.g., by occupation) for which comparable issues regarding the consistency of the data over time exist. When possible, yearly data should be used to enable the detection of potential inconsistencies in the trends due to data issues.

In general, we believe that the study of educational inequalities in mortality could benefit from more transparency regarding the underlying data used and the underlying choices made (e.g., regarding emigration), and their potential effects on the outcomes of interest. Our study has, for example, illustrated how the use of an inevitably inconsistent definition of emigration would result in substantial discontinuities in trends in the number of personyears and the CDR, both across and by educational groups, unless adjusted for. In contrast, previous studies on educational inequalities in mortality generally did not specify how they dealt with emigration, nor did they discuss the potential bias resulting from it (e.g. [7, 8, 17]). By studying trends over time using annual data, the potential effects of data inconsistencies can be demonstrated. Increased transparency regarding data inconsistencies would raise awareness of the impact of the underlying data and choices on the outcomes studied.

## Conclusions

To conclude, the proposed and implemented adjustment approach illustrates the potential effects of different data issues, and facilitates the study of long-term trends in educational inequalities in mortality in E&W in a time-consistent and internationally comparable manner. Based on the adjusted data, we were able to estimate inequality measures using internationally established methods, and to obtain additional and more detailed insights into past trends in educational inequalities in mortality in E&W. Despite some—inevitable—remaining data issues, the adjusted data provide a more solid basis for the analysis of the main determinants of past trends in educational inequalities in mortality in E&W, and for making inferences for the future. By illustrating the potential effects of different data issues, including those not specific to E&W, we hope to contribute to increased awareness of the data issues and underlying choices involved in the study of educational inequalities in mortality, and their potential impacts on the outcomes studied. Furthermore, we recommend the adoption of (part of) the proposed adjustment approach to identify and/or deal with data-driven inconsistencies in other countries, or beyond the field of educational inequalities in mortality.

## Supplementary Information

**Additional file 1**. Supplementary data and methods.

## Data Availability

The secondary data that we used as input for our analyses cannot be made publicly available, because the data owner (ONS) applies a restricted access policy. The specific output that was used to create the main figures can be obtained through Open Science Framework (https://osf.io/wptgf/). The R codes can be requested from the corresponding author.

## Appendix

See Table 3.

**Table 3:** Our classification of highest educational attainment based on the ONS-LS educational variables

Education*	Highest educational attainment
**Census 1971; ONS-LS variable(s) used**: EDUC7**	
High	Higher university degrees (EDUC7 = 0); Other degrees, and equivalents (EDUC7 = 1)
Middle	A-level and equivalent (EDUC7 = 2); Other qualifications higher than A-level but below degree level (EDUC7 = 3)
Low	None (EDUC7 = 4)
Missing	None stated (EDUC7 = 5)
**Census 1981; ONS-LS variable(s) used: QMLVHIQ8**	
High	Higher degrees of UK standard (QMLVHIQ8 = 1); First degrees and all other qualifications of first degree standard (QMLVHIQ8 = 2)
Middle	Qualifications above GCE (O- and A-levels) but not first and higher degrees (QMLVHIQ8 = 3)
Low	No qualifications obtained since the age of 18 (QMLVHIQ8 = − 9)
Missing	Highest qualification is not stated (QMLVHIQ8 = 4)
**Census 1991; ONS-LS variable(s) used: QMLVHQT9 and QMQUAL19**	
High	Higher degrees of UK standard (QMLVHQT9 = 1); First degrees and all other qualifications of first degree standard (QMLVHQT9 = 2)
Middle	Qualifications above GCE (O- and A-levels) but not first and higher degrees (QMLVHQT9 = 3)
Low	No qualifications obtained since the age of 18 excluding qualifications normally obtained at school (QMQUAL19 = − 9)
Missing	Rest of “not applicable or missing” (Remaining of QMLVHQT9 = − 9)
**Census 2001; ONS-LS variable(s) used: HLQP0, and QUP0 and PQUP0**	
High	First Degree (e.g., BA,BSc) (QUP0_5 = 1); Higher Degree (e.g., MA, PhD, PGCE, post-graduate certificates/diplomas) (QUP0_6 = 1); NVQ Level 4–5, HNC, HND (QUP0_10 = 1)
Middle	Upper-level secondary qualifications, such as A-levels and advanced GNVQ (HLQP0 = 13); Professional qualifications (qualified teachers, medical doctors, dentists, nurses) without a degree (qualprofdegree = 0); Other academic (e.g., City and Guilds, RSA/OCR, BTEC/Edexcel) and professional qualifications (HLQP0 = 15)
Low	No academic or professional qualifications (HLQP0 = 10); Qualifications below upper level secondary education, such as CSEs and GCSEs (HLQP0 = 11; HLQP0 = 12)
Missing	Missing (HLQP0 = − 7); Not applicable (HLQP0 = − 9)
**Census 2011; ONS-LS variable(s) used: HLQP11 and QUP11**	
High	Degree (e.g., BA, BSc) and higher degree (e.g., MA, PhD, PGCE) (QUP11_8 = 1); NVQ Level 4–5, HNC, HND, RSA Higher Diploma, BTEC Higher Level (QUP11_9 = 1)
Middle	Apprenticeships (HLQP11 = 13); Upper-level secondary qualifications, such as A-levels, advanced GNVQ, advanced Baccalaureate (HLP11 = 14); Professional qualifications without a degree (QUP11_10 = 1 & QUP11_8 = 0 & QUP11_9 = 0); Other: vocational/work-related qualifications, foreign qualifications/qualifications gained outside the UK (NI) (Not stated/level unknown) (HLQP11 = 16)
Low	No qualifications (HLQP11 = 10); Qualifications below upper-level secondary educations, such as CSEs and GCSEs (HLQP11 = 11; HLQP11 = 12)
Missing	Missing (HLQP11 = − 6); No code required (HLQP11 = − 9)

Source information: ONS-LS

*Low = no, pre-primary, primary, and lower secondary education (ISCED-1997 0–2); middle = upper secondary and post-secondary non-tertiary education (ISCED-1997 3–4); high = tertiary education (ISCED-1997 5–6) [23]. ** Additional file 1: Table S1 lists the summary ONS-LS educational variables (EDUC7, QMLVHIQ8, QMLVHQT9, HLQP0, HLQP11); Appendix II of the Additional file 1: Appendix II l lists the additional educational variables used

## Abbreviations

E&W: England & Wales
ONS-LS: Office for National Statistics Longitudinal Study
SDR: Age-standerdised death rate
CDR: Crude death rate
SII: Slope index of inequality
RII: Relative index of inequality
RD: Rate difference
MRR: Mortality rate ratio
