# Supplementary material for: Enabling the examination of long-term mortality trends by educational level for England and Wales in a time-consistent and internationally comparable manner
Source: Popul Health Metr. 2024 Mar 9;22:4. doi: 10.1186/s12963-024-00324-2 (PMC10925007; doi:10.1186/s12963-024-00324-2)

Enabling the examination of long-term mortality trends by educational level for England and Wales in a time-consistent and internationally comparable manner

Supplementary data and methods

CONTENTS

1.	DATA	2
2.	THE MAIN DATA ISSUES	3
2.1	The main data issues summarized	3
2.2	Inconsistent information on educational attainment over time	4
2.2.1	Questions, possible answers, and variables pertaining educational attainment by census	4
2.2.2	Inconsistencies in educational classification across the censuses	11
2.3	Large number of (elderly) people with missing educational level	12
2.4	Challenges in defining emigrants in a consistent way across the censuses	12
2.5	Imperfect and time-varying alignment with administrative country-level data	16
3.	APPROACH AND METHODS	17
3.1	Our approach	17
3.2	Methods	18
4.	THE DIFFERENT ELEMENTS OF OUR APPROACH AT THE INDIVIDUAL LEVEL	19
4.1	Our educational classification	19
4.2	Dealing with missing educational information (individual level)	24
5.	THE DIFFERENT ELEMENTS OF OUR APPROACH AT THE AGGREGATE LEVEL	26
5.1	Initial steps	26
5.1.1	From individual-level cohort data to aggregate period data	26
5.1.2	Smoothing	28
5.2	Proportional redistribution of missings	34
5.3	Careful study of trends over time	40
5.4	Adjustment of the trend break in 1981	43
5.5	Correcting for the inevitably inconsistent definition of emigration over time	54
5.6	Alignment of our ONS-LS data selection with administrative country-level data	59
6.	SYNTHESIS	67
6.1	Appraisal of the assumptions	67
6.2	After all adjustments	68
	REFERENCES	74
	APPENDIX I	76
	APPENDIX II	78
	APPENDIX III	81

1. DATA

To study mortality trends by educational level (30+) for England & Wales it is desirable to have data for the longest time series possible that is representative for the total ENW population. To avoid denominator-numerator bias, the obtainment of such data requires the linkage of individual death records with individual information on educational level.

We used information from the Office for National Statistics Longitudinal Study (ONS-LS) (Office for National Statistics, 2019), which comprises individually linked data for a 1% representative sample of the population of England and Wales from 1971 onwards (see Shelton et al., 2019). The ONS-LS members were initially drawn from respondents to the 1971 Census born on one of four undisclosed birthdays per year. Subsequent samples were drawn from the 1981, 1991, 2001 and 2011 censuses based on the same four birthdays. The ONS-LS comprises not only information on demographic and socio-economic variables obtained through the censuses, but also information on important life events through linkages to registrations of – among others – births, deaths, immigration and emigration. Between censuses, new births and immigrants are added to the LS if they were born on one of the same four birthdays, and individuals exit the study through death or emigration.

We used the ONS-LS data because it covers a long series of mortality data by calendar year and individual data linkage is already performed. Moreover, different from for example the General Household Surveys and Labour Force Surveys, the data covers the whole population (including those in institutions) and non-response is considerably lower because of the reliance on census data. In contrast to the British birth cohort studies, which follow a certain birth cohort (= those born in a certain year) over time, the ONS-LS essentially follows people of all ages in 1970, as well as people of all ages in subsequent samples related to subsequent census years. See Shelton et al., 2019.

We used ONS-LS data (i.e. birth date, death date, highest educational attainment at the time of the censuses, emigration) from 1971 – 2017 and performed 10-year follow ups (slightly shorter for the last census) of the ONS-LS members aged 20 and older at the time of the census in 1971 (April 25), 1981 (April 5), 1991 (April 21), 2001 (April 29), and 2011 (March 27). We followed individuals until the next census, or the date that vital status information was last linked to the LS (currently December 31, 2017).

We utilized a semi-closed cohort. That is, immigrants were not counted until the first census after they arrived in England and Wales to ensure that information on their educational level was available. For those who emigrated but returned during the same follow-up period, the person time for all ten years was counted (or, in case they died, until their date of death). Emigrants who did not return during the same follow-up (i.e. were not present at the following census) were excluded from the entire follow-up period.

In line with previous international comparative research (e.g. de Gelder et al. 2017), we ideally would like to distinguish three highest educational attainment groups (low, middle and high) using the International Standard Classification of Education (ISCED) (UNESCO, 1997). 'Low' is used for no, pre-primary, primary and lower secondary education (ISCED 0-2), 'Middle' is used for upper secondary and post-secondary non-tertiary education (ISCED 3-4), and 'High' is used for tertiary education (ISCED 5-6) (e.g. de Gelder et al. 2017).

Once we had defined the educational variable in line with the above classification, we rearranged the individual-level cohort data (20+) for the five different follow-up periods (1971-1981, 1981-1991, 1991-2001, 2001-2011, 2011-2017) into aggregate period data by education level, sex and single year of age (30+) for single calendar years from 1972 up to 2017.

2. THE MAIN DATA ISSUES

Despite clear advantages of using the ONS-LS for our study, the ONS-LS data – and in particular the decennial census data used to allocate people to educational groups – has some issues (e.g. Flanagan & McCartney 2015).

The main data issues – that are potentially solvable - are summarized in section 2.1 and discussed in more detail in sections 2.2-2.5.

Additional data issues include a) general issues regarding the use of decennial census data – compared to more in-depth annual information - to obtain educational information; b) other - smaller - inconsistencies in the framing of the England and Wales census questions regarding education over time; c) other general issues that could affect the consistency of educational information over time, including changes over time in the education system (e.g. changes in the number of years needed to obtain a certain degree); changes in the acquiring of qualifications (e.g. exam based or not); and changes in the valuation of qualifications (e.g. a nursing qualification obtained in the 1970s is valued differently than a nursing qualification acquired in the 2000s).

2.1 The main data issues summarized

The following data issues are hampering the construction of consistent time trends in mortality by educational level (low, middle, high) for England and Wales (30+) in 1972-2017:

1) **Across the censuses, the questions and variables regarding education vary, making the information on educational attainment inconsistent over time.** Firstly, because in the 1981 and 1991 censuses, respondents were asked to disregard any qualifications normally obtained at school, individuals who did obtain an upper-level secondary education qualification (the cut-off between low and middle educated), but no other post 18 qualification, were misclassified as low educated. Secondly, the main education variable in the 1991 Census does not distinguish a code for no qualifications (= low educated), but only one code for "not applicable or missing" combined. Thirdly, the classification of those with professional qualifications but not a degree is not consistent across the censuses. Whereas in 1971, 1981 and 1991 they are classified as sub-degree level (= middle educated), in 2001 (except for "other professional qualifications") and in 2011 they are classified as degree level (= high educated). Fourthly, in 2001 and 2011, a separate category for "other" exists, which also represents slightly different things in 2001 and 2011. In comparison to the ISCED, the education information from the main education variables in ONS-LS align rather well in 1971, slightly less in 2001 and 2011 (because of the "other" group), and not well (except for the high educated) in the 1981 and 1991 censuses (UNESCO, 1997; Schneider, 2008; OECD, 2022).
2) **Information on educational attainment is not in all censuses available for all adults, resulting in important missing educational information.** Respondents aged 70 and older in 1971 and aged 75 and older in 2001 were, respectively, not required to answer the question regarding their educational level or not asked about their educational level. In addition, particularly in the 1971 Census, nonresponse among non-elderly respondents resulted in missing educational information.
3) **Emigration could – inevitably - not be defined consistently over time.** When exact dates of emigration are not available for the whole time series, emigrants are generally excluded from the different follow-up periods to avoid biased estimates of the personyears at risk. When – furthermore - reporting of emigrants is considered to be poor, individuals are ideally defined as emigrants if they were not present at the beginning of the next follow up (= mostly the next census) and did not die. If, for the last follow-up period, data for the next census is not yet available, this definition cannot be applied for the last follow-up period, which creates both inconsistencies in the time series, and potentially biased estimates for the last follow-up period.

In E&W, official emigration statistics with emigration dates are considered incomplete (CeLSIUS, 2021). Therefore we also had to rely on the inconsistent definition of emigration over time. More specifically, for the final follow-up period (2011-2017), we could only define, and subsequently exclude, emigrants based on the available data in the ONS-LS, which stem from the registered embarkations (emigrations) data from the NHS Central Register (NSHCR). However, it is estimated that only 50% of emigrations are reported to the NHSCR (CeLSIUS, 2021).

4) **The alignment of our ONS-LS data selection with country-level population and mortality data for the general E&W population is imperfect and changes over time, resulting in issues regarding the national representativeness of our outcomes.** First, the increases in the sample size of the ONS-LS have not been in line with increases in the size of the national population of E&W. Second, our modifications to the data to avoid bias (e.g. the exclusion of emigrants (see previous point), and not counting immigrants until the census after they arrived in E&W to ensure complete information on educational attainment), resulted in additional differences with country-level data.

2.2 Inconsistent information on educational attainment over time

The ONS-LS obtains information on the educational attainment from the censuses. The questions, target audience, and possible response categories regarding education differ from one census to another and are not always convertible to the internationally used ISCED.

2.2.1 Questions, possible answers, and variables pertaining educational attainment by census

Below we provide information regarding the wording of the questions, possible answers to the questions, target audience, and constructed variables by ONS-LS for educational attainment by census. This information has been compiled from the information available at the website of CeLSIUS (Centre for Longitudinal Study Information and User Support) (CeLSIUS, 2022a) and the census forms available on the CALLS Hub (CALLS-Hub, 2022). See Appendix I for screenshots of the questions in the census form itself.

<u>Census 1971</u>
The household member who responds to the census was asked two questions regarding educational attainment of household members aged 15 and older in their household. A special note was added for people aged over 70: *"If you are over 70 and retired you need not answer any questions on this page [..]. If you had a job last week all questions 13-22 should be answered"*.

The first question, B13, was *"Have you obtained any of the following?"* with as answer possibilities*:*

GCE 'A' level or Higher School Certificate (HSC)
Higher Grade of Scottish Certificate of Education (SCE) or Higher Grade of Scottish Leaving Certificate (SLC)
Ordinary National Certificate (ONC) or Ordinary National Diploma (OND)
None of these

The second question, B14, was formulated as *"Have you obtained **any** of the following qualifications since reaching the age of 18?"* and the answer possibilities were:

HNC or HND
Nursing qualifications
Teaching qualifications
Degrees, diplomas or other educational qualifications
Graduate or corporate membership of professional institutions
Any other professional or vocational qualifications

*If so, give full details of **all** such qualifications in the order in which they were obtained, even if not relevant to your present job or if you are not working. If none, write 'NONE'."* The instructions for the

interviewers were: *"Please check these details by asking each person about his qualifications. This question need not to be answered for persons under 18 or retired persons over 70."*

Based on the replies to these two questions, the main education variable EDUC7 was generated in the ONS-LS, named "Academic level of LS member. 1971", containing the following categories:

Higher university degree
Other degrees and equivalent
Other qualifications higher than A levels
A level and equivalent
None
None stated

Census 1981 and 1991

In 1981 and 1991, the upper age limit of 70 has been removed. Furthermore, the questions about educational attainment changed compared to 1971. In 1981 and 1991 only one question about educational attainment was asked, question 16 in 1981 and question 19 in 1991:

Degree, professional, and vocational qualifications
Have you obtained any qualifications after the age of 18 such as:

Degree, Diplomas, HNC, HND
Nursing qualifications, Teaching qualifications
Graduate or corporate memberships of professional institutions
Other professional, educational or vocational qualifications?

Exclude qualifications normally obtained at school such as GCE, CSE, GCSE, SCE and School Certificates.
The response options were *"No"* (box 1) and *"Yes"* (box 2).
If box 2 is ticked write in all qualifications even if they are not relevant to your present job or if you are working. Please list the qualifications in the order in which they were obtained.
Write for each qualification:

The title
The major subject or subjects
The year obtained
The awarding institution

The information obtained through this question was used within ONS-LS to construct the educational variables: HIQAL8 and HIQAL9, and QMLVHIQ8 and QMLVHQT9, where 8 represents 1981 and 9 represents 1991.

HIQAL8 and HIQAL9 contain a list of all qualifications mentioned by census members which were broadly divided into three classes: level a (Higher degrees of UK standard), level b (First degrees and all other qualifications of first degree standard), and level c (qualifications that are generally obtained at 18 and older and that are above GCE A-levels (= General Certificate of Education, which is the main school leaving examination in England, Wales, Northern Ireland, the Channel Islands and the Isle of Man, generally taken at age 18 and used by universities in England, Wales, and Northern Ireland as the standard for assessing the applicants' suitability for admission)).

The main education variable QMLVHIQ8 is purely based on the information in HIQAL8 and consists of the following categories:

Highest qualification is level 'a' (=HIQAL8 001-007)
Highest qualification is level 'b' (=HIQAL8 008-255)
Highest qualification is level 'c' (=HIQAL8 256-478)
Highest qualification is not stated
Person aged under 16 or has no qualifications.

The main education variable QMLVHQT9 is purely based on the information in HIQAL9 and consists of the following categories:

Level 'a' (=HIQAL9 001-007)
Level 'b' (=HIQAL9 008-255)
Level 'c' (=HIQAL9 256-478)
Not applicable or missing

The levels a, b, c in 1991 are identical to those in 1981.

Census 2001

In 2001, respondents were asked to answer questions regarding educational attainment for individuals aged 16 to 74 in their household. Several multi-tick questions were asked to identify all qualifications obtained. In England and Wales, a distinction was made between educational and professional qualifications.

Question 16 asks the respondent *"Which of these qualifications do you have"*, thereby listing twelve distinctive educational qualifications (see below). Respondents are asked to *"Tick all boxes that apply or, if not specified, the nearest equivalent".*

1+ O levels/CSE/GSCE (any grades)
5+ O levels, 5+ CSEs (grade 1), 5+ GSCEs (grades A-c), School Certificate
1+ A levels/AS levels
2+ A levels, 4+ AS levels, Higher School Certificate
First Degree (eg BA, BSc)
Higher Degree (eg MA, PhD, PGCE, post-graduate certificates/diplomas)
NVQ level 1, Foundation GNVQ
NVQ level 2, Intermediate GNVQ
NVQ level 3/Advanced GNVQ
NVQ level 4-5, HNC, HND
Other qualifications (eg City and Guilds, RSA/OCR, BTEC/Edexcel)
No qualifications

The answers to the 12 educational qualifications from question 16 were recorded as variable QUP0 in ONS-LS. This variable has twelve positions in line with the abovementioned 12 educational qualifications. A "0" for a position indicates that the person does not have the particular educational classification. A "1" for a position indicates that the person has the particular educational classification. For example, if a respondent had ticked the boxes for "2+ A Levels, 4+ AS levels, Higher School Certificate" and "First degree (eg BA, BSc)", their entry in QUP0 as "000110000000".

Question 17 of the 2001 census asks *"Do you have any of the following professional qualifications"*. Six professional qualifications (see below) are listed for which people are asked to *"Tick all the boxes that apply."*

No Professional Qualifications
Qualified Teacher Status (for schools)
Qualified Medical Doctor
Qualified Dentist
Qualified Nurse, Midwife, Health Visitor
Other Professional Qualifications

The answers to the six professional qualifications in question 17 recorded as variable PQUP0. PQUP0 has six positions. For the first five positions, a 1 indicates that the professional qualification applies. For the last position, the following coding applies, 0 Has no other Professional Qualifications 1 Has other professional Qualifications 2 Not applicable.

The main education variable HLQP0 (“highest qualification”) combines the information on educational qualifications in QUP0 and professional qualifications in PQUP0 to identify the highest qualification obtained. The levels distinguished in HLQP0 are an indication of the NVQ framework used to classify qualifications in the UK. The following categories are identified:

- No academic or professional qualifications
- Level 1: CSEs (grades 2-5), GSCEs (grades D-G), 1-4 CSE (grades 1), 1-4 GSCEs (grades A-C), 1-4 O Levels, NVQ Level 1, Foundation GNVQ
- Level 2: 5+O levels, 5+CSEs (grade1), 5+GCSEs (grades A-C) etc, 1 A level, 1-3 AS levels, NVQ level 2, Intermediate GNVQ
- Level 3: 2+ A levels, 4+ AS levels, Higher School Certificate, NVQ level 3, Advanced GNVQ
- Level 4/5: First degree, Higher degree, NVQ levels 4-5, HNC, HND. Qualified Teacher status, Qualified Medical Doctor, Qualified Dentist, Qualified Nurse, Midwife, Health Visitor
- Other qualifications/ level unknown: Other qualifications (eg City and Guilds etc), Other Professional qualifications
- -7 Missing
- -9 Not applicable

First the information from QUP0 is used to identify the highest educational qualification. Second, the information from PQUP0 is used to assess whether the highest professional qualification was either 1) a Qualified Teacher status, Qualified Medical Doctor, Qualified Dentist, or Qualified Nurse, Midwife, Health Visitor, or 2) Other professional qualification. All people with highest professional qualification = 1 were assigned code 14 (“Level 4/5”). Only people with no educational qualification but with “other professional qualifications” were assigned code 15 (“Other qualifications / level unknown”).

<u>Census 2011</u>
For all respondents aged 16 and older a single tick box question, question 25, regarding qualifications needed to be answered.
The question was: *Which of the following qualifications do you have?*
*Tick **every** box that applies if you have **any** of the qualifications listed. If your UK qualification is not listed, tick the box that contains its nearest equivalent. If you have qualifications gained outside the UK, tick the ‘Foreign qualifications’ box and the nearest UK equivalents (if known).*

The following 13 options were listed:

- *1-4 O levels/CSEs/GCSEs (any grades), Entry Level, Foundation Diploma*
- *NVQ Level 1, Foundation GNVQ, Basic Skills*
- *5+ O levels (passes)/CSEs (grade 1)/GCSEs (grades A-C), School Certificate, 1 A level/2-3 AS levels/VCEs, Higher Diploma*
- *NVQ Level 2, Intermediate GNVQ, Basic Skills*
- *Apprenticeship*
- *2+ A levels/VCEs, 4+ AS levels, Higher School Certificate, Progression/Advanced Diploma*
- *NVQ Level 3, Advanced GNVQ, City and Guilds, Advanced Craft, ONC, OND, BTEC National, RSA Advanced Diploma*
- *Degree (for example BA, BSc), Higher degree (for example MA, PhD, PGCE)*
- *NVQ Level 4-5, HNC, HND, RSA Higher Diploma, BTEC Higher Level*
- *Professional qualifications (for example teaching, nursing, accountancy)*
- *Other vocational/work-related qualifications*
- *Foreign qualifications*
- *No qualifications*

The answers were used to generate two variables pertaining to qualifications in ONS-LS: QUP11 and HLQP11.

QUP11 has 13 positions, and similarly to QUP0 each position corresponds with the qualification outlined above. A “1” for a position indicates that the respondent has the particular qualification.

The information in QUP11 was then summarised to derive the main education variable HLQP11 (“highest qualification. 2011”). The main education variable HLQP11 contains the following categories:

No academic or professional qualifications (England & Wales & Northern Ireland)

Level 1: 1-4 O Levels/CSE/GCSEs (any grades), Entry Level, Foundation Diploma, NVQ level 1, Foundation GNVQ, Basic/Essential Skills (England & Wales & Northern Ireland)

Level 2: 5+ O Level (Passes)/CSE (Grade 1)/GCSE (Grades A-C), School Certificate, 1 A Level/2-3 AS Levels/VCEs, Intermediate/Higher Diploma, Welsh Baccalaureate, Intermediate Diploma, NVQ level 2, Intermediate GNVQ, City and Guilds Craft, BTEC First/G

Apprenticeship (England & Wales & Northern Ireland)

Level 3: 2+ A Levels/VCEs, 4+ AS Levels, Higher School Certificate, Progression/Advanced Diploma, Welsh Baccalaureate Advance Diploma, NVQ Level 3; Advanced GNVQ, City and Guilds Advanced Craft, ONC, OND, BTEC National, RSA Advanced Diploma (England & Wales & Northern Ireland)

Level 4+: Degree (BA, BSc), Higher Degree (MA, PhD, PGCE), NVQ Level 4-5, HNC, HND, RSA Higher Diploma, BTEC Higher level, Foundation Degree (NI), Professional Qualifications (Teaching, Nursing, Accountancy) (England & Wales & Northern Ireland)

Other: Vocational/Work-related Qualifications, Foreign Qualifications/ Qualifications gained outside the UK (NI) (Not stated/level unknown) (England & Wales & Northern Ireland)

-6 Missing

-9 No code required (Aged less than 16, Students not at their term-time address)

An overview of the main educational variables in ONS-LS (EDUC7, QMLVHIQ8, QMLVHQT9, HLQP0, HLQP11) can be found in Table S1. Most research on educational differences using the ONS-LS study, that did not merely use a binary educational variable, use these main educational variables (e.g. Plewis & Bartley, 2014; Ingleby et al., 2021).

Table S1. Overview of the summary educational variables in ONS-LS based on the census questions, by census year, including the variable name, coding, and category definition

Census year	Variable name	Coding	Category definition
1971	EDUC7	0	Higher university degree
		1	Other degrees and equivalents
		2	Other qualifications higher than A level
		3	A level and equivalents
		4	None
		5	None stated
1981	QMLVHIQ8	1	Highest qualification is level ‘a’: Higher degrees of UK standard
		2	Highest qualification is level ‘b’: First degrees and all other qualifications of first-degree standard
		3	Highest qualification is level ‘c’: Qualifications that are generally obtained at 18 and older and are above GCE
		4	Highest qualification is not stated
		-9	Person aged under 16 or has no qualifications
1991	QMLVHQT9	1	Level ‘a’: Higher degrees of UK standard
		2	Level ‘b’: First degrees and all other qualifications of first-degree standard
		3	Level ‘c’: Qualifications that are generally obtained at 18 and older and are above GCE
		-9	Not applicable or missing
2001	HLQP0	10	No academic or professional qualifications
		11	Level 1: CSEs (grades 2-5), GSCEs (grades D-G), 1-4 CSE (grades 1), 1-4 GSCEs (grades A-C), 1-4 O Levels, NVQ Level 1, Foundation GNVQ
		12	Level 2: 5+O levels, 5+CSEs (grade1), 5+GCSEs (grades A-C) etc, 1 A level, 1-3 AS levels, NVQ level 2, Intermediate GNVQ
		13	Level 3: 2+ A levels, 4+ AS levels, Higher School Certificate, NVQ level 3, Advanced GNVQ
		14	Level 4/5: First degree, Higher degree, NVQ levels 4-5, HNC, HND. Qualified Teacher status, Qualified Medical Doctor, Qualified Dentist, Qualified Nurse, Midwife, Health Visitor
		15	Other qualifications/ level unknown: Other qualifications (eg City and Guilds etc), Other Professional qualifications
		-7	Missing
		-9	Not applicable
2011	HLQP11	10	No academic or professional qualifications (England & Wales & Northern Ireland)
		11	Level 1: 1-4 O Levels/CSE/GCSEs (any grades), Entry Level, Foundation Diploma, NVQ level 1, Foundation GNVQ, Basic/Essential Skills (England & Wales & Northern Ireland)
		12	Level 2: 5+ O Level (Passes)/CSE (Grade 1)/GCSE (Grades A-C), School Certificate, 1 A Level/2-3 AS Levels/VCEs, Intermediate/Higher Diploma, Welsh Baccalaureate, Intermediate Diploma, NVQ level 2, Intermediate GNVQ, City and Guilds Craft, BTEC First/G
		13	Apprenticeship (England & Wales & Northern Ireland)

14	Level 3: 2+ A Levels/VCEs, 4+ AS Levels, Higher School Certificate, Progression/Advanced Diploma, Welsh Baccalaureate Advance Diploma, NVQ Level 3; Advanced GNVQ, City and Guilds Advanced Craft, ONC, OND, BTEC National, RSA Advanced Diploma (England & Wales & Northern Ireland)
15	Level 4+: Degree (BA, BSc), Higher Degree (MA, PhD, PGCE), NVQ Level 4-5, HNC, HND, RSA Higher Diploma, BTEC Higher level, Foundation Degree (NI), Professional Qualifications (Teaching, Nursing, Accountancy) (England & Wales & Northern Ireland)
16	Other: Vocational/Work-related Qualifications, Foreign Qualifications/ Qualifications gained outside the UK (NI) (Not stated/level unknown) (England & Wales & Northern Ireland)
-6	Missing
-9	No code required (Aged less than 16, Students not at their term-time address)

Source information: ONS-LS

2.2.2 Inconsistencies in educational classification across the censuses

As outlined above, the questions and variables regarding educational attainment vary across censuses. In particular, four issues are hampering the construction of a consistent educational classification in line with the ISCED classification (low: ISCED 0-2, middle: ISCED 3-4, and high: ISCED 5 and above), particularly when solely relying on the main educational variables in the ONS-LS (EDUC7, QMLVHIQ8, QMLVHQT9, HLQP0, HLQP11).

First, it is difficult to distinguish ONS-LS members with low and middle educational status in the 1981 and 1991 censuses. In 1981 and 1991, respondents were asked to disregard any qualifications before the age of 18 and thus normally obtained at school. However, obtaining an upper-secondary school qualification is the cut-off between the low and middle educated. Respondents instead were asked whether they obtained any qualifications after the age of 18, excluding qualifications normally obtained at school. Therefore, only a select group of middle educated individuals who obtained a sub-degree level qualification after turning 18 and leaving school could be identified in 1981 and 1991. Consequently, individuals who obtained an upper-level secondary education qualification but no other post-18 qualification could have been misclassified as low educated.
Second, the main education variable in the 1991 census (QMLVHQT9) does not distinguish a code for "no qualifications" (= low educated), but only one code for "not applicable or missing" combined.

Third, the classification of professional qualifications is not consistent across the censuses. In the main education variables in 1971, 1981 and 1991, people with a professional qualification - such as teacher, medical doctor, dentist, nurse, midwife, health visitor – but without a degree were categorised as sub-degree level, which would translate to middle educated in line with the ISCED classification. In the main education variables in 2001 and 2011 all people with professional qualifications, including those without a degree, are coded as NVQ Level 4/5, which would be regarded high educated according to the ISCED classification. This may be due to changes in the qualification process of some of these professions. For example, nurses could register with a diploma or degree level qualifications until 2009, when it became an all-degree profession, and from 2013 onwards all newly registered nurses require a degree level education (Shields & Watson, 2007; Coleman, 2021). Nevertheless, the classification of professional qualifications without a degree is inconsistent over time, and – for 2001 and 2011 - not consistent with the ISCED classification (Eurostat, 2022; OECD, 2022).

Fourthly, in 2001 and 2011, the main educational variables distinguish not only a missing category but also a separate category "other". Of the ONS-LS members aged 20 to 74, 7.44% and 6.44% in 2001 and 2011 respectively are categorised as "other" under the main educational variables. This poses a challenge when re-classifying this group of ONS-LS members according to the ISCED 1997 classification. Moreover, the group "other" represents slightly different things in 2001 and 2011; in 2001 it includes those with "other professional qualifications" without a degree, whereas in 2011 this group is not separately distinguished as part of "other".

In comparison to the ISCED, the educational information from the main education variables in ONS-LS aligns rather well in 1971. The 1971 classification distinguishes between tertiary, secondary, and no qualifications, based on the highest academic qualifications. The educational information from the main educational variables in 1981 and 1991 does not align well with the ISCED due to the difficulty in distinguishing between those with low and with middle educational attainment. The educational information that can be obtained from the main educational variables in 2001 and 2011 align well with ISCED overall. That is, the main educational variables in 2001 and 2011 use – for the academic qualifications - the NVQ classification commonly used by English researchers to define the English educational system (CeLSIUS, 2022b) in which it is easy to distinguish people with a tertiary degree and upper secondary qualifications. Still, as mentioned, their use of a separate category "other" and

their way of classifying those with professional qualifications without a degree, warrants our attention.

All aforementioned issues pose challenges when constructing a consistent educational classification in line with the ISCED classification. These challenges have also been discussed by Flanagan & McCartney (2015) and McCartney (2016) and have resulted in the less optimal study of educational inequalities in mortality over time and between countries. For example, a previous study that specifically aimed at studying long-term trends in educational inequalities in mortality for different European countries, focussed – for England and Wales – purely on comparing the periods 1971-1981 and 2001-2009 (e.g. de Gelder et al. 2017), because "in the datasets for 1981–1991 and 1991–2001 in England and Wales only two levels of education could be distinguished ('low and middle' vs 'higher')". Gregoraci et al. (2017) purely distinguished those with ISCED 0-3 from those with ISCED 4-6 for England & Wales in the 1990-1994 period. This will likely not result in similar outcomes when distinguishing the low, middle and high educated, because mortality trends in Europe generally follow distinct trajectories between low and middle educated groups (de Gelder et al. 2017).

2.3 Large number of (elderly) people with missing educational information

Another issue with the information on educational attainment in the ONS-LS (which is based on the educational information in the censuses) is that it is not available for all adults. Respondents aged 70 and older in 1971 and aged 75 and older in 2001 were, respectively, not required to answer the education question or not asked about their educational level (see section 2.2.1). As a result, no information regarding educational level exists for individuals aged 75+ in 2001, and 33% of the elderly (70+) in 1971 did not report or were not asked about their educational level (see Table S2).

In addition, particularly in the 1971 Census, nonresponse among non-elderly respondents resulted in missing educational information (3.43% of the respondents aged 20-69 in 1971 (11,156 LS members)) (see Table S2). In the 1981 and 1991 censuses missing educational information was negligible (0.01% aged 20 and older in 1981; 0.42% aged 20 and older in 1991). For the 2001 and 2011 censuses, the qualification variables were imputed by ONS (Office for National Statistics, 2012a), resulting as well in negligible missing educational information (0.32% aged 20-74 in 2001; 0.28% aged 20 and older in 2011). Note that this imputation procedure was not applied to those aged 75 and older in 2001, because they were not meant to answer the qualification questions.

Table S2. Number of people with missing educational information at the time of the decennial censuses, and their frequencies

Age	1971	1981	1991	2001	2011
% aged 30 and older	7.88 (23,170)	0.01 (36)	0.30 (966)	12.27 (42,063)	0.04 (135)
% aged 20 and older	6.86 (25,324)	0.01 (41)	0.42 (1,697)	10.56 (43,119)	0.28 (1,248)
% aged 30-69 / 30-74*	2.67 (9,002)	0.01 (23)	0.25 (896)	0.03 (103)	0.03 (115)
% aged 20-69 / 20-74**	3.43 (11,156)	0.01 (28)	0.44 (1,627)	0.32 (1,159)	0.30 (1,228)
% aged 70 plus / 75 plus***	32.62 (14,168)	0.02 (13)	0.18 (70)	100 (41,960)	0.04 (20)

Source data: ONS-LS. * 30-69 for 1971 and 1981; 30-74 for 1991, 2001, 2011. ** 20-69 for 1971 and 1981; 20-74 for 1991, 2001, 2011. *** 70+ for 1971 and 1981; 75+ for 1991, 2001, 2011.

In previous research on educational inequalities in mortality, non-elderly respondents with missing educational information were generally omitted, although this is not always specified. This results in less robust numbers, particularly for the 1971 Census. Also, individuals aged 70 or 75 and older

(depending on the time span covered) are omitted from previous research on educational inequalities in mortality covering England & Wales (e.g. Flanagan & McCartney 2015; de Gelder et al. 2017). Consequently, long-term trends in socio-economic mortality inequalities for England & Wales has purely been investigated for those up to age 69 or 74, and not for individuals aged 75 and older. This is problematic because the effects of socio-economic differences on mortality extend into these higher ages (Huisman 2004), and socio-economic differences in mortality at these ages have a larger impact on life expectancy because of the concentration of deaths towards older ages (van Baal et al. 2016).

2.4 Challenges in defining emigrants in a consistent way across the censuses

An important choice to make in a mortality follow-up is how to deal with emigration. Ideally, emigrants would be included in the exposure time until they leave the country. However, the exact date of emigration is generally not available, or not available for the entire study period. Furthermore, because reporting of emigration is often incomplete (UNECE, 2009), relying on the official registration of emigration could result in an underestimation of emigrants, an overestimation of personyears, and consequently possibly an underestimation of mortality rates. Therefore, an emigrant is instead generally defined as someone who was not present at the beginning of the next follow up (generally the next census) and did not die, and is excluded from the particular follow-up. However, when data for the beginning of the next follow up (i.e. the most recent census) is not available yet, it is not possible to define emigrants in this way for the last follow-up period. Including the data for the final follow up period, therefore requires either a suboptimal emigration definition altogether, or working with a different emigration definition for the final follow-up period compared to the previous follow-up periods.

For England and Wales, the reporting of emigration is far from complete (CeLSIUS, 2021). In England & Wales, emigration notifications are sent to the NHS Central Register (NSHCR) when individuals that have a national insurance or receive benefits or pensions are known to be leaving the country, or if an individual informs their GP that they are emigrating. The latter is not a legal requirement and therefore embarkations are not all notified to the NHSCR, or, if so, with a potential delay. It is estimated that only 50% of emigrations are notified to the NHSCR (CeLSIUS, 2021). Because of this large underestimation of emigrations, we choose to define emigrants as those who were not present at the next census and did not die during the relevant 10-year follow-up period. However, for the last follow-up period (2011 onwards), in the absence of data from the next census[1], we could only define emigrants based on the available data on emigration in the ONS-LS (through the "embarkation" data table), which is based on emigrations that are part of the NSHCR. This inevitably inconsistent definition of emigration means that from 2011 onwards emigration is likely to have been underestimated.

In fact, based on a comparison of number of emigrants using both emigration definitions (1: based on absence in next census; 2: based on embarkation data) for the follow-up periods before 2011 (see Table S3), the underestimation of emigrants in the 2011—2017 follow-up is substantial. That is, in 2001-11 the underestimation of emigrants when we would have used the emigration definition based on embarkation (EMI2) instead of the emigration definition based on presence in the next census (EMI1) would amount to 38,533 – 3,157 = 35,376.

Multiplying the 2001 – 2011 ratio of the number of individuals not enumerated in the subsequent census (EMI2) to the number of reported embarkations (EMI1) (12.19), with the 3,060 reported embarkations in the 2011-2017 follow-up period would mean a preliminary estimated number of individuals not enumerated in the subsequent census of 37,301, and, consequently, an underestimation of 34,241 (37,301 – 3,060) emigrants in the 2011-2017 follow-up period. These 34,241 underestimated emigrants, and consequently overestimated persons in the 2011-2017 follow-

[1] The 2021 Census was undertaken on 21/03/2021 but has not been linked to the ONS-LS yet

up comprises approximately 8.5 percent (34,241/403,695) of individuals aged 20 and older in the 2011-2017 follow up.

Table S3. Comparison of the number of emigrants using two different definitions, in each follow-up period for the ONS-LS population aged 20 and over

Follow-up	EMI1	EMI2	Ratio EMI1/EMI2 by educational level			
			Ratio EMI1/EMI2	Low educated	Middle educated*	High educated
1971-1981	15,251	5,953	2.56	2.60	2.60	2.04
1981-1991	21,827	3,090	7.06	7.67	4.68	4.87
1991-2001	31,084	2,227	13.96	17.50	8.36	6.24
2001-2011	38,560	3,164	12.19	15.81	9.55	8.17
2011-2017	N/A	3,102				

Source data: ONS-LS. EMI1: number of emigrants according to the definition 'Did not die during follow up and is not present at next census'; EMI2: number of emigrants according to the definition 'Has an embarkation date during the respective follow up'. * without "other" in 2001-11

Because the EMI1/EMI2 ratios differ by educational level (see Table S3), the bias is likely to be education-specific. That is, especially in the 1991-2001 and the 2001-2011 follow up the ratio between the two emigration definitions is clearly highest for the low educated. This likely reflects a lower likelihood among the low-educated to deregister when they emigrate. Indeed, research mainly attributes underestimation of emigration in population registers (such as the NHSCR) to either a lack of awareness of the requirement to deregister, or a reluctance to do so due to a fear of losing certain benefits (e.g. child support), resident permits or the right to return to the country (Monti et al., 2019), which are arguably more common phenomena amongst individuals in lower socio-economic strata.

The likely substantial underestimation of emigration from 2011 onwards might have impacted trends over time in important outcome measures. Figures S1 illustrates important trend breaks in 2011, and a change in 2011 in the alignment of our ONS-LS data selection with national population and mortality data. First, we observe a large increase in the number of personyears around 2011. Second, we see a trend break in 2011 in both the CDR30+ and the SDR30+. Third, we find – particularly for men – lower SDR for ONS-LS compared to HMD, from 2011 onwards. It should be noted, however, that these trend breaks in 2011 may not only be due to a different emigration definition from 2011 onwards but could also be related to the important increase in the ONS-LS follow-up sample in 2011 compared to the ONS-LS follow-up in 2001 (see next section).

In addition, the relatively small drop in the number of personyears for the low educated in 2011 (see Figure S2) compared to the increase in these numbers for the middle and high educated in the same year is not in line with what could be expected given the educational expansion in E&W (Greenaway & Haynes 2003; Paterson 2022b). This might be – at least partly – related to the underestimation of emigrants (i.e. overestimation of personyears) from 2011 onwards, which – based on the information in Table S3 - might be larger for the low educated.

Information regarding how emigration is dealt with in other studies on educational inequalities in mortality is generally sparse. If emigration is excluded from the different follow-up periods, which is most common, information on how emigration is defined throughout the study period and what this implies for the results is generally not discussed. Because in previous research past trends in socio-economic mortality inequalities have generally used data clubbed in either 5- or 10-year periods, an inconsistency in the data as a consequence of a potential different definition of emigration over time could not easily be identified.

Figure S1. Trends in the number of personyears, the number of deaths, the crude death rate (CDR) and the age-standardised death rate (SDR) comparing our ONS-LS data selection with country-level administrative data from the Human Mortality Database (HMD, 2021), by sex, for individuals aged 30 and older, England and Wales, 1972-2017

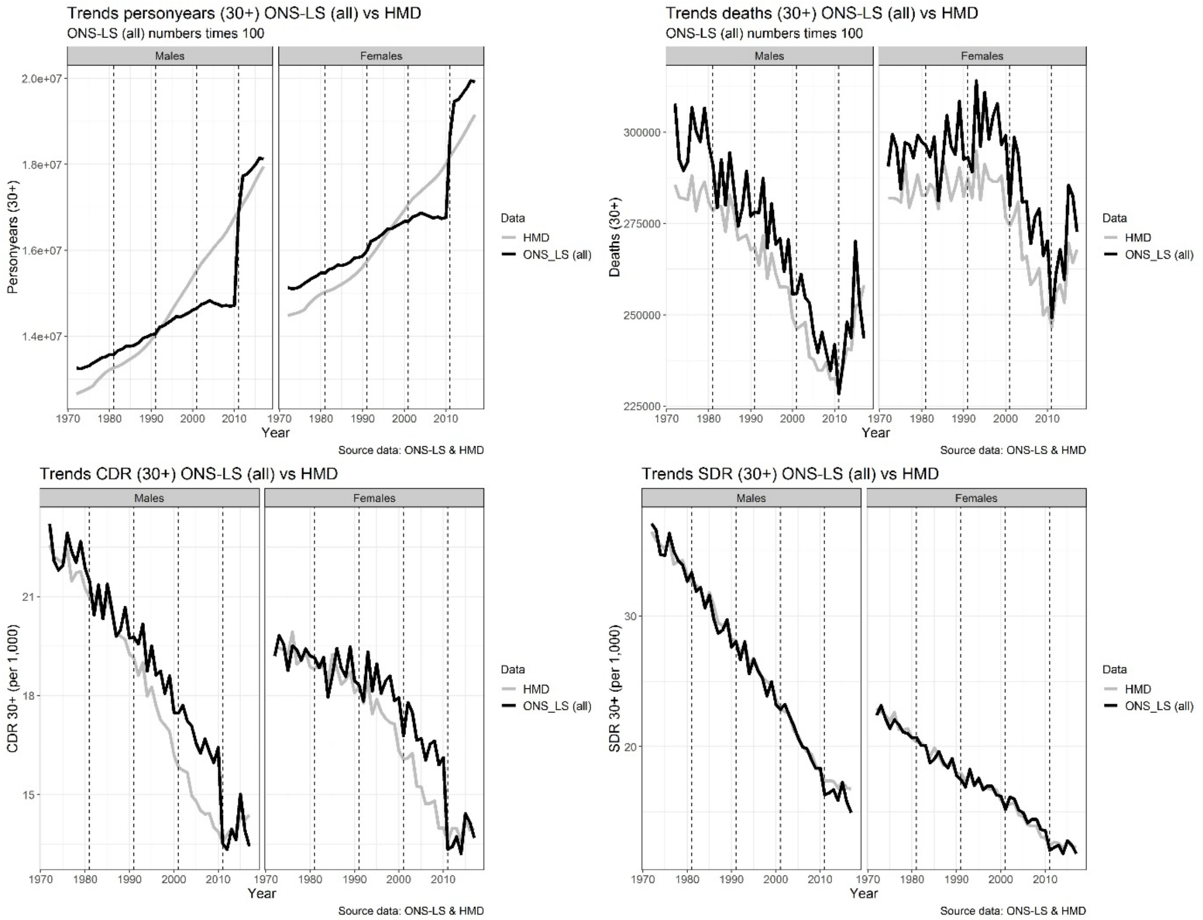

Figure S2. Trends in the number of personyears by educational attainment group after smoothing and redistribution of missings for individuals aged 30 and older, by sex, England and Wales, 2002-2017

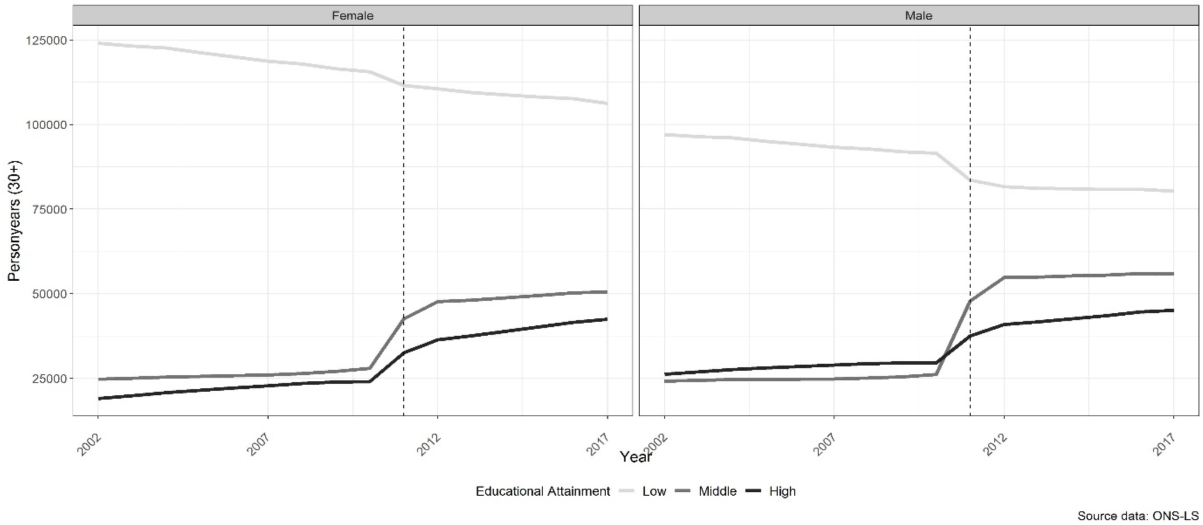

2.5 Imperfect and time-varying alignment with administrative country-level data

Because the increase in the sample size of ONS-LS is not in line with the increase in the general population size, and because of our way of dealing with emigrants and immigrants, the alignment of our ONS-LS data selection with national population and mortality data is not perfect and changes over time. First, there is a change over time in the representativeness of the ONS-LS compared to the general population. Foremost, the ONS-LS 2011 follow-up includes about 43,000 more individuals (all ages) compared to the ONS-LS 2001 follow-up (see Table S4). In contrast, for the earlier years covered by the LS, the number of LS members remained quite stable over time. The relatively large increase in the number of subjects in the ONS-LS 2011 follow-up is attributable to very high response rates to the 2011 Census (94 %) compared to other national censuses (Office for National Statistics, 2012b). Second, our way of dealing with emigration and immigration could have affected the alignment of our ONS-LS selection. That is, both the exclusion of emigrants from the respective follow-up period, and the inclusion of immigrants only from the subsequent follow-up period, likely affected the representativeness of our ONS-LS data selection.

Table S4. Sample size of the ONS-LS at baseline and the subsequent follow-ups, all ages, 30+ and 20+

	Sample size				
	All ages*	30+	30+ excl. emi	20+	20+ excl. emi
1971	524 000	290 739	282 863	364 308	349 057
1981 follow-up	533 000	304 178	291 476	380 304	358 463
1991 follow-up	537 000	319 775	301 417	400 980	369 879
2001 follow-up	538 000	341 807	314 643	406 195	367 605
2011 follow-up	581 000	370 493	368 369	445 549	442 449

* Numbers taken from Table 1 of Shelton et al. 2019. Excl. emi = Excluding emigrants, where emigrants are measured as those not present in the next census, except for the 2011 follow-up where emigrants are measured as those with an embarkation after the 2011 census. Source data: ONS-LS

As a consequence of these two issues, the alignment of our ONS-LS data selection with national population and mortality data is not perfect and changes over time. Figure S1 clearly indicates this. First, for our ONS-LS data selection an important increase in the number of personyears between 2010 and 2012 exists, which results in an important trend break in the CDR and the SDR in 2011. Second, the increase in personyears within our ONS-LS data selection is not in line with the increase in personyears for the national England & Wales population (HMD). That is, between 1971 and 2010, the increase in personyears for our ONS-LS data selection is lower than the increase in personyears for the national England & Wales population, but after the increase in the number of subjects in 2011 in the ONS-LS, the personyears in our ONS-LS data selection returns to being higher than the national population. Both the death numbers (taking into account the 1% sample of the national population that the ONS-LS data comprises) and the trends over time in the number of deaths seem more in line in our ONS-LS data selection with the national population. Consequently, the CDR in our ONS-LS data selection is substantially higher than the CDR for the national population in the period 1990-2010. For women the SDR is also slightly higher in this period in our ONS-LS data selection compared to the national population. For men, however, the SDR in our ONS-LS data selection becomes substantially lower compared to the SDR in the national population in 2011.

3. APPROACH AND METHODS

3.1 Our approach

We implemented a dual data adjustment approach with adjustments at the individual level and at the aggregate level.

At the individual level, we optimised the available education information. That is, to generate a consistent educational classification over time that distinguishes the low, middle and high educated, and aligns well with the ISCED classification, we used information on the educational system in E&W compared to the ISCED classification (e.g. Schneider, 2008), the original census data on education (CALLS-Hub, 2002), and additional information on the educational variables in the ONS-LS (CeLSIUS, 2022a). Compared to previous research that merely used the summary educational variables in the ONS-LS, we used – as well – the more detailed underlying educational variables in the ONS-LS that more directly align with the census questions. Furthermore, we used the general strategy of using educational information from other censuses (e.g. Paterson 2022a), to deal with missing educational information at the individual level.

At the aggregate level, we identified and adjusted for trend discontinuities related to the identified data issues, and for undesirable differences with country-level mortality data for the total population. The adjustment involves the reallocation of people (personyears) in line with information on the data issue at stake, and the consequent reallocation of deaths, by applying the relevant mortality rates. The adjustment, furthermore, includes the use of existing approaches to deal with data-quality related trend discontinuities in cause-specific mortality (Janssen & Kunst, 2004; Van der Stegen et al. 2014). In adjusting our data with administrative country-level mortality data, we maintained the differences between educational groups in the partly-adjusted ONS-LS data.

In absence of quantitative data that can – without problems - be used as external validation beyond the administrative country-level mortality data (see the note at the end of this section), we regard the careful study of trends over time in different outcome measures as an important means of internal validation. Trend discontinuities that can be clearly linked to the identified data issues (from here onwards: "unrealistic trend discontinuities") we regard a sign of invalid data, and should be adjusted. Hence, our choice for the dual data adjustment approach.

More specifically our approach includes the following seven elements.

<u>At the individual level:</u>

a) optimisation of the individual information regarding educational attainment by using, where available and appropriate, the more detailed underlying educational variables in ONS-LS in addition to the main ONS-LS educational variables;
b) the use of educational information from other censuses to deal – as much as possible – with missing educational information.

<u>At the aggregate level:</u>

c) dealing with remaining missings at the aggregate level by proportionally redistributing personyears and deaths with missing educational information to the low, middle and high educated categories, in order to obtain complete period data for those aged 30 and over from 1972 onwards;
d) careful study of trends over time, thereby focussing on the identification of trend discontinuities that can be linked to the identified data issues (from now on we will refer to those as "unrealistic trend discontinuities") and of inconsistencies in the mortality data for the three educational groups combined with administrative country-level mortality data (Human Mortality Database, 2021);

e) adjustment of identified unrealistic trend discontinuities at census years, by using existing approaches to deal with data-quality-related trend breaks in cause-specific mortality resulting from revisions in the International Classification of Diseases (ICD);
f) correcting for the inevitably inconsistent definition of emigration over time by estimating, by sex, the number of "missed"' emigrants (thus, overestimated personyears) in 2011-2017, and applying to them the age- and education-specific distribution of reported emigrations (= "embarkations") in 2011-2017, thereby correcting for educational differences in the reporting of embarkations;
g) adjusting for the imperfect and time-varying alignment of our ONS-LS data selection for the three educational groups combined with administrative country-level population and mortality data from the Human Mortality Database (HMD) by adopting the age-, sex- and year-specific death rates from the latter, while maintaining the differences between educational groups.

Note regarding the external validation of our data:
The formal comparison of our education trends with other quantitative time series data on education is hampered by differences in the sample size, differences in the coverage (age groups, institutionalized or not), differences in the used educational categorisation, differences in the length of the time series covered, and – very importantly – differences in the study of trends (either across cohorts or – as in our case – across calendar years). See as well page 2 of this document. This is the reason, why we pursued the validation of the educational data, foremost, by comparison with similar data from other countries (see appendix III in the supplementary document), and by performing internal validation by examining trends over time in the educational distribution of the population. In addition, we crosschecked our outcomes with information in the available literature on trends in E&W or the UK.

3.2 Methods

All adjustments at the individual level are conducted using STATA. We show the effect of our adjustments at the individual level on the frequencies of ONS-LS members by educational level at the different censuses.

At the aggregate level, we carefully studied trends over time (1972-2017) in different outcome measures, by sex, thereby focussing on the age group 30+, but also exploring the age groups 30-74 and 75+, as well as five-year age groups:
a) the annual share of personyears by educational level (%).
b) the annual share of deaths by educational level (%).
c) the crude death rate (CDR) by educational level and for the ONS-LS population combined.
d) the age-standardised death rate (SDR) by educational level and for the ONS-LS population combined.

To estimate the SDR we applied direct age-standardisation using the 2013 revision of the European Standard Population (European Commission, 2013) as the standard population. This European Standard Population is based on the projected total (=male + female combined) population of the European Union (EU)-27 plus European Free Trade Association (EFTA) countries, based on the Eurostat 2010-based population projections, averaged over the period 2011-2030 (European Commission, 2013). We applied this standard population for those aged 30 and over to the age-specific mortality rates by year, sex, and education group.

In addition, we compared the levels and trends in the SDR, the CDR, the deaths and population numbers for the three educational groups combined based on our ONS-LS data selection, with those based on administrative country-level mortality and population data, for which we used data from the

Human Mortality Database (HMD) (HMD, 2021). We did so, to examine the extent of bias from the inevitably inconsistent definition of emigration (see before), and to obtain additional insights regarding the national representativeness of the overall mortality levels and trends based on the ONS-LS data.

We illustrate the effect of our adjustments at the aggregate level on the trends over time for the different outcome measures listed above.

The overall outcome of our adjustments are the obtainment of time-consistent aggregate mortality data based on the internationally comparable categorization of highest educational attainment into the low, middle, and high educated. Based on these adjusted data, we were able to study the long-term trends in educational inequalities in mortality in E&W by means of established methods. We show the trends over time in both absolute and relative educational inequalities in mortality by means of the slope index of inequality (SII)) and the relative index of inequality (RII)), respectively. Because these inequality measures account for the educational distribution of the population, they are considered well-suited for the comparison of inequalities over time and between countries (Mackenbach & Kunst, 1997; de Gelder et al. 2017). In calculating the RII we applied Poisson regression as per Moreno-Betancur et al. (2015) to the adjusted data by educational level (low, middle, high), which includes the use of a multiplicative Poisson model by year and sex, adjusted for age groups. The SII was calculated from the RII and the SDR in the general population (de Gelder et al. 2017):

$$\mathrm{SII} = \frac{2 * SDR * (RII - 1)}{(RII + 1)}$$

We applied segmented regression to identify potential changes in the trends, using the R package *Segmented*.

4. THE DIFFERENT ELEMENTS OF OUR APPROACH AT THE INDIVIDUAL LEVEL

4.1 Our educational classification

To generate a consistent educational classification over time that distinguishes the low, middle and high educated, and aligns well with the ISCED classification, we used information on the educational system in E&W compared to the ISCED classification (e.g. Schneider, 2008), the original census data on education (CALLS-Hub, 2002), and additional information on the educational variables in the ONS-LS (CeLSIUS, 2022a).

More specifically, we optimised the individual information regarding educational attainment that was available in the ONS-LS by using, where appropriate, the more detailed underlying educational variables in ONS-LS (see Appendix II) in addition to the main ONS-LS education variables (EDUC7, QMLVHIQ8, QMLVHQT9, HLQP0, HLQP11) (see Table S1). We could not use educational information from other – administrative – sources. That is, although administrative data sources generally provide satisfactory school qualification information, in the UK context, this information – owned by the Department for Education - is very restricted in terms of access. Furthermore, currently the individual linkage of these data to the ONS-LS data is not possible.

See Table S5 for our prefinal educational classification and how it is derived based on the available educational information. We refer to this as our pre-final educational classification, because here we still treat those with “other” education as a separate group, whereas subsequently we classified them as middle educated. Table S6 illustrates important differences in the educational distribution of ONS-

LS members aged 20+ at the different censuses, using our pre-final educational variable compared to the main ONS-LS education variables.

First, using the more detailed education variable QMQUAL19 (1st qualification 1991) in the 1991 census, we were able to distinguish among the 352,355 LS-members aged 20 and older with “missing or not applicable” in the main education variable QMLVHQT9, (i) the 350,658 LS-members that did not have any qualifications (QMQUAL19 = -9), and were consequently regarded low educated, and (ii) the 1,697 LS-members with simply had missing education information due to, for example, non-response.

Second, using the more detailed information in the 2001 and 2011 censuses, we were able to identify LS members with professional qualifications (i.e., teaching, nursing, midwifery, health visitors) but no degree, and classified them as middle educated, in line with the classification used in the 1971, 1981 and 1991 censuses. For 2001 this meant using both the QUP0 variable (the 12-digit variable with each digit corresponding to the 12 academic qualification tick boxes used in the qualifications question of the 2001), and the PQUP0 variable (the 6-digit variable with each digit corresponding to the six professional qualification tick boxes used in the professional qualification question in the 2001 Census). For 2011 this meant making use of the QUP11 variable (the 13-digit variable with each digit corresponding to the 13 tick boxes used in the qualifications question in the 2011 Census, including both academic and professional qualifications). For 2001 we reclassified 11,125 LS members aged 20 and older (effectively aged 20-74, because we did not have educational information for individuals aged 75 and older) that had a professional qualification but not a degree, from high to middle educated. In 2011, we reclassified 31,524 LS members aged 20 and older who had a professional qualification but not a degree, from high to middle educated.

Third, by using the more detailed educational information in the 2001 Census, we changed the classification of “other” in 2001 so that it no longer included those with “other professional qualifications”. This ensured that the classification of “other” in 2001 better matched the “other” category in 2011. In doing so, we reclassified 5,900 LS members aged 20 and older (effectively aged 20-74) with other professional qualifications in 2001 to the middle educated.

Finally, to obtain our final education variable, we categorized those with “other” education as “middle” educated. Our main reason for doing so was that the “other” category includes “City and Guilds” qualifications, which are comparable to A-levels (upper secondary degree), but below degree level and therefore can be considered in line with ISCED 3-4. This re-categorization was also made because “other” seems to suggest some form of education, and likely not high education, since obtaining a tertiary education degree is a rather distinct and universal milestone likely to be remembered, also by household members. We, thus, reclassified to the middle educated, 21,356 LS members aged 20 and older (effectively aged 20-74) with other professional qualifications in 2001, and 29,002 LS members aged 20 and older with other professional qualifications in 2011.

Our sensitivity analysis (see Figure S3) revealed that the mortality levels and trends of the ONS-LS members classified as “other” in 2011 and 2021 most closely resemble those of the middle educated without “other” (“middle” in Figure S3), and that merging them with the middle educated (“middle incl. other” in Figure S3) resulted in largely similar mortality levels and trends.

Figure S3. Comparing trends in age-standardised mortality (SDR) for individuals with "other" educational attainment versus the remaining educational attainment groups, for individuals aged 30-74, by sex, England and Wales, 2001-2017*

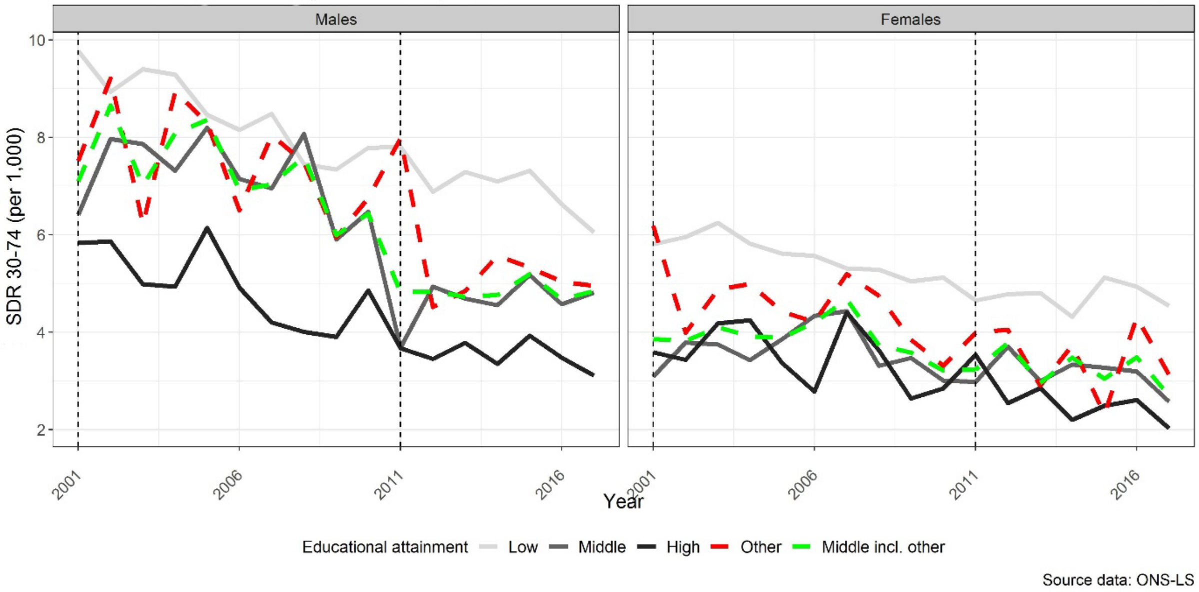

* we focussed on individuals aged 30-74 from 2001 onwards, and not 30+ from 2001 onwards to make sure that the comparison is not affected by our way of dealing with the missing educational information among individuals aged 75 and older in 2001.

In this figure "Middle" refers to the middle educated without the "other" educated, in line with our prefinal classification of highest educational attainment.

Table S5. Our prefinal classification of highest educational attainment based on the ONS-LS education variables (= our education variable that still separately distinguishes "other", which subsequently we merged with "middle")

Census	Education*	Education variable(s)**	Highest educational attainment
**1971**	High	EDUC7=0 | EDUC7=1	Higher university degrees; Other degrees, and equivalents
	Middle	EDUC7=2 | EDUC7=3	A-level and equivalent; Other qualifications higher than A-level but below degree level
	Low	EDUC7=4	None
	Missing	EDUC7=5	None stated
**1981**	High	QMLVHIQ8=1 | QMLVHIQ8=2	Higher degrees of UK standard; First degrees and all other qualifications of first degree standard
	Middle	QMLVHIQ8=3	Qualifications above GCE O- and A-levels but not first and higher degrees
	Low	QMLVHIQ8=-9	No qualifications obtained since the age of 18
	Missing	QMLVHIQ8=4	Highest qualification is not stated
**1991**	High	QMLVHQT9=1 | QMLVHQT9=2	Higher degrees of UK standard; First degrees and all other qualifications of first-degree standard
	Middle	QMLVHQT9=3	Qualifications above GCE O- and A-levels but not first and higher degrees
	Low	QMQUAL19=-9	No qualifications obtained since the age of 18 excluding qualifications normally obtained at school
	Missing	Remaining QMLVHQT9=-9	Rest of "not applicable or missing"
**2001**	High	QUP0_5=1 | QUP0_6=1 | QUPO_10 =1	First Degree (e.g., BA, BSc); Higher Degree (e.g., MA, PhD, PGCE, post-graduate certificates/diplomas); NVQ Level 4-5, HNC, HND
	Middle	HLQP0=13 | qualprofdegree=0 | otherprofother=1. We make use of QUP0 and PQUP0.	Upper-level secondary qualifications, such as A-levels and advanced GNVQ; Professional qualifications (qualified teachers, medical doctors, dentists, nurses) without a degree; Other professional (= non-academic) qualifications.
	Low	HLQP0=10 | HLQP0=11 | HLQP0=12	No academic or professional qualifications; Qualifications below upper-level secondary education, such as CSEs and GCSEs
	Other	Remaining HLQP0=15	Other academic qualifications (e.g., City and Guilds, RSA/OCR, BTEC/Edexcel)
	Missing	HLQP0=-7 | HLQP0=-9	Missing; Not applicable
**2011**	High	QUP11_8=1 | QUP11_9=1	Degree (for example, BA, BSc) and higher degree (for example, MA, PhD, PGCE); NVQ Level 4-5, HNC, HND, RSA Higher Diploma, BTEC Higher Level
	Middle	HLQP11=13 | HLQP11=14 | (QUP11_10=1 & QUP11_8=0 & QUP11_9=0)	Apprenticeships; Upper-level secondary qualifications, such as A-levels, advanced GNVQ, advanced Baccalaureate; Professional qualifications without a degree
	Low	HLQP11=10 | HLQP11=11 | HLQP11=12	No qualifications; Qualifications below upper-level secondary educations, such as CSEs and GCSEs
	Other	HLQP11=16	Other: Vocational/Work-related Qualifications, Foreign Qualifications/ Qualifications gained outside the UK (NI) (Not stated/level unknown)
	Missing	HLQP11=-6 | HLQP11=-9	Missing; No code required

Source information: ONS-LS
* Low = no, pre-primary, primary, and lower secondary education (ISCED-1997 0-2); middle = upper secondary and post-secondary non-tertiary education (ISCED-1997 3-4); high = tertiary education (ISCED-1997 5-6) (UNESCO, 1997)** Table S1 lists the summary ONS-LS educational variables; Appendix II lists the additional educational variables used.

Table S6. Distribution of the population by educational attainment for our prefinal educational variable (= our education variable that still separately distinguishes "other", which subsequently we merged with "middle") compared to the main educational variables in ONS-LS and their relation*, for ONS-LS members aged 20+ at the different decennial censuses

Main educational variables in ONS-LS		N	*	Our educational variable		N
1971 (EDUC7)				1971		
0	Higher university degree	944	\	3	High (ISCED 5-6)	11,528
1	Other degrees and equivalents	10,584	/			
2	Other qualifications > than A level	15,019	\	2	Middle (ISCED 3-4)	31,911
3	A level and equivalents	16,892	/			
4	None	300,171	=	1	Low (ISCED 0-2)	300,171
5	None stated	25,324	=	0	Missing	25,324
		368,934				368,934
1981 (QMLVHIQ8)				1981		
1	Highest qualification is level 'a'	2,148	\	3	High (ISCED 5-6)	21,335
2	Highest qualification is level 'b'	19,187	/			
3	Highest qualification is level 'c'	17,730	=	2	Middle (ISCED 3-4)	17,730
4	Highest qualification not stated	41	=	0	Missing	41
-9	No qualifications	344,003	=	1	Low (ISCED 0-2)	344,003
		383,109				383,109
1991 (QMLVHQT9)				1991		
1	Highest qualification is level 'a'	3,852	\	3	High (ISCED 5-6)	29,041
2	Highest qualification is level 'b'	25,162	/			
3	Highest qualification is level 'c'	24,903	=	2	Middle (ISCED 3-4)	24,903
-9	Not applicable or missing	352,355	/	1	Low (ISCED 0-2)	350,658
			\	0	Missing	1,697
		406,272				406,272
2001 (HLQP0)				2001		
10	No academic / professional qualifications	111,511	=	1		
11	Level 1	60,394	=	1		
12	Level 2	63,850	=	1	Low (ISCED 0-2)	235,755
13	Level 3	27,259	≠	2	Middle (ISCED 3-4)	44,284
14	Level 4/5	74,826	≠	3	High (ISCED 5-6)	63,701
15	Other qualifications/ level unknown**	27,256	≠	5	Other	21,356
-7	Missing	21	=	0		
-9	Not applicable	43,098	=	0	Missing	43,119
		408,215				408,215
2011 (HLQP11)				2011		
10	No academic / professional qualifications	106,321	=	1		
11	Level 1	56,878	=	1		
12	Level 2	60,440	=	1	Low (ISCED 0-2)	223,639
13	Apprenticeship	16,441	≠	2	Middle (ISCED 3-4)	99,236
14	Level 3	51,271				
15	Level 4+ and professional qualifications	128,648	≠	3	High (ISCED 5-6)	97,124
16	Other qualifications/level unknown***	29,002	=	5	Other	29,002
-6	Missing	97	=	0		
-9	No code required	1,151	=	0	Missing	1,248
		450,249				450,249

Source data: ONS-LS.

* The 4th column shows the relation between the different categories of our educational variable compared to the ONS-LS main educational variables. '=>' indicates that the ONS-LS category is included in our category, but that our category includes more than that. '<=' indicates that our category includes part of the ONS-LS category, and that part of the ONS-LS category has gone to another category; ** Other qualifications (eg City and Guilds etc), Other Professional qualifications; *** Vocational/Work-related Qualifications, Foreign Qualifications/ Qualifications gained outside the UK (NI) (Not stated/level unknown) (England & Wales & Northern Ireland)

4.2 Dealing with missing educational information (individual level)

We dealt with the missing educational information – which was particularly large among the elderly in the 1971 and 2001 censuses (who were not required to answer the education questions), and among the non-elderly in the 1971 census - as much as possible at the individual level. In doing so, we used educational information from other censuses, which is a common strategy of those performing longitudinal studies regarding education using the ONS-LS or its sister-studies (e.g. Iveson et al. 2020; Paterson 2022a). In using educational information from other censuses, we decided to purely rely on information from the previous census. This, because we considered the most recent information the most reliable. In addition, we deliberately refrained from using educational information from the next census, because for those who died educational information from the next census would not be available and would introduce bias.

We also decided, in the end, to purely use the educational information from the previous census for individuals aged 75 and older in the 2001 census. Firstly, they constitute a distinct group whom were not asked about their educational level at all and for whom we consequently had no educational information based on the 2001 Census. Secondly, for individuals aged 70 and older with missing educational information in the 1971 Census, our decision to purely rely on information from the previous census, implied that we could not adequately deal with the missing educational information at the individual level. However, different from the 2001 Census, 67 % of the respondents aged 70 and older did respond to the education question (see 2.3: only those 70+ without a job in the last week did not need to answer). This provided at least some information on the educational distribution for this age group, which we used to address their missing educational information at the aggregate level (see section 5.2). Thirdly, we refrained from using the educational information from the previous census as well for the non-elderly with missing educational information, because this applied predominantly to the non-elderly in the 1971 Census, for whom we did not have information from a previous census. But also, because especially for younger ages using educational information from an earlier census is not ideal because their educational attainment may still change over time. Also, for this group, we consequently decided to address the missing educational information at the aggregate level.

Of the 41,960 ONS-LS members aged 75 and older in 2001, who all had missing educational information, 38,650 (92.1 %) were present in the 1991 Census. For them, we could, therefore, use the educational information available from the 1991 Census. Of these 38,650 ONS-LS members, 35,564 (92%) could be assigned as low educated, 1,557 (4%) as middle educated, 1,297 (3%) as high educated, and 232 (0.6%) had missing educational information as well in 1991 (Table S7).

See Table S8 for the final frequencies after performing the adjustments at the individual level.

Table S7. Using the educational information from the 1991 census for the elderly ONS-LS members in the 2001 census, which all had missing educational information

ONS-LS members	Number (frequency)
Aged 75+ in the 2001 census (with missing educational information)	41,960
Present in the 1991 census	38,650 (92.1 %)
Of individuals present in the 1991 census (N = 38,650):	
Low educated in 1991	35,564 (92.0 %)
Middle educated in 1991	1,557 (4.0 %)
High educated in 1991	1,297 (3.4 %)
No educational information in 1991	232 (0.6 %)

Source data: ONS-LS.

Table S8. Frequencies of ONS-LS members by highest educational attainment for different age groups at the different censuses, based on our final educational variable, and after dealing with missing educational information at the individual level

	Missing	Low	Middle	High	Total
1971					
% aged 30 and older	7.88 (23,170)	82.52 (242,651)	6.82 (20,042)	2.79 (8,195)	294,058
% aged 20 and older	6.86 (25,324)	81.36 (300,171)	8.65 (31,911)	3.12 (11,528)	368,934
% aged 30-69	3.59 (9,002)	85.49 (214,261)	7.78 (19,506)	3.13 (7,851)	250,620
% aged 20-69	3.43 (11,156)	83.50 (271,781)	9.64 (31,375)	3.44 (11,184)	325,496
% aged 70 and older	32.62 (14,168)	65.36 (28,390)	1.23 (536)	0.79 (344)	43,438
1981					
% aged 30 and older	0.01 (36)	90.43 (276,710)	4.49 (13,738)	5.07 (15,522)	306,006
% aged 20 and older	0.01 (41)	89.79 (344,003)	4.63 (17,730)	5.57 (21,335)	383,109
% aged 30-69	0.01 (23)	89.25 (226,052)	5.03 (12,745)	5.71 (14,450)	253,270
% aged 20-69	0.01 (28)	88.79 (293,345)	5.07 (16,737)	6.13 (20,263)	330,373
% aged 70 and older	0.02 (13)	96.06 (50,658)	1.88 (993)	2.03 (1,072)	52,736
1991					
% aged 30 and older	0.30 (966)	86.60 (280,040)	6.30 (20,361)	6.81 (22,021)	323,379
% aged 20 and older	0.42 (1,697)	86.31 (350,658)	6.13 (24,903)	7.14 (29,014)	406,272
% aged 30-74	0.31 (896)	85.46 (243,632)	6.8 (19,392)	7.42 (21,162)	285,082
% aged 20-74	0.44 (1,627)	85.40 (314,250)	6.50 (23,934)	7.65 (28,164)	367,975
% aged 75 and older	0.18 (70)	95.07 (36,408)	2.53 (969)	2.22 (850)	38,297
2001					
% aged 30 and older	12.27 (42,063)	58.78 (201,567)	15.02 (51,500)	13.93 (47,777)	342,907
% aged 20 and older	10.56 (43,119)	57.75 (235,755)	16.08 (65,640)	15.60 (63,701)	408,215
% aged 30-74	0.03 (103)	66.98 (201,567)	17.11 (51,500)	15.88 (47,777)	300,947
% aged 20-74	0.32 (1,159)	64.37 (235,755)	17.92 (65,640)	17.39 (63,701)	366,255
% aged 75 and older	100 (41,960)	0.00 (0)	0.00 (0)	0.00 (0)	41,960
2001 – using for 75+ the educational attainment from the 1991 census					
% aged 30 and older	1.06 (3,645)	69.15 (237,131)	15.47 (53,057)	14.31 (49,074)	342,907
% aged 20 and older	1.15 (4,701)	66.46 (271,319)	16.46 (67,197)	15.92 (64,998)	408,215
% aged 30-74	0.03 (103)	66.98 (201,567)	17.11 (51,500)	15.88 (47,777)	300,947
% aged 20-74	0.32 (1,159)	64.37 (235,755)	17.92 (65,640)	17.39 (63,701)	366,255
% aged 75 and older	8.44 (3,542)	84.76 (35,564)	3.71 (1,557)	3.09 (1,297)	41,960
2011					
% aged 30 and older	0.04 (135)	52.14 (194,335)	27.49 (102,472)	20.34 (75,797)	372,739
% aged 20 and older	0.28 (1,248)	49.67 (223,639)	28.48 (128,238)	21.57 (97,124)	450,249
% aged 30-74	0.04 (115)	49.39 (161,107)	28.27 (92,202)	22.31 (72,761)	326,185
% aged 20-74	0.30 (1,228)	47.17 (190,411)	29.22 (117,968)	23.31 (94,088)	403,695
% aged 75 and older	0.04 (20)	71.38 (33,228)	22.06 (10,270)	6.52 (3,036)	46,554

Source data: ONS-LS

5. THE DIFFERENT ELEMENTS OF OUR APPROACH AT THE AGGREGATE LEVEL

5.1 Initial steps

5.1.1 From individual-level cohort data to aggregate period data

We rearranged the individual data of ONS-LS members aged 20 and older for the five different follow-ups (census 1971 to census 1981; census 1981 to census 1991; census 1991 to census 2001; census 2001 to census 2011; census 2011 to December 31 2017) into aggregate period data by single year of age and single calendar year (1972 up to 2017). Based on cohort data for those aged 20 and older and 10-year follow-ups, we were able to obtain complete age-period data for those aged 30 and over.

We first estimated the person days at risk for individuals at the start of the five 10-year follow up cohorts, using the Stata *stset* command, whereby we count the days from the census date to the exact date of dying (constructed from day, month and year of death), or the end date of the follow up, whichever comes first. For the last follow-up period we added the date of embarkation (i.e. our operationalisation of emigration for that period) as an additional end point of observation.

We then rearranged the individual level data into person-period records for each age and calendar year an individual is present in the data (using the *stsplit* command in Stata). To determine the age at onset of each calendar year, we made use of the exact date of birth.

Subsequently, we summed these records into aggregated data by single year of age and single calendar year. To end up with full calendar years and because the 1971 Census was administered in April, we start the official observation from January 1st, 1972. Data in subsequent census years (1981, 1991, 2001, 2011) were constructed using both the information from the preceding follow-up period and the subsequent follow-up period.

Table S9 shows the shares of the deaths and personyears by highest educational attainment group (low, middle, high) for individuals aged 30 and older by sex and for different periods. Largely the same observations can be made compared to those based on Table S8, which was based on cohort data.

Table S9. Share of personyears and deaths by educational attainment group (%) over 10-year periods (weighted averages), for individuals aged 30 and older, by sex, England and Wales, 1972-2017

Period*	Missing	Low	Middle	High
	**Share of personyears by highest educational attainment (%)**			
Total				
1972-1981	5.28	83.73	7.72	3.27
1982-1991	0.03	89.14	5.04	5.78
1992-2001	0.39	84.45	7.29	7.87
2002-2011	0.58	65.98	17.13	16.31
2012-2017	0.04	50.29	27.75	21.92
Males				
1972-1981	4.13	82.18	8.31	5.38
1982-1991	0.04	86.64	4.97	8.36
1992-2001	0.45	81.65	7.11	10.80
2002-2011	0.45	61.94	18.14	19.47
2012-2017	0.05	45.10	30.86	23.99
Females				
1972-1981	6.28	85.10	7.20	1.42
1982-1991	0.02	91.35	5.11	3.52
1992-2001	0.35	86.91	7.44	5.30
2002-2011	0.69	69.55	16.24	13.52
2012-2017	0.04	55.01	24.92	20.04
	**Share of deaths by highest educational attainment (%)**			
Total				
1972-1981	16.98	78.87	2.83	1.33
1982-1991	0.05	95.71	2.04	2.20
1992-2001	0.59	93.43	3.24	2.74
2002-2011	3.89	81.03	9.88	5.20
2012-2017	0.04	70.59	22.26	7.10
Males				
1972-1981	13.17	81.42	3.19	2.22
1982-1991	0.07	94.47	1.98	3.49
1992-2001	0.59	91.44	3.37	4.60
2002-2011	3.05	76.47	12.42	8.07
2012-2017	0.03	61.46	28.41	10.09
Females				
1972-1981	20.82	76.29	2.46	0.42
1982-1991	0.03	96.9	2.10	0.98
1992-2001	0.58	95.22	3.13	1.06
2002-2011	4.64	85.06	7.64	2.66
2012-2017	0.05	78.97	16.62	4.36

Source data: ONS-LS.

5.1.2 Smoothing

Before adjusting the data at the aggregate level, we smoothed the sex-specific deaths and personyears for the high, middle and low educated. This was necessary because very low death numbers occurred among high educated women in the early years in particular, but also for the middle educated in selected years in the 1980s, and quite a few strata existed with zero deaths (Table S10).

Table S10. Number of strata with zero counts of deaths or personyears by educational attainment group by ten-year periods, for individuals aged 30 and older, by sex, England and Wales, 1972-2017

	Deaths			Personyears		
Period	Low	Middle	High	Low	Middle	High
Males						
1972-1981	0	23	22	0	6	2
1982-1991	0	34	13	0	2	0
1992-2001	0	20	11	0	1	0
2002-2011	0	2	3	0	0	0
2012-2017	0	1	0	0	0	0
Females						
1972-1981	0	18	66	0	1	4
1982-1991	0	22	34	0	0	2
1992-2001	0	15	22	0	0	0
2002-2011	0	4	8	0	0	0
2012-2017	0	2	2	0	0	0

Source data: ONS-LS.

Consequently, performing any adjustment at the age-specific level proved very dependent on these small and non-robust numbers. More generally, zeros are rather difficult to work with when performing adjustments. For example, for the proportional redistribution of missings, it would lead to no extra deaths/personyears for strata with respectively no death numbers or no personyears. In addition, the smoothing could prevent huge and unrealistic fluctuations in SDR by educational level over time.

While applying the smoothing we made sure that the total counts for the different five-year age groups combined (by year, sex and educational level) and for the different educational attainment groups combined (by year, sex, and five-year age group) were maintained. This yields equal CDRs by educational level, and equal SDRs for the different educational groups combined, before and after applying the smoothing.

As our main smoothing method we selected the one-dimensional Rizzi et al. (2015) smoothing technique, which essentially is a technique to smooth age distributions of counts. The Rizzi et al. (2015) smoothing technique is based on a composite link model, added with a penalty to ensure the smoothness of the underlying distribution (see Rizzi et al. 2015 for more information). Optimization is done by maximizing a penalized likelihood. The method is not only robust to outliers but also ensures that the total counts are maintained. We applied the method separately to both the death numbers and the personyears by year, sex and educational level (low, middle, high) using the R package *ungroup* (Pascariu et al., 2018). In doing so, we selected 100 as the upper age limit of the open-ended age group 95+. To make sure that the totals for the three educational groups combined (by year, sex, and five-year age group) are also maintained, we proportionally redistributed – separately for the deaths and the personyears – the differences between the original and the smoothed counts for the three educational groups combined, to the three educational groups, according to the education-

specific smoothed proportions. Subsequently, we applied the age distributions based on these obtained smoothed numbers to the original total for the different ages combined.

For the personyears the smoothing procedure resulted in smooth age patterns that were largely in line with the original age patterns (Figure S4). In fact, the smoothing only has a small visible effect for the personyears among high educated women in 1972-1983. For the deaths, however, particularly for high educated women (1991 up to 2001) and also for the middle educated in selected years, the Rizzi smoothing did not result in a realistic wave-shaped age pattern of deaths (Figure S5), simply because numbers are too few to start with. To obtain more robust outcomes for the deaths, still maintaining the totals, we instead, applied, for each year by sex, smoothed education-specific age patterns of dying to the education-specific deaths aged 30 and older. The smoothed age patterns by year, sex and educational level were obtained by dividing Rizzi smoothed five-year moving average death counts by the observed death counts for 30+. We, subsequently, made sure that the totals for the three educational groups combined (by year, sex, and five-year age group) were also maintained. After applying this slightly more advanced smoothing procedure, we obtained more realistic age patterns of dying for both the middle and the high educated (Figure S6). For the low educated, the smoothing hardly had an effect.

Before implementing the final smoothing approach, we checked the resulting CDR and SDR levels. It showed that for middle educated men in 2010 an unrealistic peak in the SDR resulted with levels higher than the SDR for the low educated, due to much higher smoothed deaths after age 75 compared to the baseline deaths. We decided, therefore, to use the non-smoothed baseline death numbers instead of the smoothed death numbers for middle educated men in 2010. We implemented this in such a way that the totals for the different age groups combined and the different educational groups combined are maintained.

Because we kept the totals for the different age groups combined and for the different educational groups combined constant, the trend in CDR by educational group has remained the same, as well as the trend in the share of deaths by educational level for 30+, and the trend in the share of personyears by educational level for 30+. The age-specific mortality rates for the low educated remain largely the same, and for the middle and high educated they are more in line with the expected exponential increase in mortality rates after age 30 (Gompertz, 1825) (Figure S7). The small change in age-specific mortality rates for the middle and high educated results in a small effect on the SDR for these groups (Figure S8).

Our smoothing approach hence does not resolve the issue of fluctuations, and – likewise – it does not resolve the issue of the unstable estimates of SDR for the high and middle educated in particular. This was a deliberate choice, as we did not want the smoothing approach to be too invasive. In line with our intentions, our approach solves the issue of zeros, which would hamper subsequent data adjustments, and it enables the use of a more realistic age pattern of deaths in our data adjustment.

Figure S4. Relative age pattern of personyears before and after applying the smoothing procedure, by sex and educational group, England and Wales, selected years. *Age groups: 30 = 30-34, … , 90 = 90-94, 95 = 95+.*

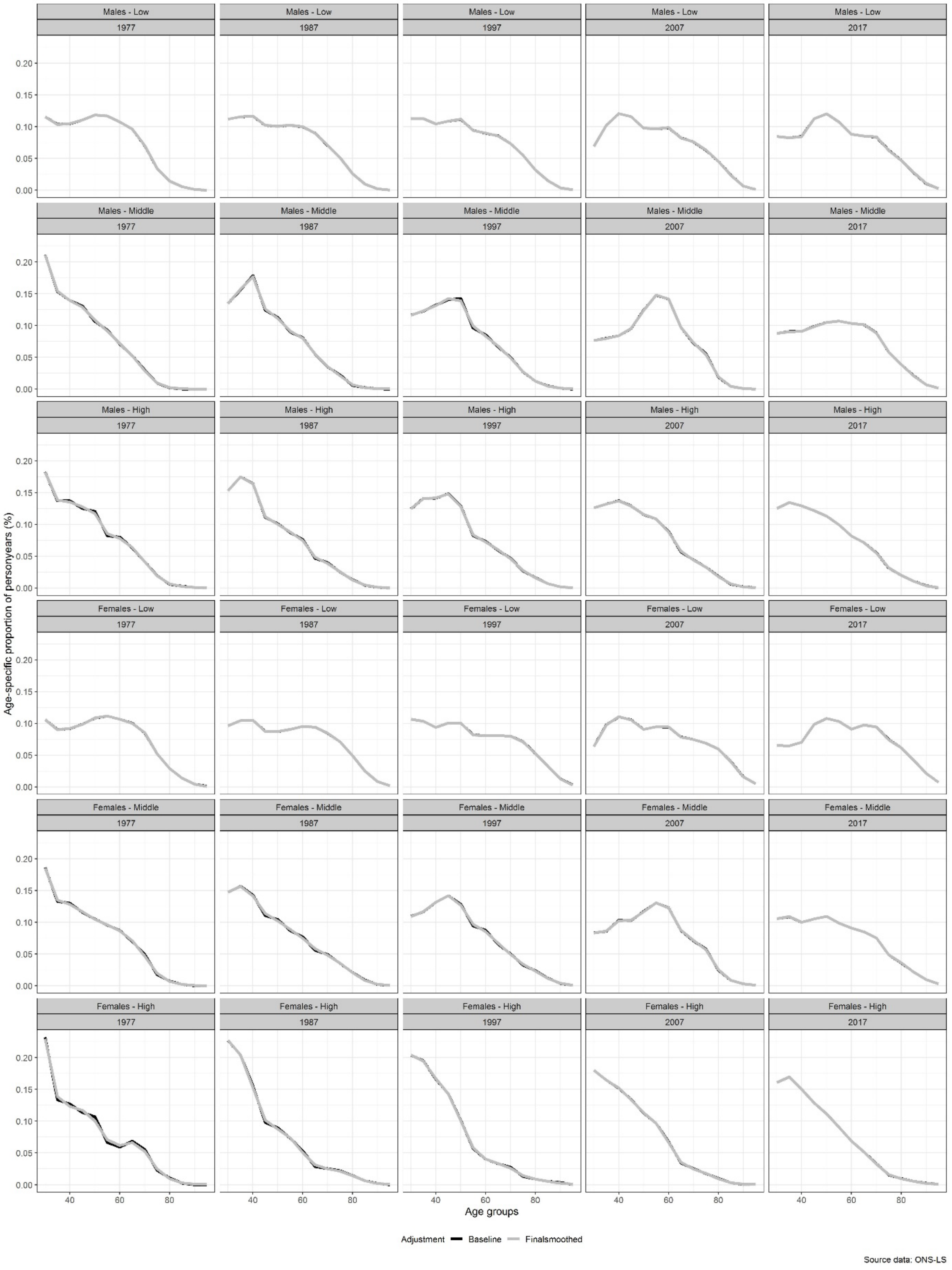

Figure S5. Relative age pattern of deaths before and after applying the Rizzi et al. 2015 smoothing procedure, by sex and educational group, England and Wales, selected populations. *Age groups: 30 = 30-34, … , 90 = 90-94, 95 = 95+.*

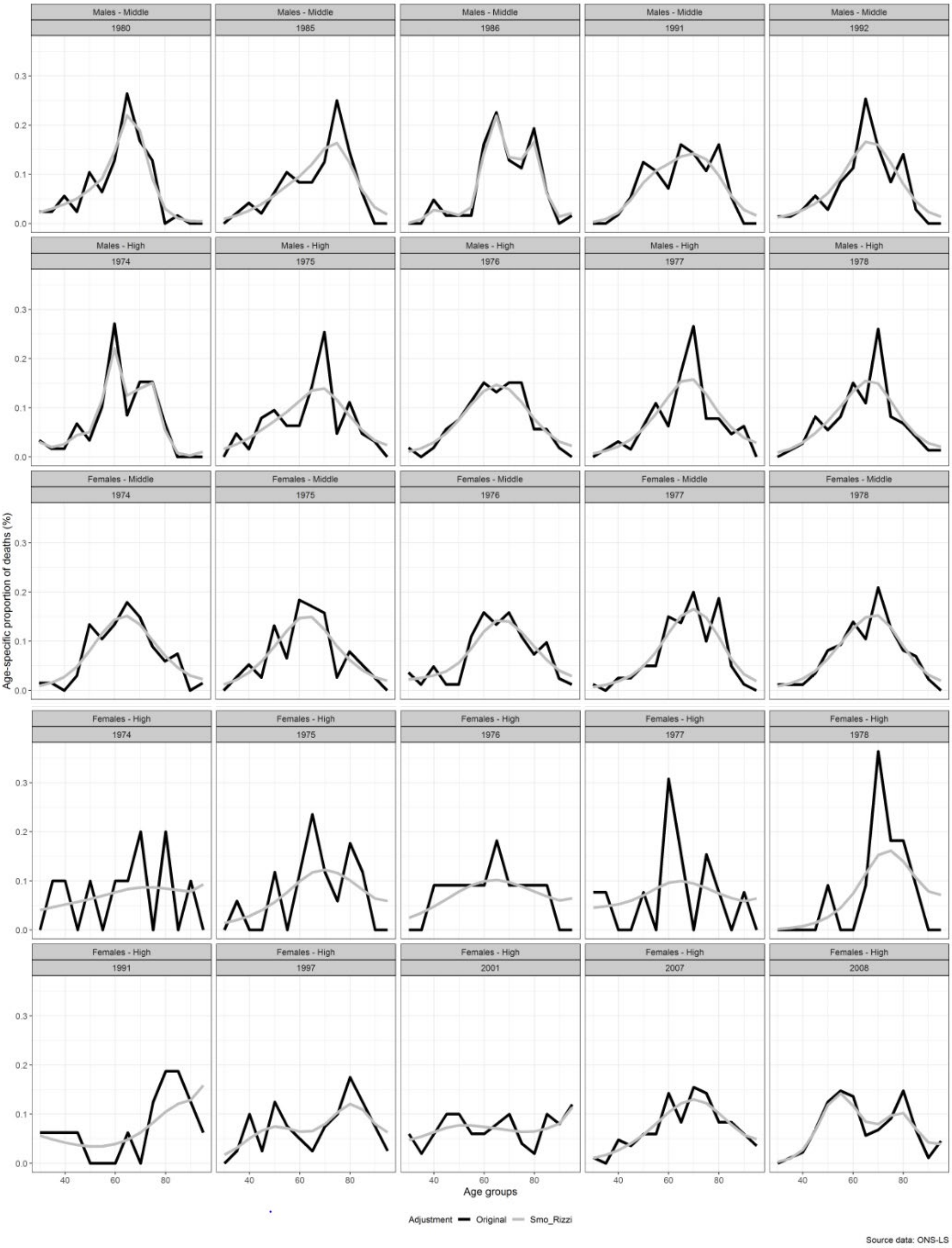

Figure S6. Relative age pattern of deaths before and after applying the smoothing procedure, by sex and educational group, England and Wales, selected years. *Age groups: 30 = 30-34, … , 90 = 90-94, 95 = 95+.*

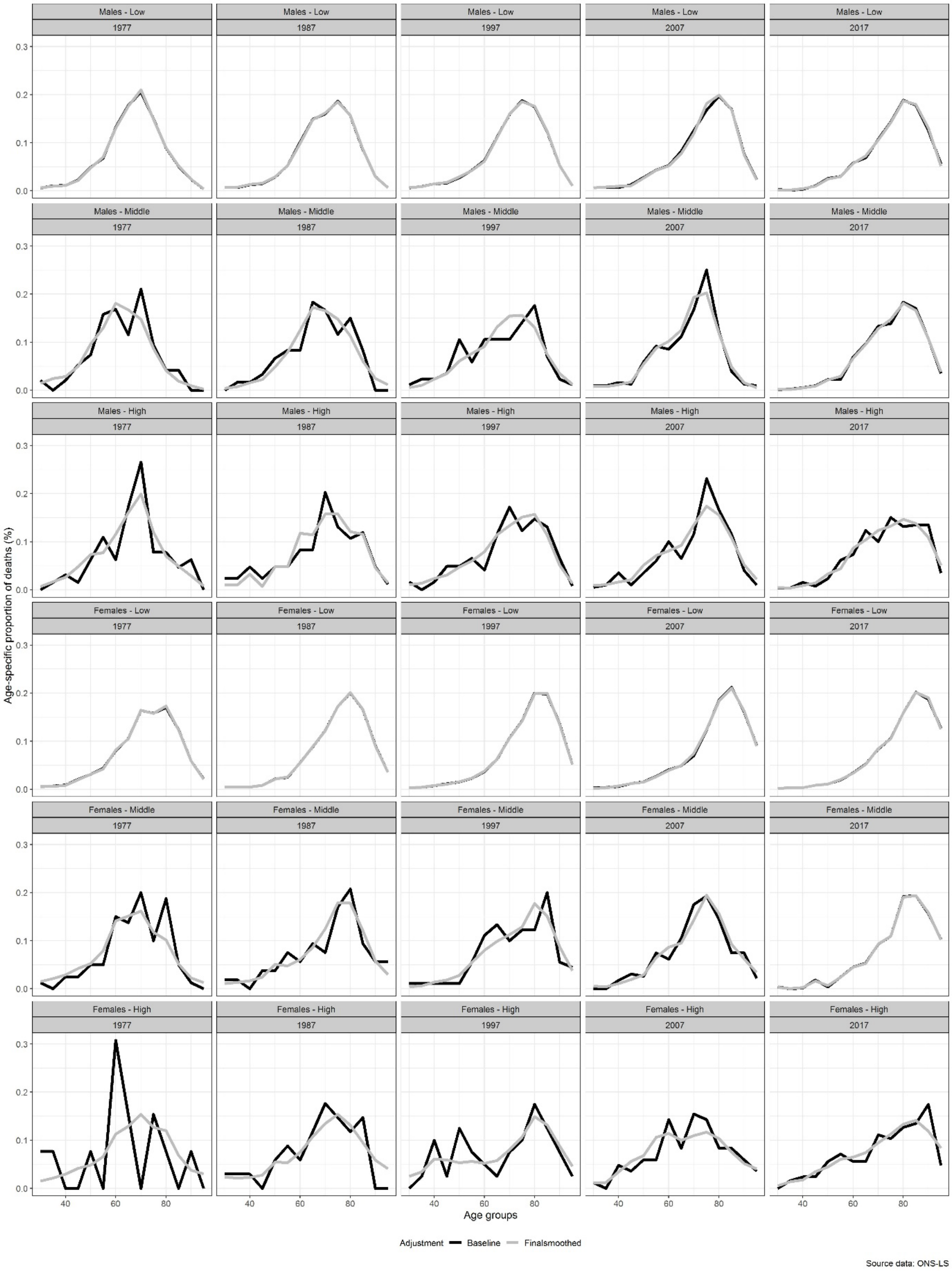

Figure S7. Age-specific mortality rates (log scale) before and after applying the smoothing procedure, by sex and educational group, England and Wales, selected years. *Age groups: 30 = 30-34, … , 90 = 90-94, 95 = 95+.*

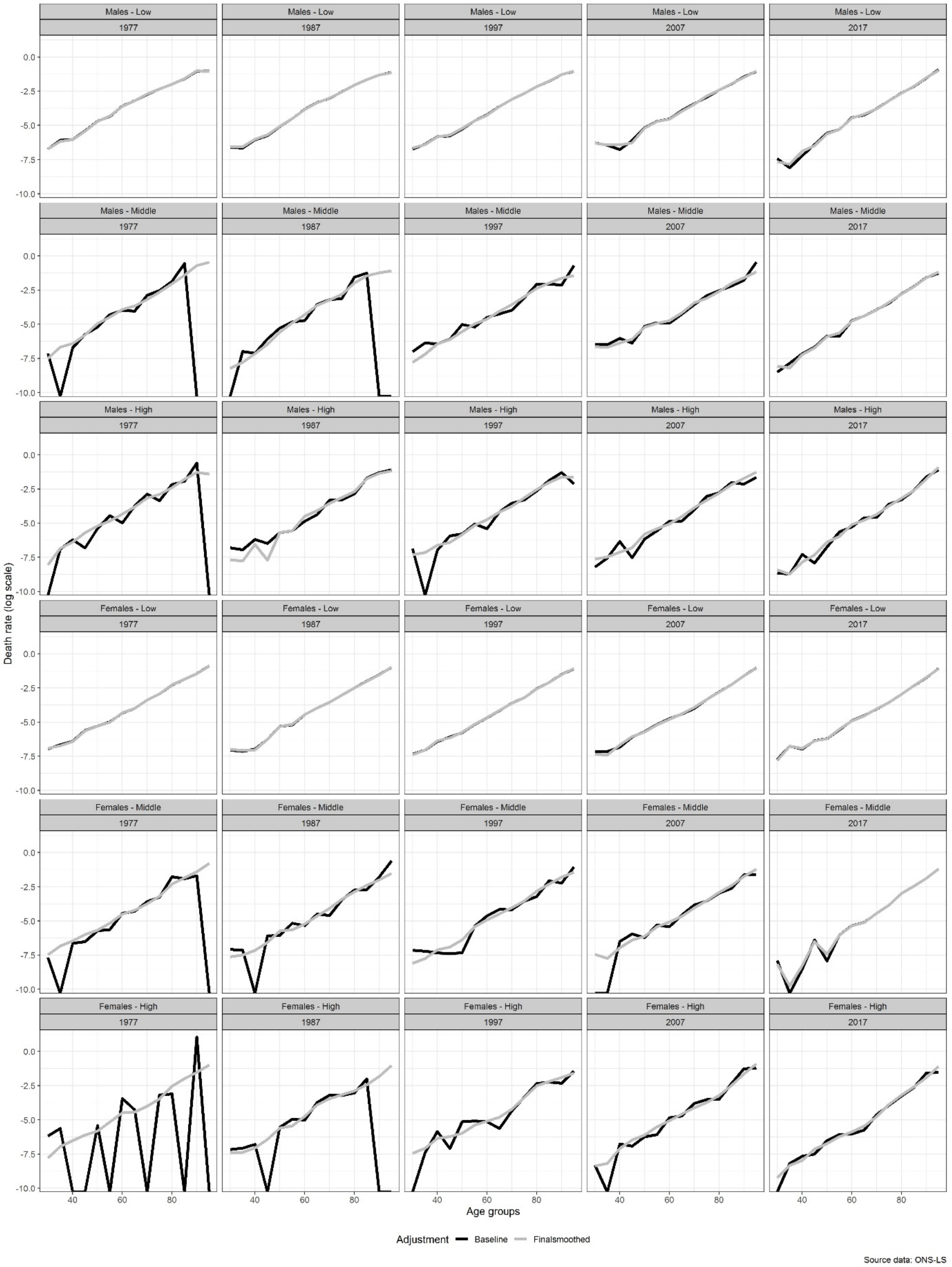

Figure S8. Trends in the age-standardised death rate (SDR) by educational attainment group before and after smoothing, for broad age groups (30+, 30-74, 75+), by sex, England and Wales, 1972-2017

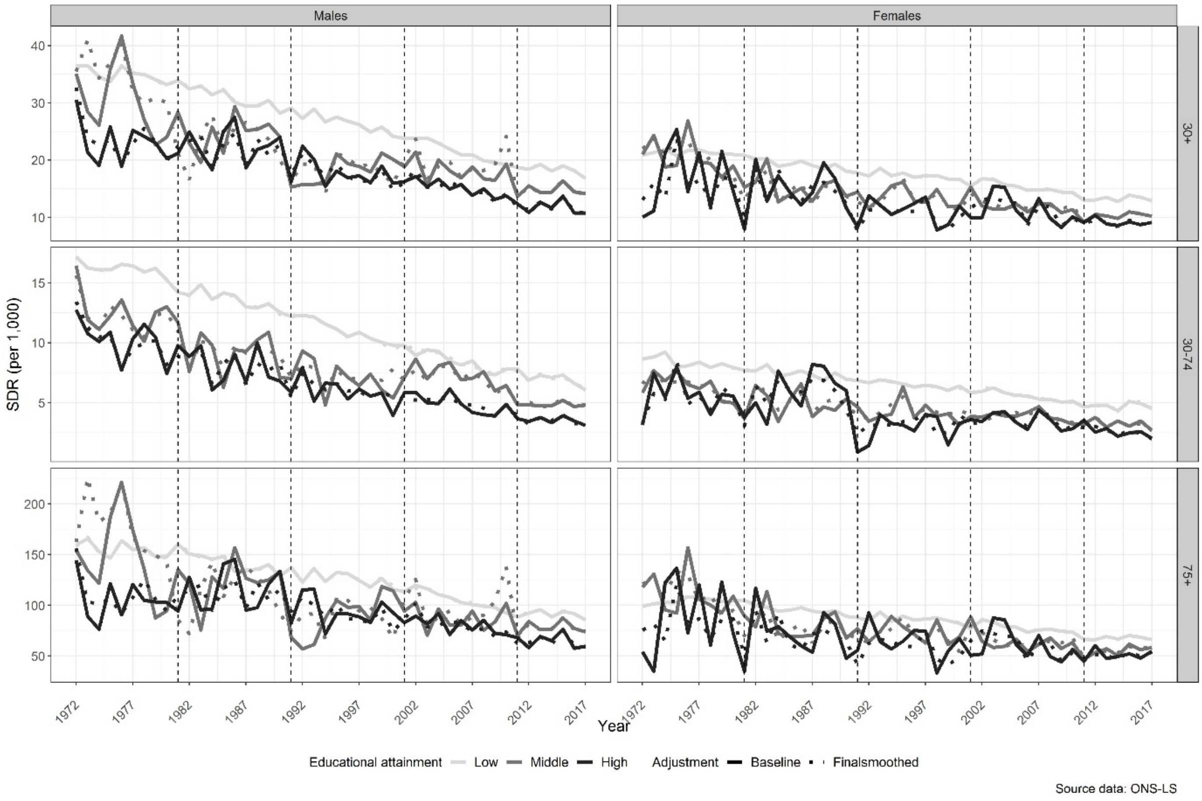

5.2 Proportional redistribution of missings

Because we could not deal adequately with the entirety of missing educational information at the individual level, we dealt with the remaining instances of missing educational information (Table S7) at the aggregate level. At the aggregate level, the (remaining) people with missing educational level contributed – overall – 183,771 personyears, and 12,472 deaths, which amounts to, respectively 1.3% and 4.8% of the total personyears (14,372,539) and total deaths 257,413 (Table S9). Missing educational information particularly exists among individuals aged 30 and older in 1972-1981 and aged 75 and older in 2001-2011.

We proportionally redistributed these deaths and personyears with missing educational information, to the low, middle and high educated categories according to the smoothed relative share of the respective educational category by year, sex, and age. In doing so, we assumed that those with missing educational information are missing at random. In other words, those for whom information regarding education is available are assumed to be representative of the educational distribution of the entire population. The same assumption is essentially applied when one simply omits those with missing educational information. The advantage of our approach – over omitting the missings - is that we maximise the power of the available data and that the sum of the personyears and deaths over the different educational levels is then more likely to be consistent with the numbers at the national level (taking into account the sample size) and to be consistent over time. We applied the proportional redistribution to the smoothed data (low, middle, high), to make sure that strata with zero death counts in the original data would also obtain a small percentage of the missings.

Of the personyears with missing educational level 167,712 (91.3%) were assigned low educated, 10,191 (5.5%) middle educated, and 5,868 (3.2%) high educated (Table S11). Of the deaths with missing educational level, 11,948 (95.8%) were assigned low educated, 333 (2.7%) middle educated,

and 191 (1.5%) high educated. For individuals aged 30-69 in 1972-1981 relatively fewer personyears and deaths with missing educational level were assigned to low (88.0% and 93.0%, respectively) compared to overall, and consequently more personyears and deaths were assigned to the middle and high educated. For individuals aged 70 and older in 1972-1981 the personyears and deaths were assigned relatively more to the low educated compared to overall (96.2% and 97.0%, respectively). For individuals aged 75 and older in 2002-2011 again relatively fewer personyears and deaths with missing educational level were assigned to the low educated (90.2% and 92.2%, respectively).

Figures S9-S13 demonstrate the effect of the proportional redistribution of missings. The effect on the share of deaths by educational level and the share of personyears by educational level is in line with our intentions (Figure S9-S10). In fact, the share of personyears and share of deaths by educational level are – after this adjustment – the same as the shares before the adjustment if those shares were calculated thereby not considering the missings. The effect of the redistribution on the age-specific mortality rates (Figure S11) and – consequently – the levels and trends in SDR by educational level is negligible (Figure S12), except for the elderly in the early 1970s. However, in terms of the CDR (Figure S13) we observe higher rates for the low educated, particularly in the 1972-1981 period, but also in the 2001-2011 period. This is because predominantly the elderly – with high mortality rates - had missing educational information, and their redistribution consequently resulted in higher overall deaths compared to the overall personyears, particularly for the low educated to which the vast majority of missings are redistributed. For the non-elderly, the CDR remained the same after the proportional redistribution of personyears and deaths with (remaining) missing educational level across the three educational groups.

Table S11. The proportional redistribution of personyears and deaths with (remaining) missing educational level across the three educational groups, for selected populations, by sex, England and Wales, 1972-2017.

Sex	Period	Agegroup	Missings	All	Percentage missings redistributed to		
					Low	Middle	High
			Personyears				
Males	1972-2017	30+	69657	6743080	87.8	6.4	5.8
Males	1972-1981	30-69	28177	1166892	85.2	9.2	5.6
Males	1972-1981	70+	27247	173824	94.7	2.3	3.0
Males	2002-2011	75+	5314	170346	85.3	6.3	8.4
Females	1972-2017	30+	114114	7629459	93.4	5.1	1.5
Females	1972-1981	30-69	34922	1217084	90.3	8.2	1.5
Females	1972-1981	70+	60992	310627	96.8	2.6	0.6
Females	2002-2011	75+	10521	265477	92.7	5.6	1.7
**Total**	**1972-2017**	**30+**	**183771**	**14372539**	**91.3**	**5.5**	**3.2**
Total	1972-1981	30-69	63099	2383976	88.0	8.7	3.3
Total	1972-1981	70+	88238	484451	96.2	2.5	1.3
Total	2002-2011	75+	15835	435823	90.2	5.8	4.0
			Deaths				
Males	1972-2017	30+	4855	124459	94.2	2.8	3.0
Males	1972-1981	30-69	486	12977	92.3	5.0	2.7
Males	1972-1981	70+	3440	16828	95.9	1.9	2.2
Males	2002-2011	75+	742	14896	88.2	5.2	6.6
Females	1972-2017	30+	7617	132954	96.8	2.6	0.6
Females	1972-1981	30-69	368	7780	94.0	5.2	0.8
Females	1972-1981	70+	5775	21720	97.6	2.0	0.4
Females	2002-2011	75+	1278	20801	94.5	4.2	1.3
**Total**	**1972-2017**	**30+**	**12472**	**257413**	**95.8**	**2.7**	**1.5**
Total	1972-1981	30-69	854	20757	93.0	5.1	1.9
Total	1972-1981	70+	9215	38548	97.0	2.0	1.0
Total	2002-2011	75+	2020	35697	92.2	4.6	3.2

Source data: ONS-LS

Figure S9. Trends in the share of deaths by educational attainment group (%) before and after the proportional redistribution of missings, for broad age groups (30+, 30-74, 75+), by sex, England and Wales, 1972-2017

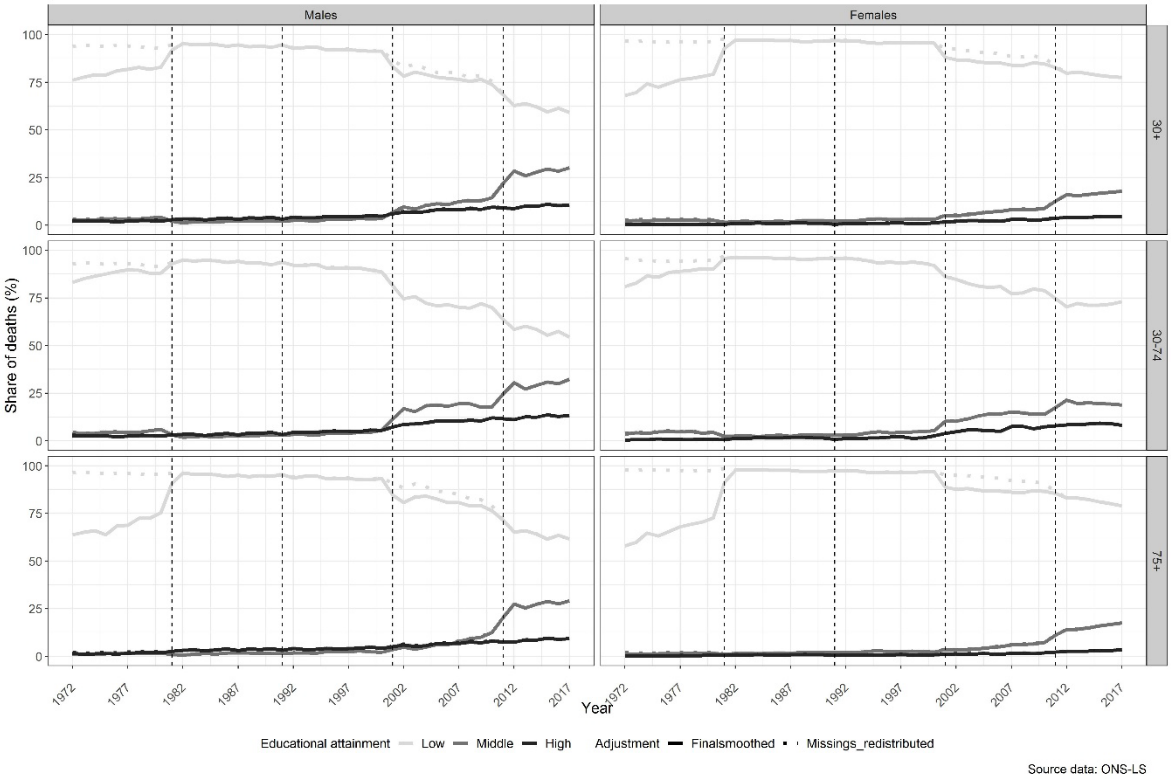

Figure S10. Trends in the share of personyears by educational attainment group (%) before and after the proportional redistribution of missings, for broad age groups (30+, 30-74, 75+), by sex, England and Wales, 1972-2017

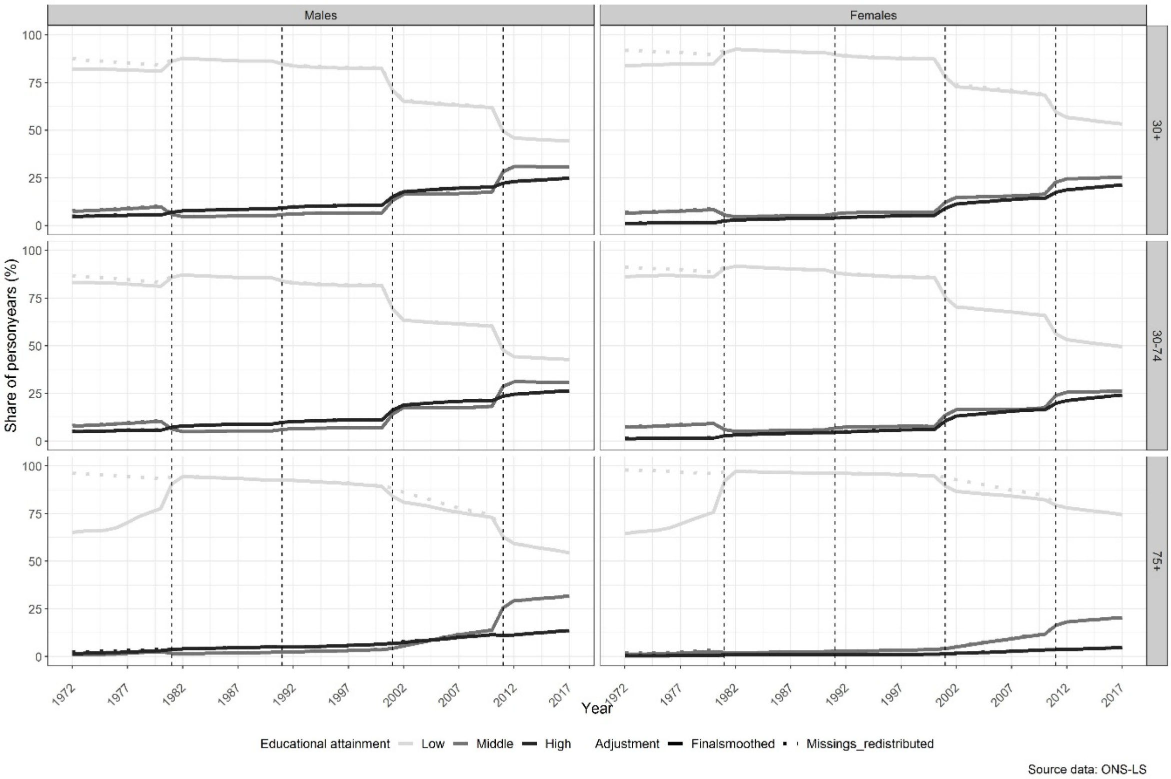

Figure S11. Age-specific mortality rates (log scale) before and after the proportional redistribution of missings, by sex and educational group, England and Wales, selected years. *Age groups: 30 = 30-34, … , 90 = 90-94, 95 = 95+.*

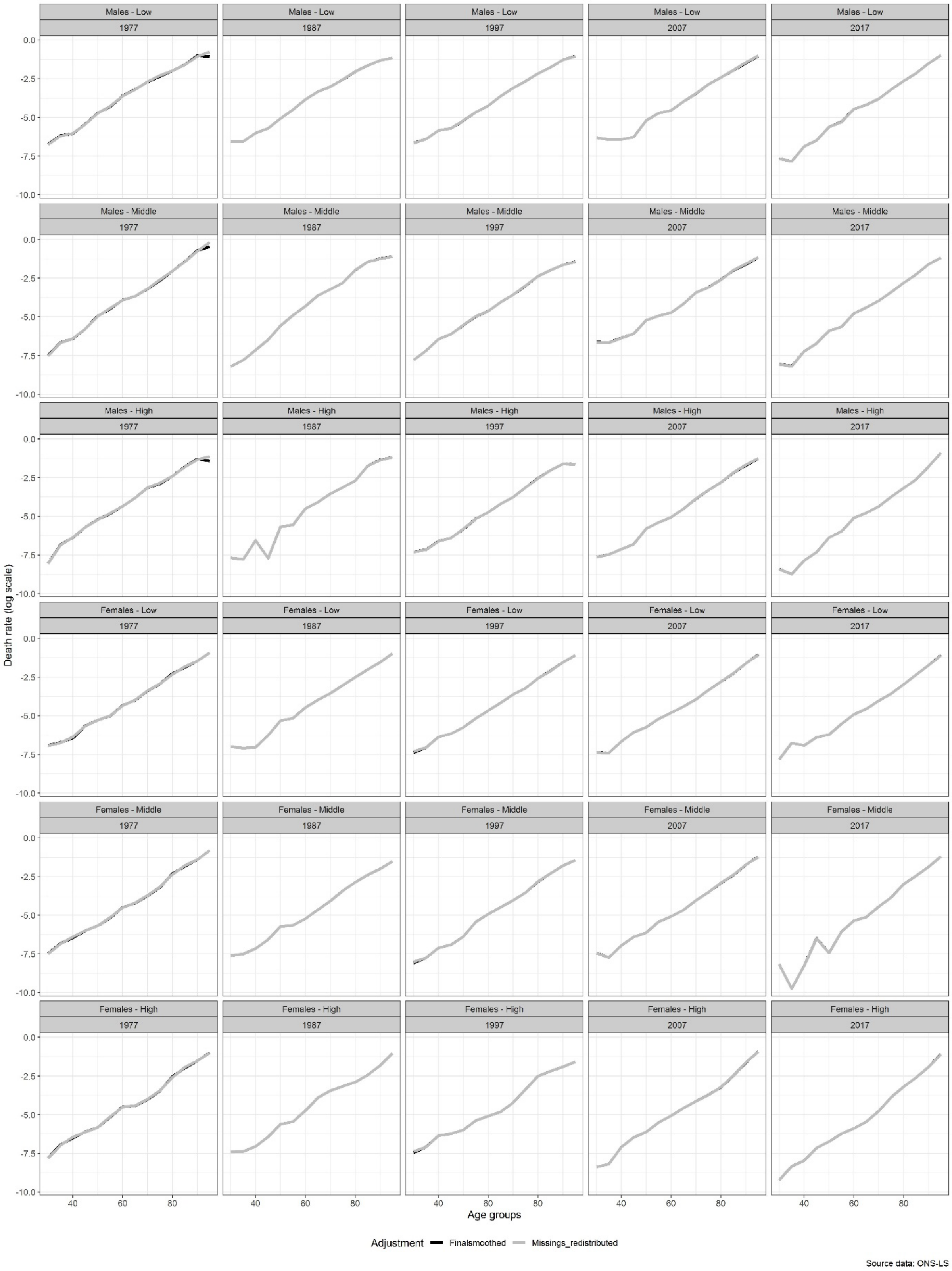

Figure S12. Trends in age-standardised mortality (SDR) by educational attainment group before and after the proportional redistribution of missings, for broad age groups (30+, 30-74, 75+), by sex, England and Wales, 1972-2017

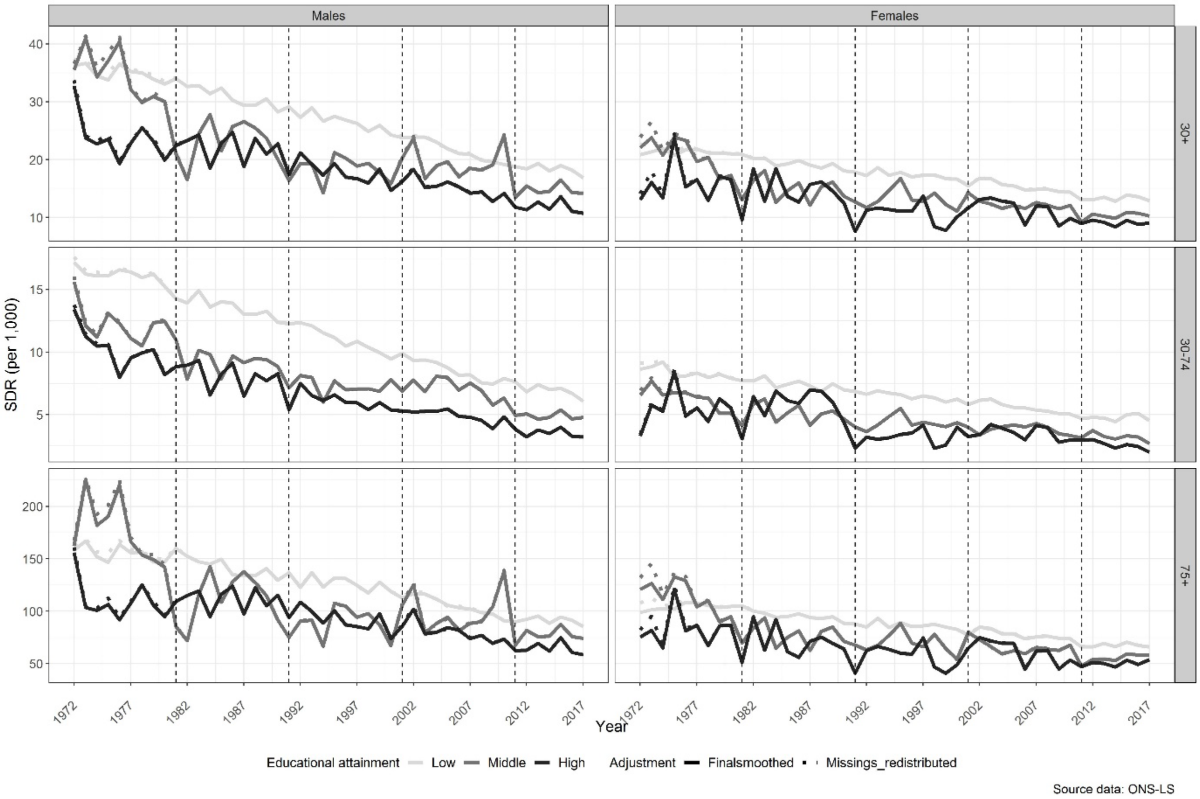

Figure S13. Trends in the crude death rate (CDR) by educational attainment group before and after the proportional redistribution of missings, for broad age groups (30+, 30-74, 75+), by sex, England and Wales, 1972-2017

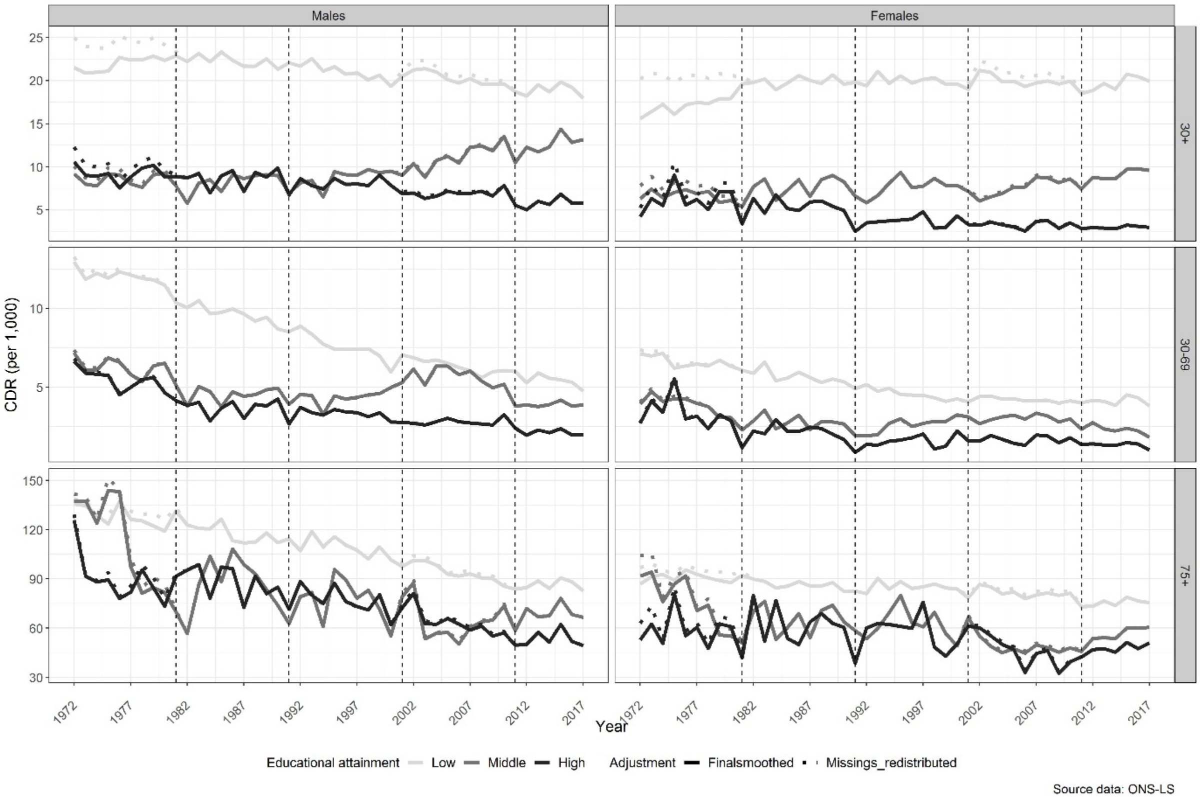

5.3 Careful study of trends over time

An important element of the remaining adjustments at the aggregate level was the careful study of trends over time. We identified discontinuities in trends over time that can be linked to the identified data issues, thereby exploiting the yearly character of the data and the available information on the data issues. We examined trends over time in the annual share of personyears and deaths by educational level (%), the crude death rate (CDR), and the age-standardised death rate (SDR) for individuals aged 30+, 30-74, and 75+.

Figure S14 shows the trends in the share of personyears by educational attainment (%), from 1972 until 2017 for different main age groups (30+, 30-74, 75+). Based on comparable data for Italy (Turin) and Finland (see Appendix III), we expected decreases in the share of personyears for the low educated with downward shifts at the census years (1981, 1991, 2001, 2011), and increases with upward shifts at the census years for the middle and high educated, in line with educational expansion and with the fact that the educational information is not continuous but updated at census years. That is, the educational information in a year before the census does not represent the actual educational distribution, but the situation 9 years earlier.

However, for individuals aged 30 and older, we observed an upward shift in the share of personyears for the low educated in 1981 resulting in elevated shares for 1981-2001, and a concurrent downward shift in the share of personyears for the middle educated in 1981, resulting in lowered shares for 1981-2001. For the share of deaths by educational level we observed a similar pattern (Figure S15), whereas we would, in line with the observations for Italy (Turin) and Finland in Appendix III, expect continuous decreases for the low educated, and continuous increases for the middle and high educated.

These unrealistic shifts in the share of personyears and deaths by educational level in 1981 can be brought back to the remaining inconsistency in the educational classification over time that we were not able to tackle at the individual level. This applies to the incorrect classification of ONS-LS members with upper secondary degrees in the 1981 and 1991 censuses as low instead of middle educated because of the lack of information on upper secondary degrees. Whereas for individuals aged 20-74, its effect is visible from 1981 until 2001 in line with the 10 year follow-up of ONS-LS members at the 1981 and 1991 censuses; for individuals aged 75 and older, the effect lasts even up until 2011, because for individuals aged 75 and older in 2001, who all had missing educational information, we adopted the educational information from the 1991 Census, and consequently the incorrect classification applies as well to individuals aged 75 and older in the 2001 follow-up until 2011.

Another trend discontinuity in the share of deaths for the low educated (downwards) and the middle educated (upwards) occurred in 2011 for the three broad age groups and both sexes. Because we essentially adopted the same educational classification in 2011 compared to 2001, we believe that these shifts are probably not due to educational classification issues. Also, we do not expect that they are explained by our different emigration definition from 2011 onwards, because, although the underestimation of emigrants and overestimation of personyears is likely more severe among the low educated, this cannot in itself result in such large jumps. Since we also see strong shifts in the share of personyears by educational level in 2011, which we did not observe for Italy and Finland (see Appendix III), we expect that these strong shifts in 2011 are likely explained by the huge educational expansion that England & Wales experienced in the early 2000s (Greenaway & Haynes, 2003), and therefore not unrealistic.

Finally, the trends in both the share of personyears and of deaths for the high educated prove rather consistent over time since we were able to identify ONS-LS members with a tertiary degree or higher across all the censuses, thereby using the summarized educational variables for the 1971, 1981 and 1991 censuses and the underlying educational variables for the 2001 and 2011 censuses.

Figure S14. Trends in the share of personyears by educational attainment group (%) after smoothing and redistribution of missings, for broad age groups (30+, 30-74, 75+), by sex, England and Wales, 1972-2017

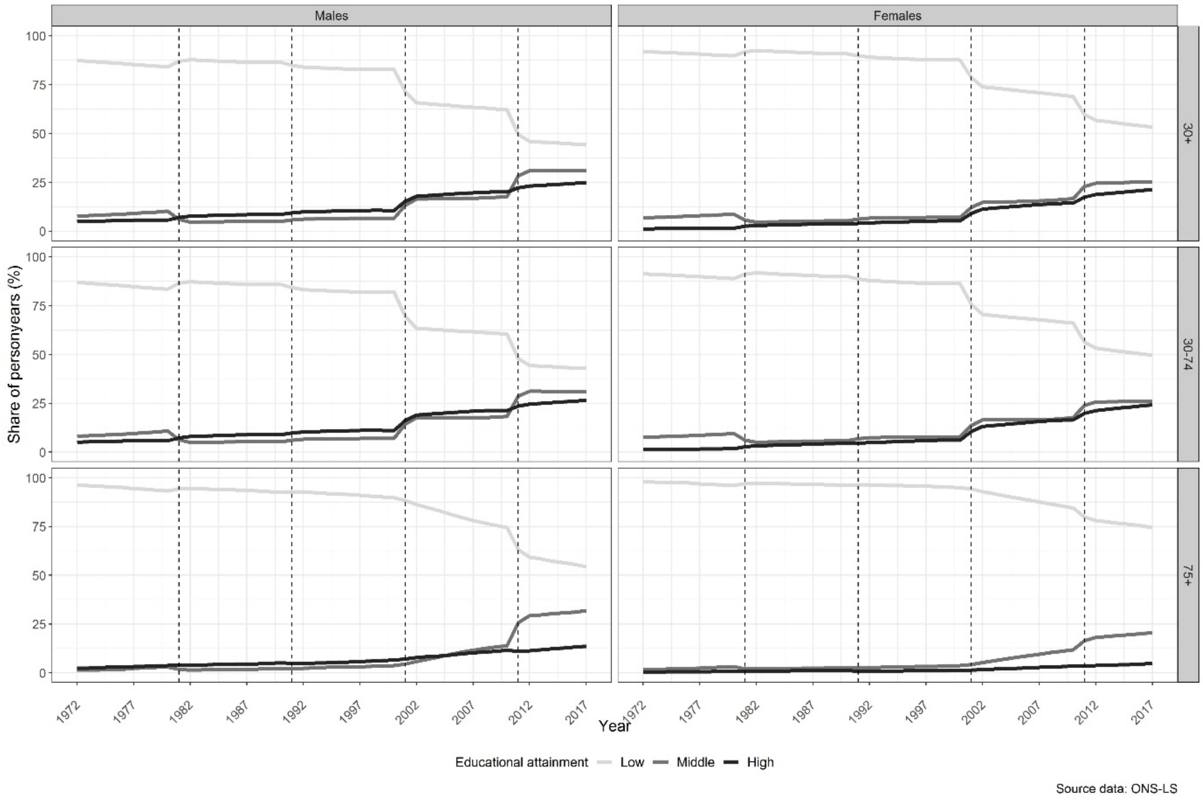

Figure S15. Trends in the share of deaths by educational attainment group (%) after smoothing and redistribution of missings, for broad age groups (30+, 30-74, 75+), by sex, England and Wales, 1972-2017

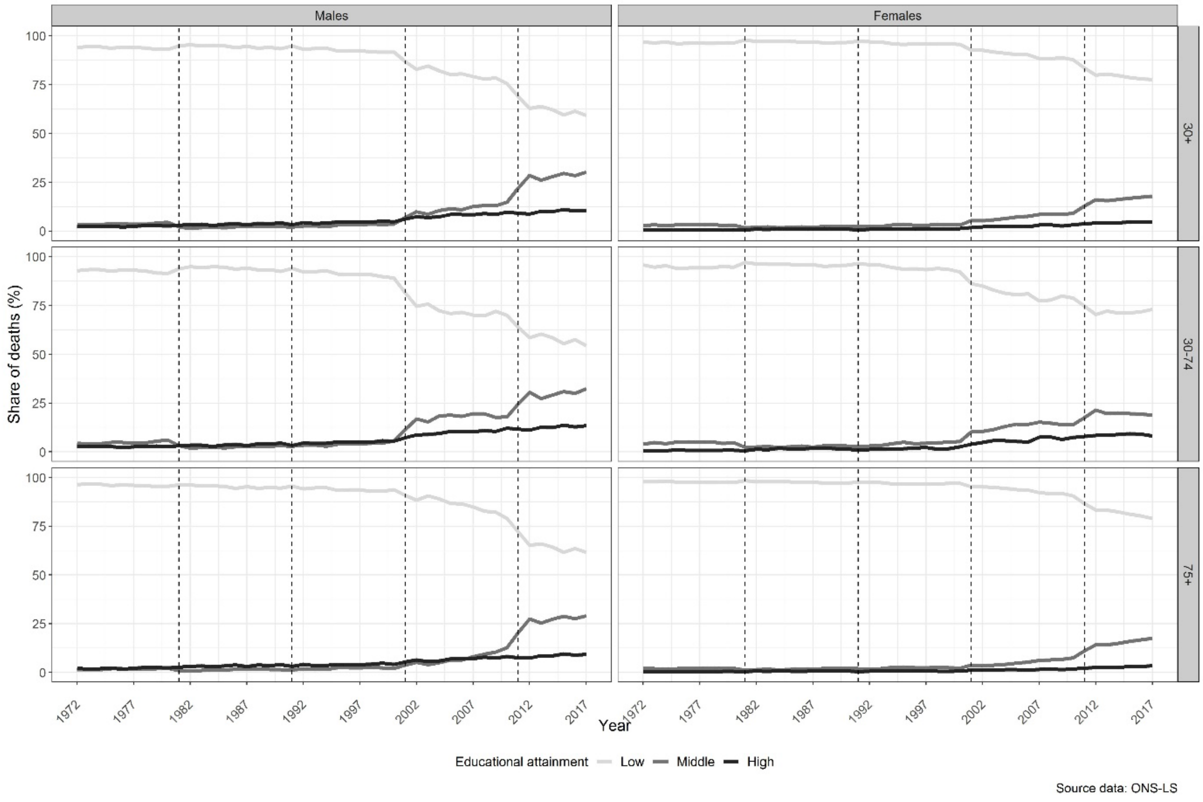

Figure S16. Trends in the crude death rate (CDR) by educational attainment group after smoothing and redistribution of missings, for broad age groups (30+, 30-74, 75+), by sex, England and Wales, 1972-2017

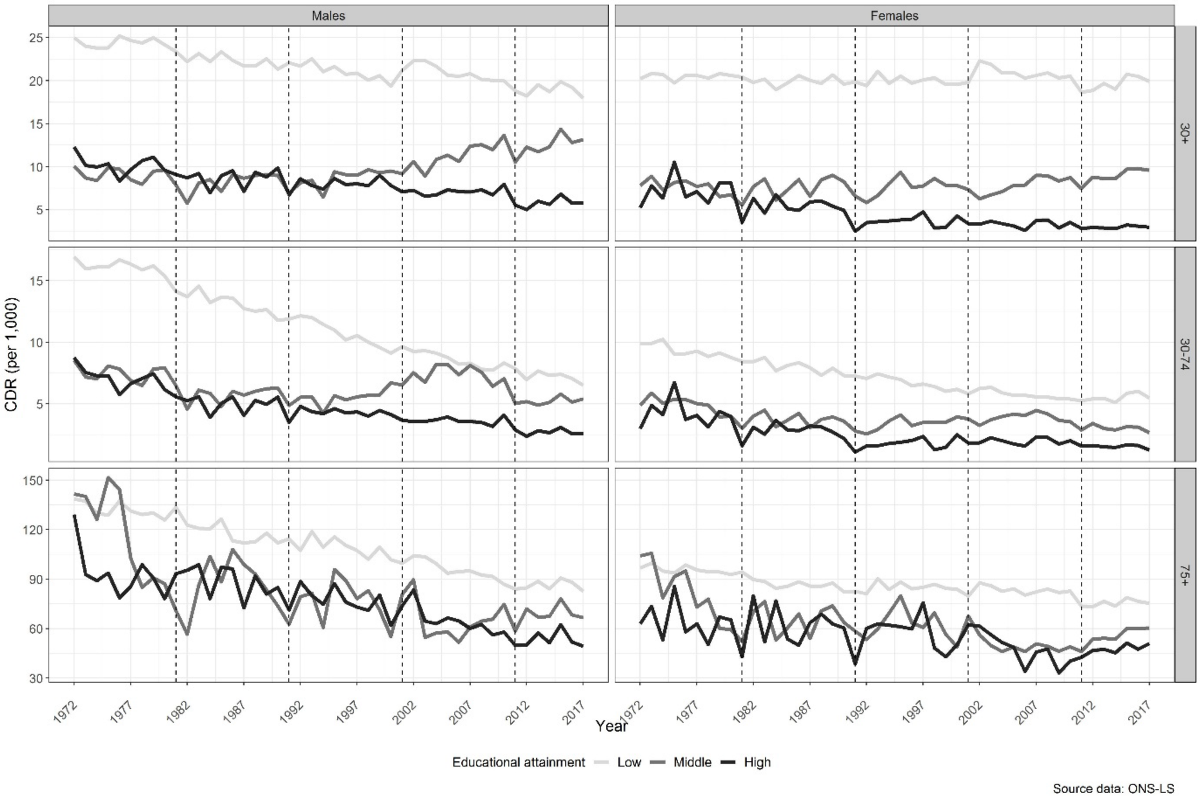

Figure S17. Trends in age-standardised mortality (SDR) by educational attainment group after smoothing and redistribution of missings, for broad age groups (30+, 30-74, 75+), by sex, England and Wales, 1972-2017

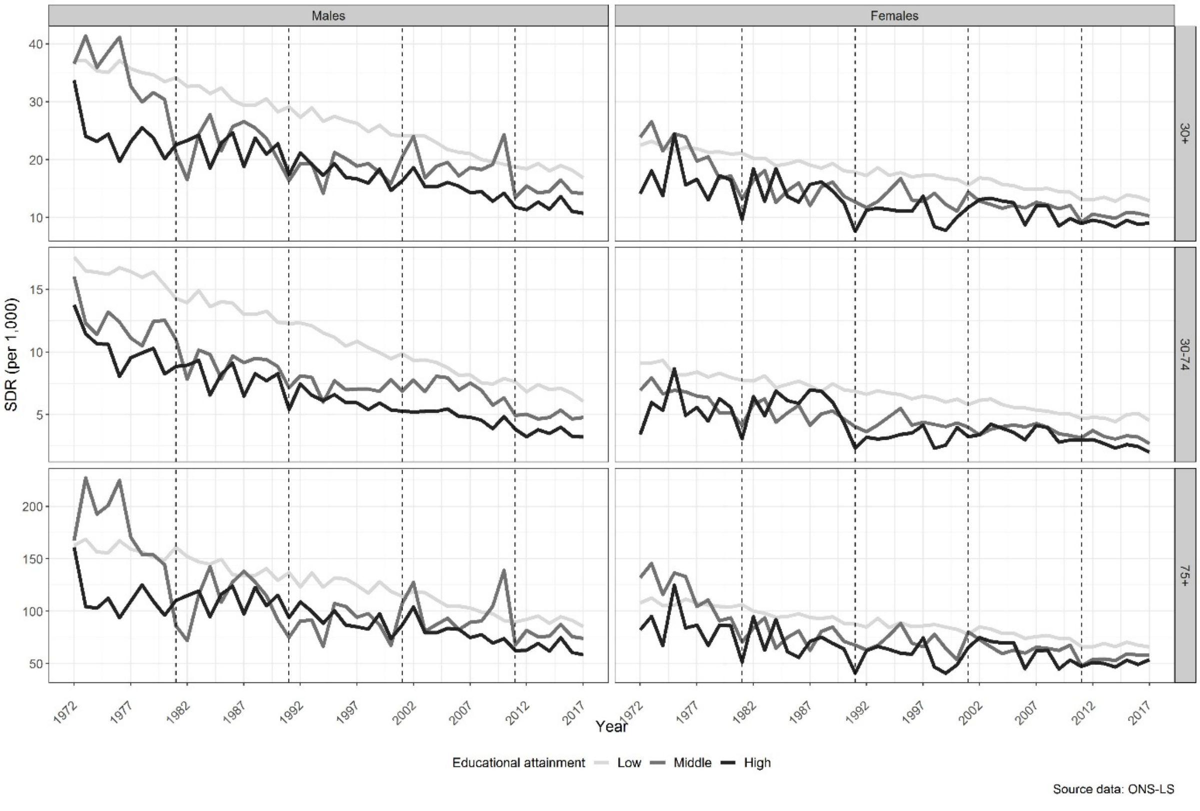

Trends over time in CDR and SDR are a bit erratic for the middle and high educated because of low numbers (Figure S16-S17). Still, for the low educated, it is possible to discern some jumps at the census years. That is, it seems that for low educated men in the period 1981-2001 the CDR levels are lowered compared to before and after. These lowered CDR levels are in line with the expected underestimation of mortality because of the misclassification of part of the middle educated (with lower mortality) to the low educated. However, for women, and for the SDR this cannot be observed.

5.4 Adjustment of the trend break in 1981

Our analysis of the trends over time revealed data-inconsistency driven jumps in the share of personyears and the share of deaths for the middle and the low educated in 1981, that lasted for the 1981-2001 period for those aged 30-74 and for the 1981-2011 period for those aged 75+.

We adjusted for this trend break by using existing approaches to deal with trend breaks in cause-specific mortality resulting from revisions in the International Classification of Diseases (Janssen & Kunst, 2004; Van der Stegen et al. 2014). The basic idea of these approaches is to shift (upwards or downwards) the levels before or after an unrealistic jump to the levels following or preceding the jump, respectively (depending on which levels are considered correct), so that the trend break no longer exist, and the original trends following and preceding the jump are maintained. Figure S18 illustrates this.

Figure S18. Illustration of the general approach to adjust trend breaks over time

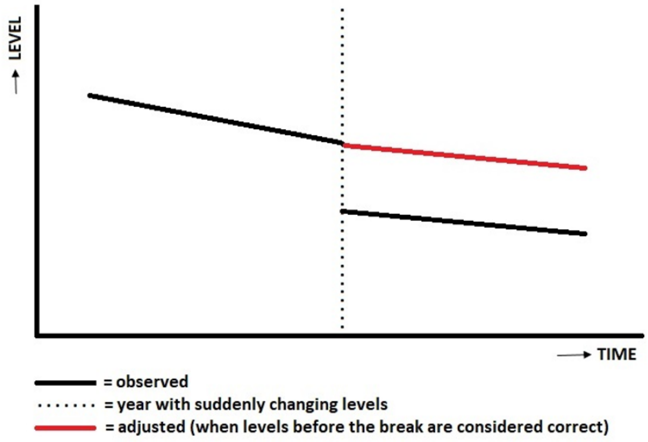

We used a two-step approach to adjust for this trend break in 1981. First, we adjusted, by sex, for the trend breaks in the age-specific shares of person-years by educational level for the middle educated in 1981 to obtain a yearly estimate of the age-specific person-years to be redistributed between the low and the middle educated. Second, we calculated the age-specific deaths to be redistributed by year and sex by multiplying the redistributed age-specific personyears with the smoothed age-specific mortality rates for the middle educated.

The advantage of using this two-step approach compared to adjusting trend breaks in age-standardised or age-specific mortality rates, is that for the latter approach it remains unclear how the underlying personyears and deaths should be readjusted in line with the adjusted mortality rates. Also, the direct adjustment of age-specific or age-standardised mortality rates might not result in similar total number of deaths and personyears before and after the adjustment. Lastly, it is rather difficult to observe the trend breaks in either the age-specific or the age-standardised mortality rates,

whereas the breaks in the (age-specific) share of personyears (and deaths) by educational level are very clear and can directly be related to the inconsistencies in the educational variable over time.

We choose to focus on the share in personyears by educational level – instead of the share of deaths by educational level - because we regard the effect of erroneously classifying people first and foremost a matter of people (and not deaths) getting reallocated to a different group. Also, the age-specific shares of personyears by educational level are more robust over time compared to the age-specific shares of deaths by educational level.

We selected the middle educated for the calculation of the adjustment factors instead of the low educated, because for the middle educated it is safer to assume that educational expansion did not importantly affect the underlying trend in the share of personyears. Indeed, as can be observed in Figure S14, the low educated are more affected by educational expansion in the first three decades compared to the middle educated.

We adjusted the trend break in the share of personyears by five-year age group, because using broader age groups (30-74, 75+) for the estimation of adjustment factors resulted in unrealistic outcomes at the age-specific level, in particular for older women.

In applying our adjustment we adjusted the age-specific share of personyears among the middle educated after 1981 (1981-2001 for individuals aged 30-74; 1981-2011 for individuals aged 75 and older) upwards, so it matches the age-specific shares observed before 1981. This, because the educational classification in 1971 aligns well with the ISCED classification and the preferred distinction into low, middle and high. Furthermore, we took into account that the different censuses in ENW did not take place on January 1, but mostly around April 1 of the year. Consequently we focussed on comparing 1980 versus 1982 and applied the adjustment only partly in 1981 (all ages), 2001 (30-74), and 2011 (75+).

More details regarding the two steps of the adjustment follow below.

Step 1 - Adjustment of the trend break in 1981 in the age-specific shares of personyears (%) for the middle and low educated

In adjusting – by sex and five-year age group – for the trend break in 1981 in the share of personyears for the middle educated, we ensured that both the original trends before the break and the original trends after the break are maintained. That is, we only adjust the trend after the break upwards to match the trend before the break, based on the absolute difference between the two in 1982. We estimated this absolute difference in 1982, by comparing the extrapolated share in 1982 (based on linear regression of the trend for 1977-1980), with the fitted share in 1982 (obtained through linear regression applied to 1982-1985).

The resulting absolute difference between the fitted and the extrapolated share – which represents the absolute difference between the trend before and after the break - is subsequently applied to the observed shares of personyears for the middle educated after the break (1981-2001 for individuals aged 30-74, and 1981-2011 for individuals aged 75 and older), thereby applying the adjustment only partly in 1981 (all ages), in 2001 (30-74), and in 2011 (75+). That is, for all five-year age groups we implemented for 1981 (census date April 5) 365-(31+28+31+5)/365 = 270 / 365 = 0.74 of the absolute adjustment level in 1982. For individuals aged 30-74 we implemented for 2001 (census date April 29) (31+28+31+29)/365 = 119/365 = 0.33 of the absolute sex- and age-specific adjustment levels in 1982. For individuals aged 75 and older for 2011 (census date March 27) we implemented (31+28+27)/365 = 86/365 = 0.24 of the absolute sex- and age-specific adjustment levels in 1982.

We subsequently obtained the to-be redistributed age- and sex specific personyears from the low educated to the middle educated by multiplying the adjusted shares of personyears for the middle educated to the age- and sex specific personyears for the three educational groups combined. Subsequently these to-be-redistributed personyears were added to the personyears for the middle educated and subtracted from the personyears for the low educated.

See Table S12 for the age-specific adjustment levels in 1982, and the number of personyears we redistributed from the middle to the low educated in that year.

Table S12. The age-specific adjustment levels for the share of personyears for the middle educated in 1982, and the associated number of personyears we redistributed from the low to the middle educated in that year, by sex, England and Wales

	Males		Females	
	**Adjustment level (%)**	**Redistributed personyears**	**Adjustment level (%)**	**Redistributed personyears**
30-34	13.6	2323	8.5	1487
35-39	8.2	1415	6.8	1172
40-44	6.0	885	5.0	713
45-49	7.0	1008	5.0	698
50-54	5.4	781	4.2	615
55-59	4.3	628	3.5	533
60-64	4.9	653	4.6	702
65-69	4.3	488	3.5	493
70-74	4.1	385	4.0	512
75-79	3.0	173	2.6	261
80-84	1.3	35	1.6	101
85-89	0.0	0	0.5	16
90-94	0.0	0	0.0	0
95+	2.1	0	0.0	0
30+	8774 (6.4 %)		7304 (4.7 %)	

Source data: ONS-LS

Step 2 - Subsequent adjustment of the age-specific deaths for the middle and low educated

For the subsequent adjustment of the deaths, we multiplied – by year and sex – the redistributed age-specific personyears with the age-specific mortality rates of the middle educated to obtain the to-be-redistributed age-specific deaths. These were added to the age-specific deaths for the middle educated and subtracted from the age-specific deaths for the low educated.

We used the age-specific mortality rates of the middle educated in line with the observation that it was middle educated that were wrongly classified as low educated in the 1981 and 1991 censuses. However, because of the low numbers of middle educated in 1981-2001 in the data, the age-specific mortality rates for the middle educated were not very robust. Their application hence resulted in unrealistic high numbers of to be redistributed deaths from middle to low educated for specific years, and consequently unrealistic peaks in the adjusted SDR for the low educated. We therefore used smoothed age-specific mortality rates for the middle-educated instead based on the application of year- and sex-specific age schedules of mortality rates for the total England & Wales population in the

HMD, which we adjusted so that it matches a three-year moving average SDR for the middle educated in ONS-LS. We thereby set the maximum SDR ratio (SDR middle educated/SDR HMD) to 1 to ensure that the smoothed SDR for the middle educated is lower than (or equal to) the SDR for the total population in the HMD, in line with what can be expected given that the low educated comprise the largest share of the total population, and exhibit substantially higher mortality compared to the middle and the high educated.

Our adjustment for the unrealistic jumps in the share of personyears and share of deaths for the low and middle educated resulted - for individuals aged 30 and older - in the redistribution of 335,507 personyears and 2,638 deaths from low educated to middle educated in the 1981-2011 period (Table S14).

Figures S19-S25 illustrate the effect of our adjustment.

Figures S19 and S20 illustrate that our adjustment solved the sudden jumps in the share of personyears by educational level for both the middle educated and the low educated in 1981. For the middle educated (because we focussed on them) we now no longer see jumps, whereas for the low educated we now observe a small drop downwards - instead of the original jump upwards. This drop downwards counterbalances the jump upwards for the high educated and is in line with educational expansion in England & Wales (Greenaway & Haynes 2003; Paterson 2022b). This educational expansion is also responsible for the remaining jumps in the share of personyears in 2001 and 2011.

Figures S21 and S22 illustrate that applying the smoothed age-specific mortality of the middle educated to the redistributed personyears results in likely trends in the share of deaths by educational level. Note that because of unrealistic outcomes for males aged 30-34 in 1984 (due to very low numbers) we applied the average share of deaths over 1983 and 1985 to middle educated men aged 30-34 in 1984, and subsequently adjusted the share of deaths for the low educated.

In terms of the CDR by educational level (Figure S23), the levels for the low educated are adjusted upwards, and our adjustment thus resolved the seemingly lowered levels among the low educated in 1981-2001, which was particularly visible for men. In addition, the CDR for the middle educated has changed somewhat which can be related to use of smoothed instead of observed age-specific mortality rates to obtain the deaths for the redistributed personyears. Similar slight changes in the SDR for the middle educated can be observed in Figure S24. The change in the SDR for the low educated is quite small. The age-specific death rates did not change for the low educated in the 1981-2001 period, whereas a small change can be observed for the middle educated (see Figure S25; 1987 and 1997 are indicative for the 1981-2001 period) due to the use of smoothed instead of observed age-specific mortality rates for the adjustment.

Because we adjusted the share of personyears, and subsequently redistributed the personyears, the total personyears for the educational groups combined and the total deaths for the educational groups combined are not affected. For the educational groups combined, the age-specific mortality rates, CDR and SDR have therefore remained the same.

Figure S19. Age-specific trends in the share of personyears by educational attainment group (%) before and after adjustment trend break 1981, by sex, England and Wales, 1972-2017. a) males b) females. *Age groups: 30 = 30-34, … , 90 = 90-94, 95 = 95+.*

a) males

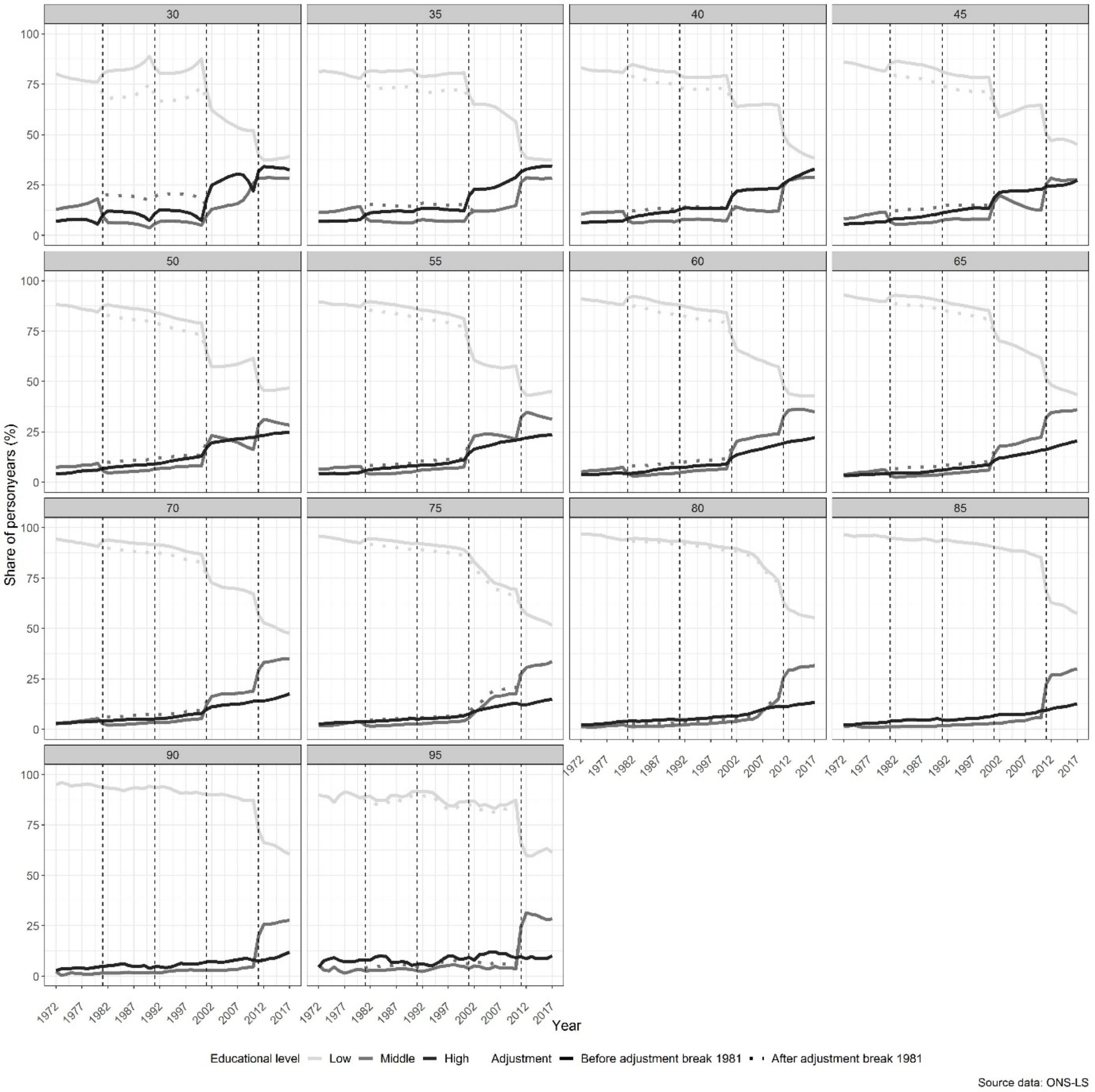

b) females

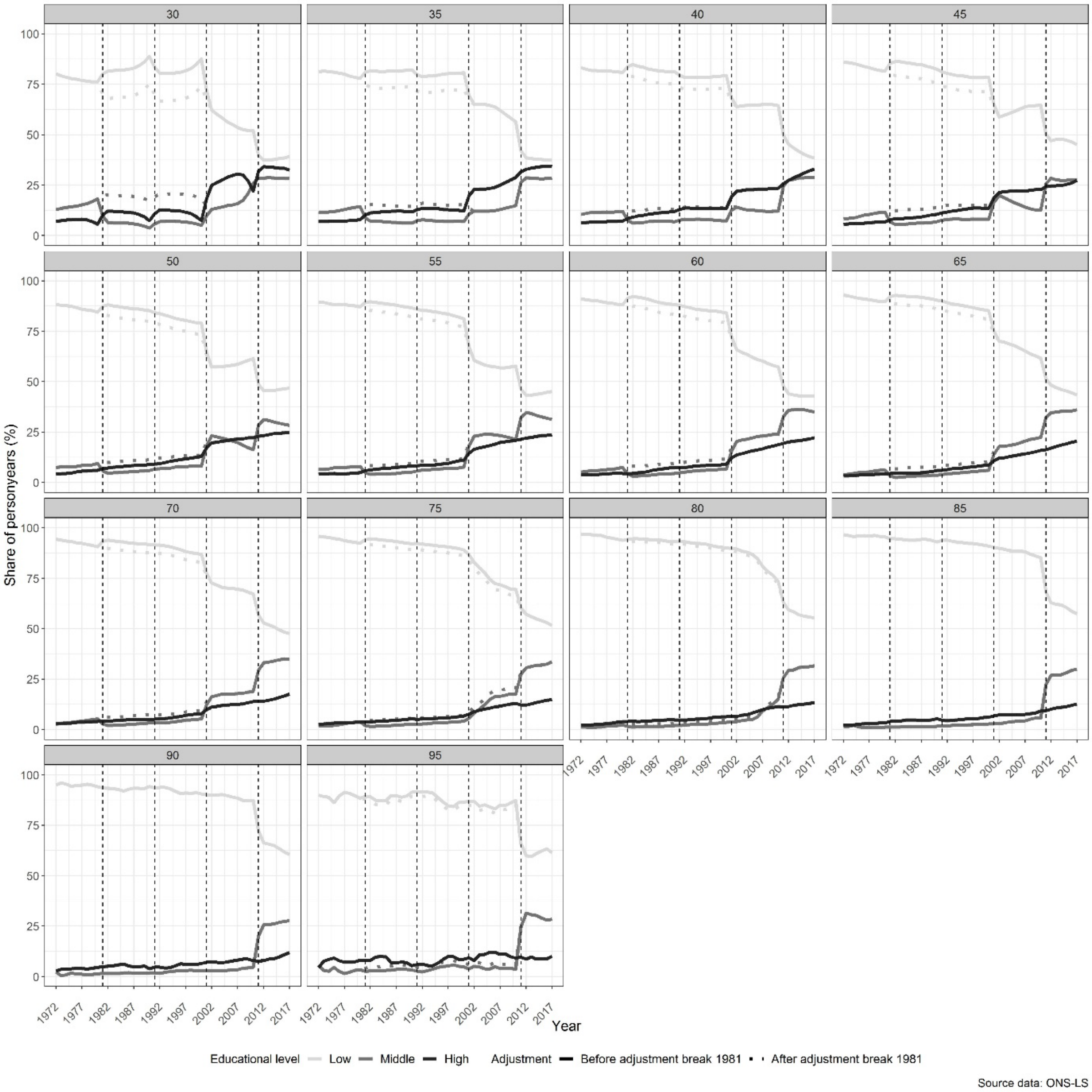

30
35
40
45
50
55
60
65
70
75
80
85
90
95
Share of personyears (%)
Year
Educational level Low Middle High
Adjustment Before adjustment break 1981 After adjustment break 1981
Source data: ONS-LS

Figure S20. Trends in the share of personyears by educational attainment group (%) before and after adjustment of the trend break in 1981, for broad age groups (30+, 30-74, 75+), by sex, England and Wales, 1972-2017

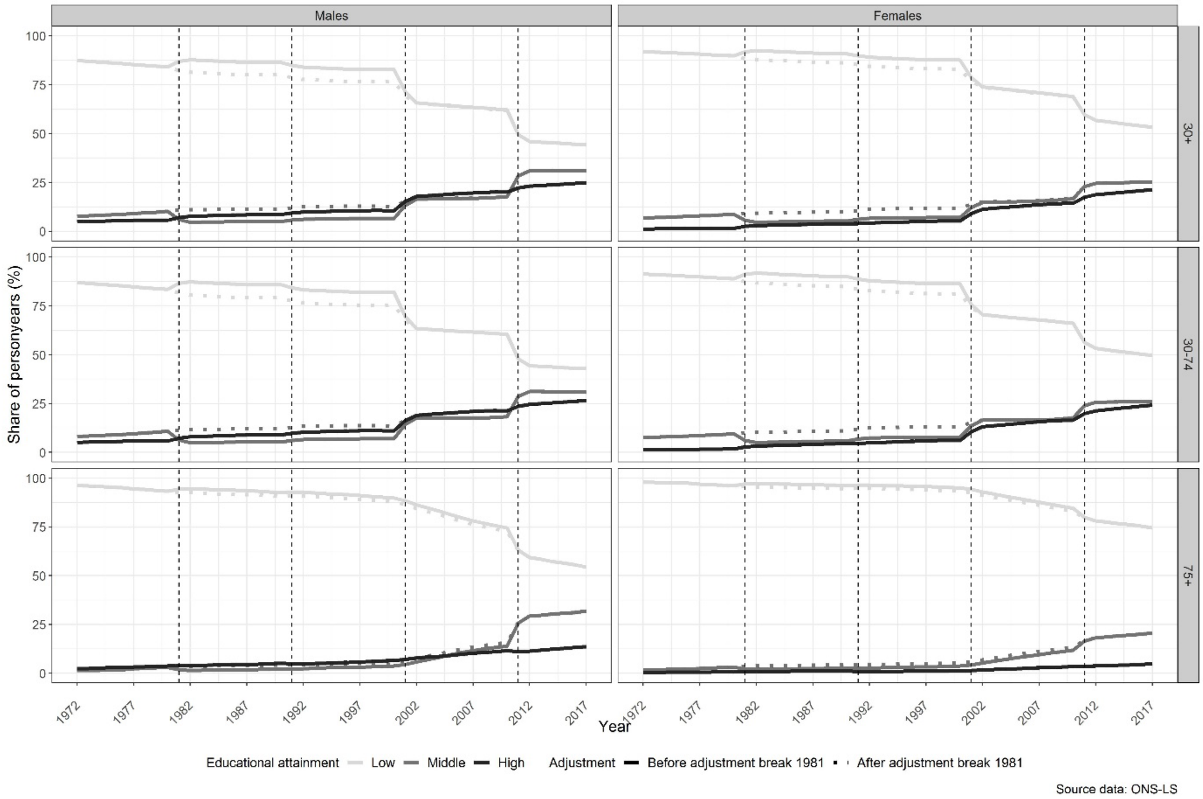

Figure S21. Trends in the share of deaths by educational attainment group (%) before and after adjustment of the trend break in 1981, for broad age groups (30+, 30-74, 75+), by sex, England and Wales, 1972-2017

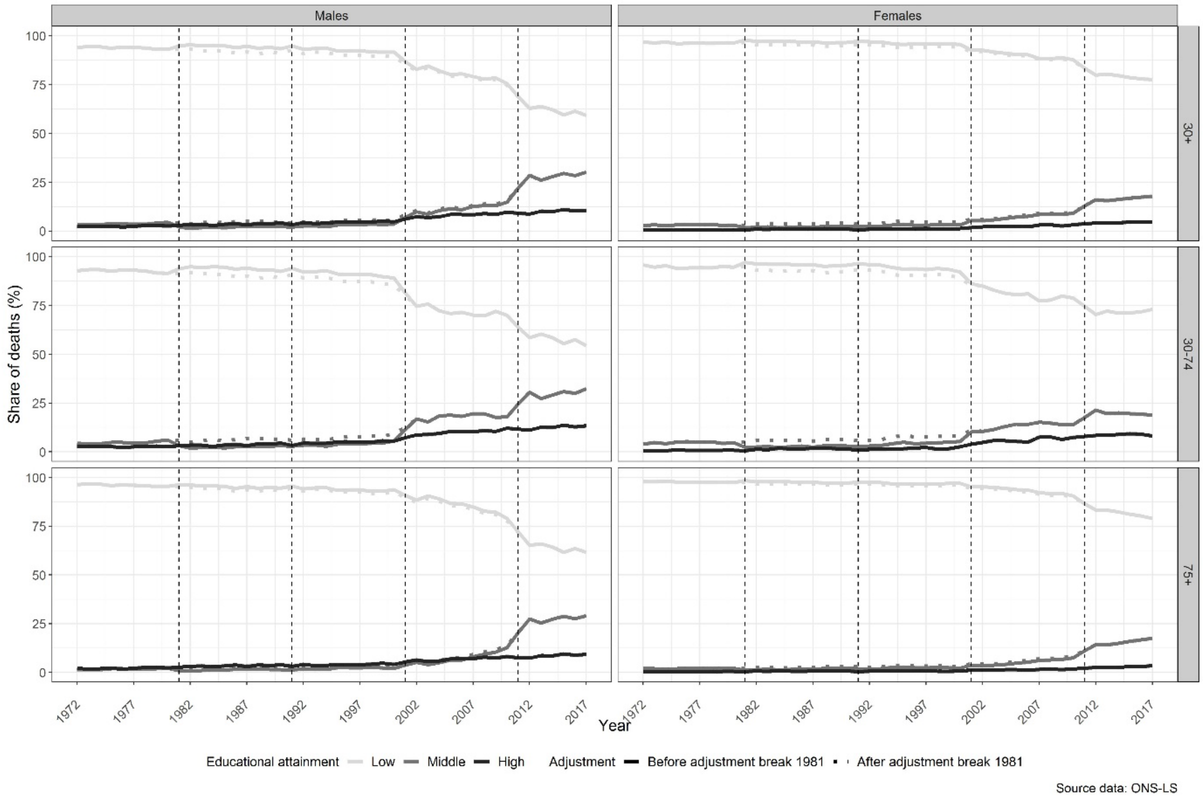

Figure S22. Age-specific trends in the share of deaths by educational attainment group (%) before and after adjustment of the trend break in 1981, by sex, England and Wales, 1972-2017. a) males b) females. *Age groups: 30 = 30-34, … , 90 = 90-94, 95 = 95+.*

a) males

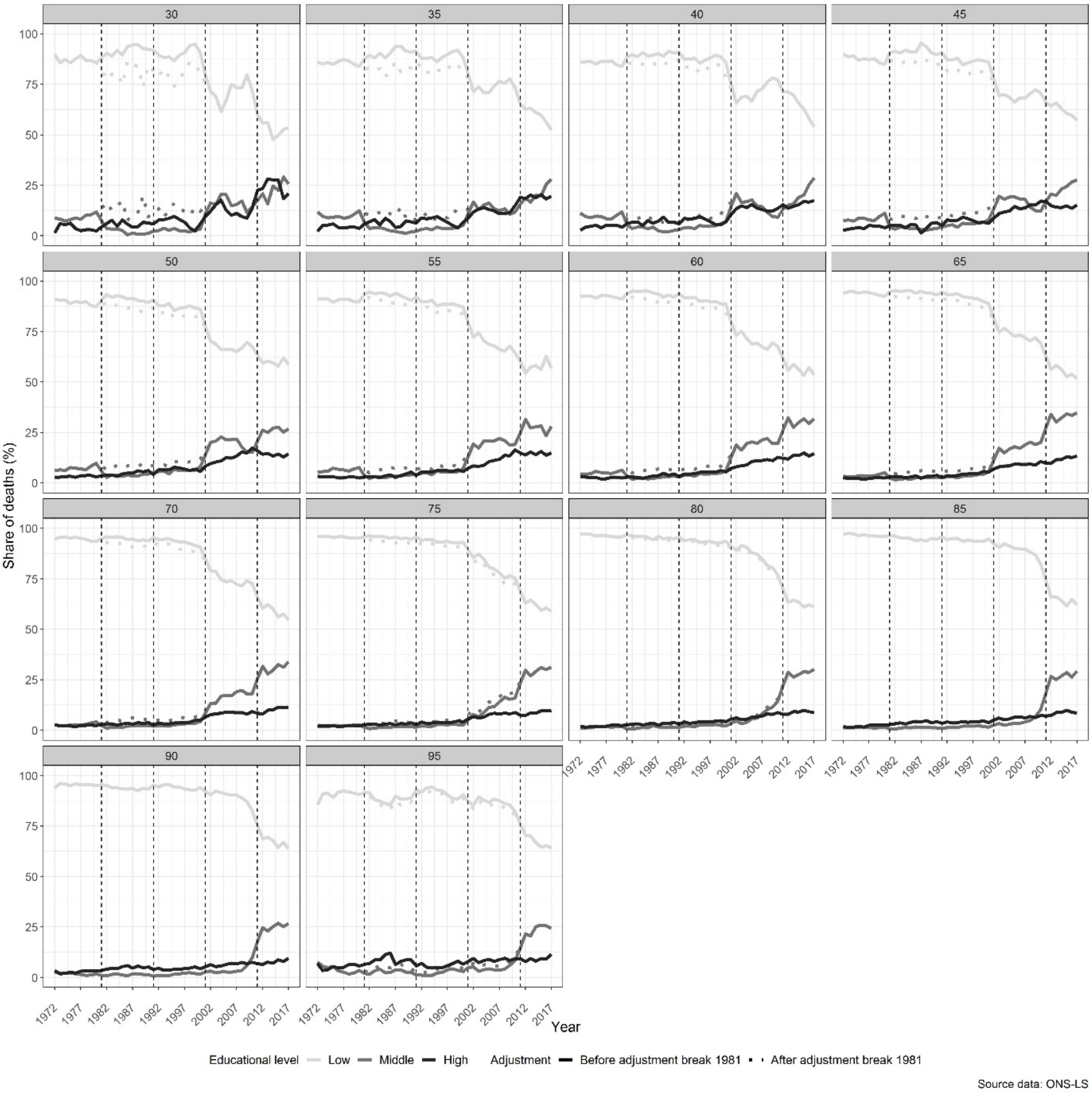

b) females

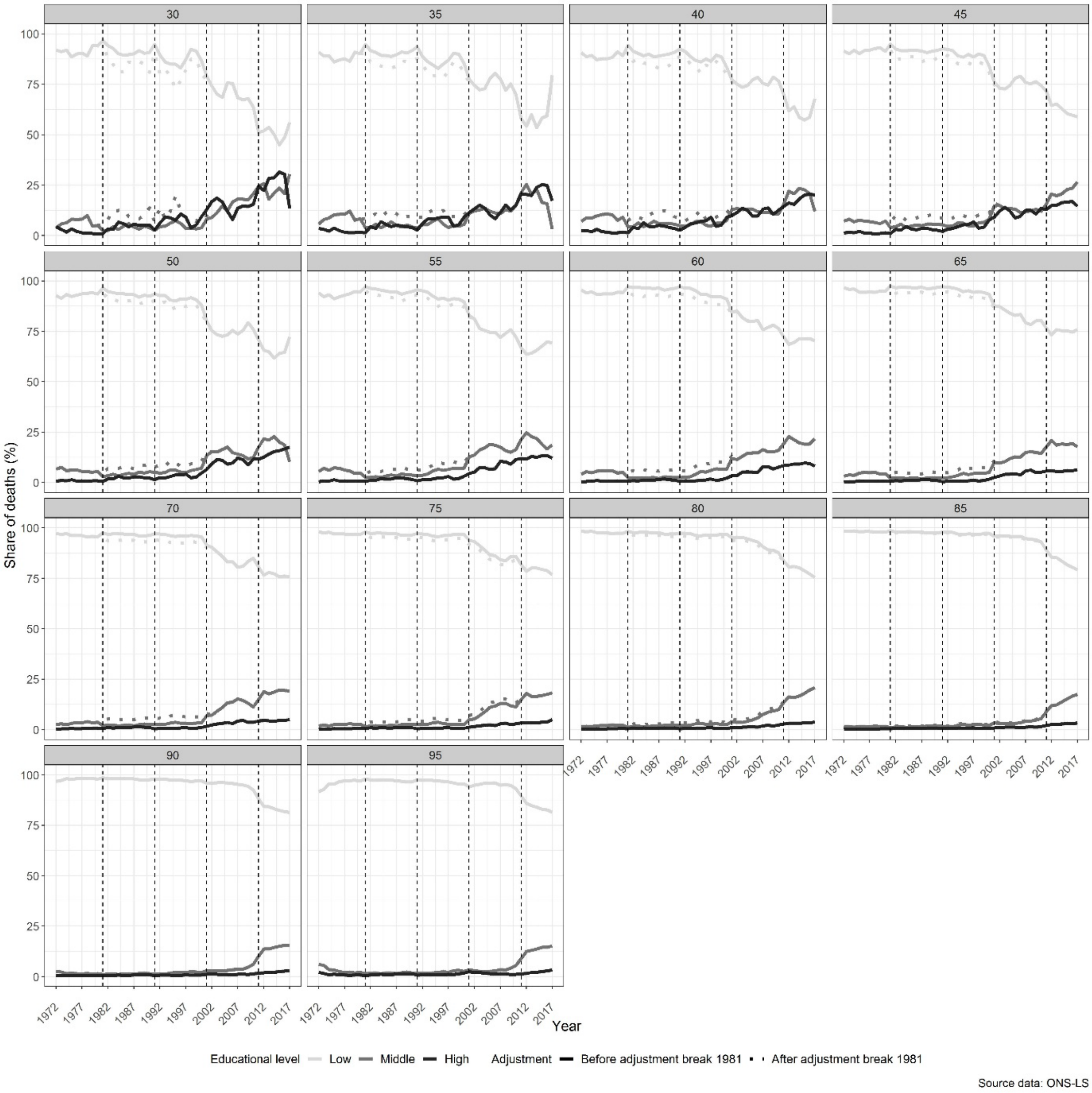
30
35
40
45
50
55
60
65
70
75
80
85
90
95
Share of deaths (%)
100
75
50
25
0
1972
1977
1982
1987
1992
1997
2002
2007
2012
2017
Year
Educational level
Low
Middle
High
Adjustment
Before adjustment break 1981
After adjustment break 1981
Source data: ONS-LS

Figure S23. Trends in the crude death rate (CDR) by educational attainment group before and after adjustment of the trend break in 1981, for broad age groups (30+, 30-74, 75+), by sex, England and Wales, 1972-2017

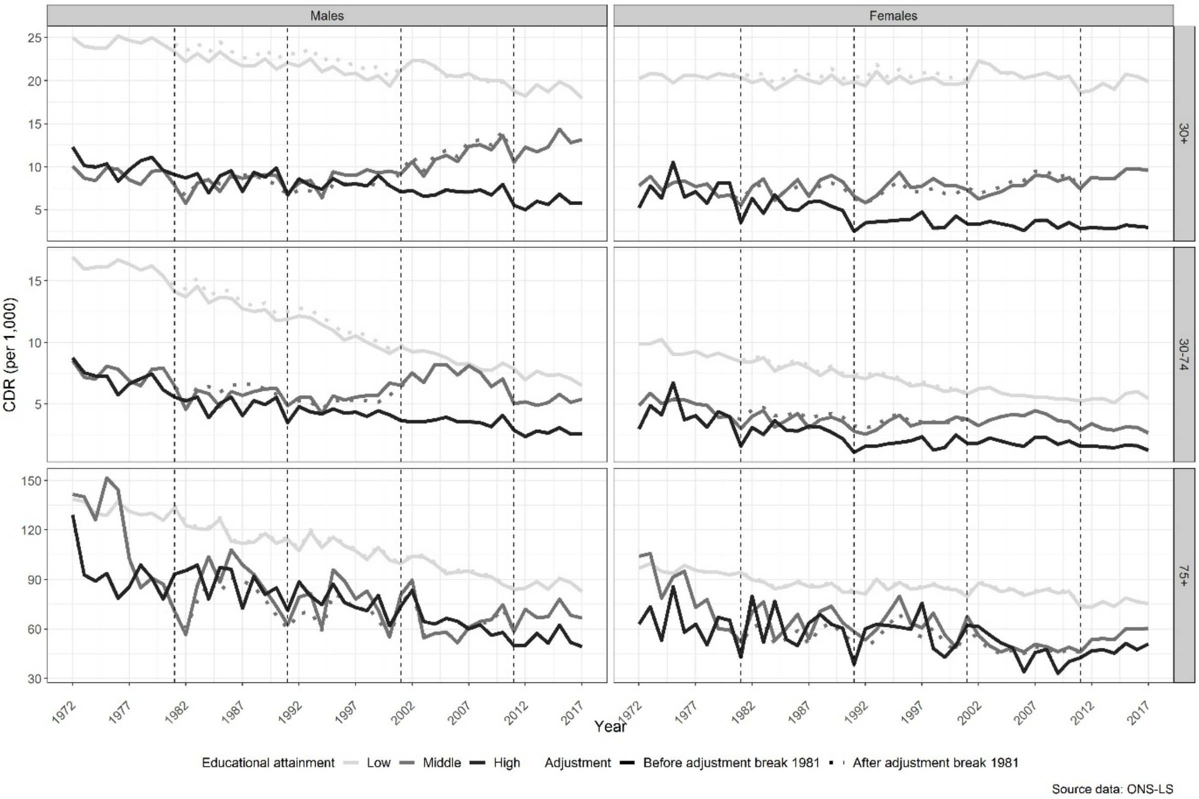

Figure S24. Trends in age-standardised mortality (SDR) by educational attainment group before and after adjustment of the trend break in 1981, for broad age groups (30+, 30-74, 75+), by sex, England and Wales, 1972-2017

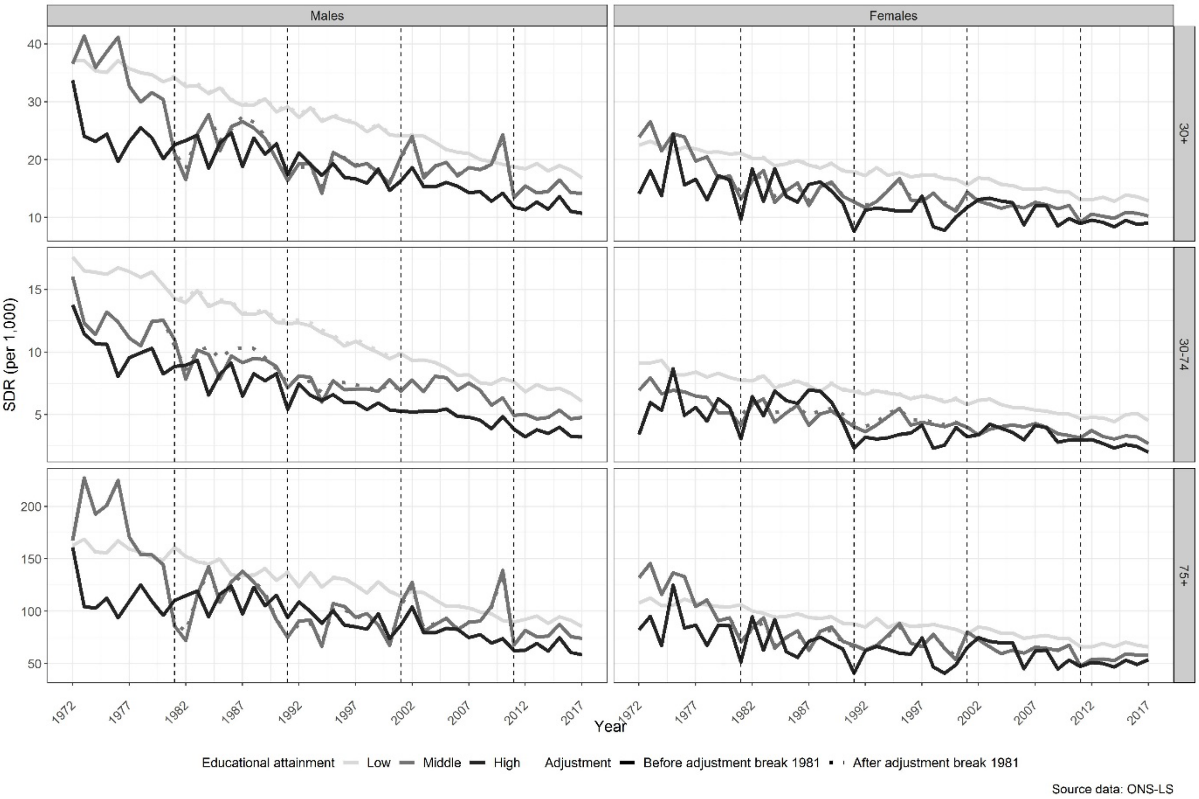

Figure S25. Age-specific mortality rates (log scale) before and after adjustment of the trend break in 1981, by sex and educational group, England and Wales, selected years. *Age groups: 30 = 30-34, … , 90 = 90-94, 95 = 95+.*

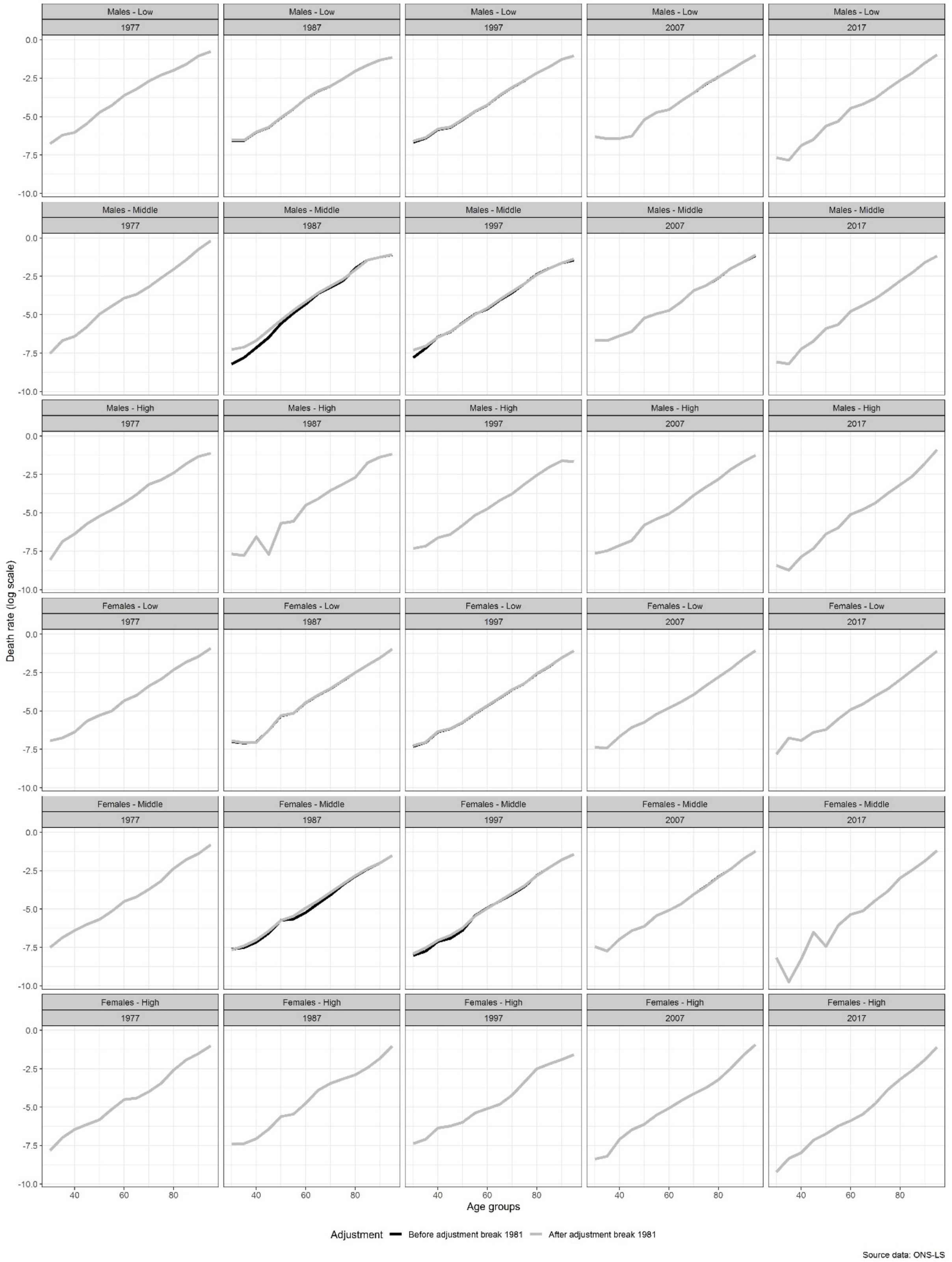

5.5 Correcting for the inevitably inconsistent definition of emigration over time

We corrected for our use of a different definition of emigration after 2011 (based on reported embarkations) compared to before (based on the absence of an individual in the next census) by applying, by sex, the age- and educational distribution of the embarkations in 2011-2017, to the estimated number of "missed" emigrants (= overestimated personyears) in that same period. We thereby corrected for the difference in sample size between the 2011 follow-up and the 2001 follow-up and for educational differences in reporting emigration. Next, we calculated by how much the deaths were overestimated by applying the observed strata-specific death rates to the age-, sex-, year-, and education-specific overestimated personyears.

As a first step we estimated, separately by sex, the number of "missed" emigrants aged 30 and older as the jump in the personyears between 2012 and 2010 that cannot be attributed to the difference in sample size between the 2011 and 2001 follow-up. We did so by subtracting from the observed difference in personyears between 2012 and 2010 (representing the "unrealistic" jump in personyears) the difference in the sample size between the 2011 and the 2001 ONS-LS follow-up periods (representing part of the jump not related to the inconsistent emigration definition). We choose this approach rather than applying the ratio between the estimated emigrants in 2001-2011 according to the two emigration definitions, because these ratios change over time (Table S3), and because the distribution by age group and educational level of the emigrants based on embarkations differs between 2001-2011 and 2011-2017 (Figure S26a). We used the broad age group 30 and older, because at the age-specific level it was difficult to discern the different effect of the inconsistent emigration definition and the effect of the change in sample size between the 2001 and 2011 follow-up. Table S13 shows our estimation of the sex-specific number of "missed" emigrants aged 30 and older in 2011-2017.

Table S13. Estimation of the number of "missed" emigrants between 2011 and 2017 as the jump in the personyears between 2012 and 2010 that cannot be attributed to the difference in sample size between the 2011 and 2001 follow-up, by sex, aged 30 and older, England and Wales

sex	Difference in personyears between 2012 and 2010	Difference in sample size between the 2011 follow-up and the 2001 follow-up	Estimated number of "missed" emigrants
Males	30,131	15,952	14,179
Females	27,062	13,880	13,182
Total	57,193	29,832	27,361

Source data: ONS-LS

The estimated number of "missed" emigrants of 27,361 equals an EMI1/EMI2 ratio of (27,361+2,124)/2,124 = 13.88 (where 2,124 is the number of emigrants 30+ according to the EMI1/EMI2 definition in 2011-2017). This can be considered a reasonable estimate, given that in 2001-2011 this ratio was 12.19 (see Table S3). The estimated number of overestimated personyears represents about half of the total difference in personyears between 2010 and 2012 and 7.3% of the ONS-LS sample aged 30+ in 2011.

As a second step we estimated the sex-, age-, and education-specific overestimated personyears by applying the age- and education-specific distribution of reported emigrations (= embarkations") in 2011-2017 to the "missed" emigrants. In doing so, we corrected for the difference in reporting embarkations between the high and the low educated. In doing so, we selected the total population distribution (= for men and women combined), because there are hardly any differences between men and women in the distribution of migrants by age and educational level (see Figure S26b). However, we noticed that although the distribution of migrants by age and educational level is quite similar

between the two emigration definitions for 2001-11, at younger ages the low educated tend to report their emigration less, and the high educated at younger ages tend to report their emigration more (see Figure S26c). We corrected for this difference in reporting between the high and the low educated by multiplying the total population distribution by age and educational level of the migrants based on "embarkations" in 2011-2017, with age- and education-specific ratios of emigrants according to the two different emigration definitions in 2001-2011. These ratios were calculated – based on data for 2001-2011 as the age- and education-specific number of emigrants based on absence in the subsequent census divided by the age- and education-specific number of emigrants based on embarkations. This resulted in ratios higher than 1 for the low educated at young ages (in line with the underreporting), and in low ratios for the high educated at young ages (in line with their overreporting). In applying these ratios we made sure that the adjusted distribution again adds to one.

As a third step we obtained the adjusted age-, sex- and education-specific estimates of personyears for 2011-2017, thereby taking into account that in 2011 the overestimation is less severe because it only applies as of March 28. We do so by subtracting the age-, sex- and education-specific estimates of the overestimated personyears (i.e. the number of "missed" emigrants) from the baseline age-, sex- and education-specific personyears for each year from 2012-2017. We did this for each year because in our study we deleted emigrants from the complete follow-up period (given the lack of information about the exact date of emigration), so the overestimation of personyears counts equally for the different years. For 2011, however, we need to take into account that the underestimation of migrants only started from March 28 onwards (= after 31 + 28 +27 days). So the overestimation of personyears in 2011 only counts for (365 – (31+28+27) /365) = 0.764384.

As a final step, we estimated the age-, sex-, year-, and education-specific overestimated number of deaths and deducted these from the baseline death numbers. We estimated the age-, sex-, year-, and education-specific overestimated death numbers by applying the age-, sex-, year- and education-specific death rates to the overestimated age-, sex-, year-, and education-specific personyears. This approach implies that the "missed" emigrants are assumed to have the same risk of dying compared to their counterparts in the same strata who did not emigrate. Although this is a tricky assumption it is the only assumption one can make to adjust the deaths for the "missed" emigrants in line with the personyears for the "missed" emigrants, beyond simply not adjusting the deaths. Furthermore, especially younger people emigrate, so the effect on mortality is expected to be rather limited. Additionally, any potential bias because of this assumption will be partly reversed in the next step where the age-specific mortality rates of ONS-LS are aligned to those observed for the national population.

Our adjustment for the inconsistent emigration definition before and after 2011 resulted in a decrease in total personyears of 185,085 in the 2011-2017 period (95,913 for men and 89,170 for women) (Table S14), which resolved part of the trend break in the sex-specific personyears in 2011 (Figure S27). The jumps in 2011 in the trends in the number of adjusted personyears by educational level are now more in line with what can be expected in terms of educational expansion in E&W (Greenaway & Haynes 2003; Paterson 2022b) and are also more in line with the jumps in 2001. In terms of the share of personyears by educational level hardly any effect is visible.

Figure S28 illustrates that the effect of our adjustment on the deaths is small. This is to be expected, because migration particularly occurs at younger ages when mortality is relatively low. An effect on the number of deaths by educational level is hardly visible, and the share of deaths by educational level has remained largely unchanged. Regarding the CDR we can see a small effect (Figure S29). The SDRs remain unaltered, because we applied the age-specific death rates to the overestimated personyears.

Figure S26. Comparison of the population distribution by age and educational level of emigrants according to different emigration definitions for the 2001-2011 and 2011-2017 follow-ups, for individuals aged 20 and older, by sex, England and Wales

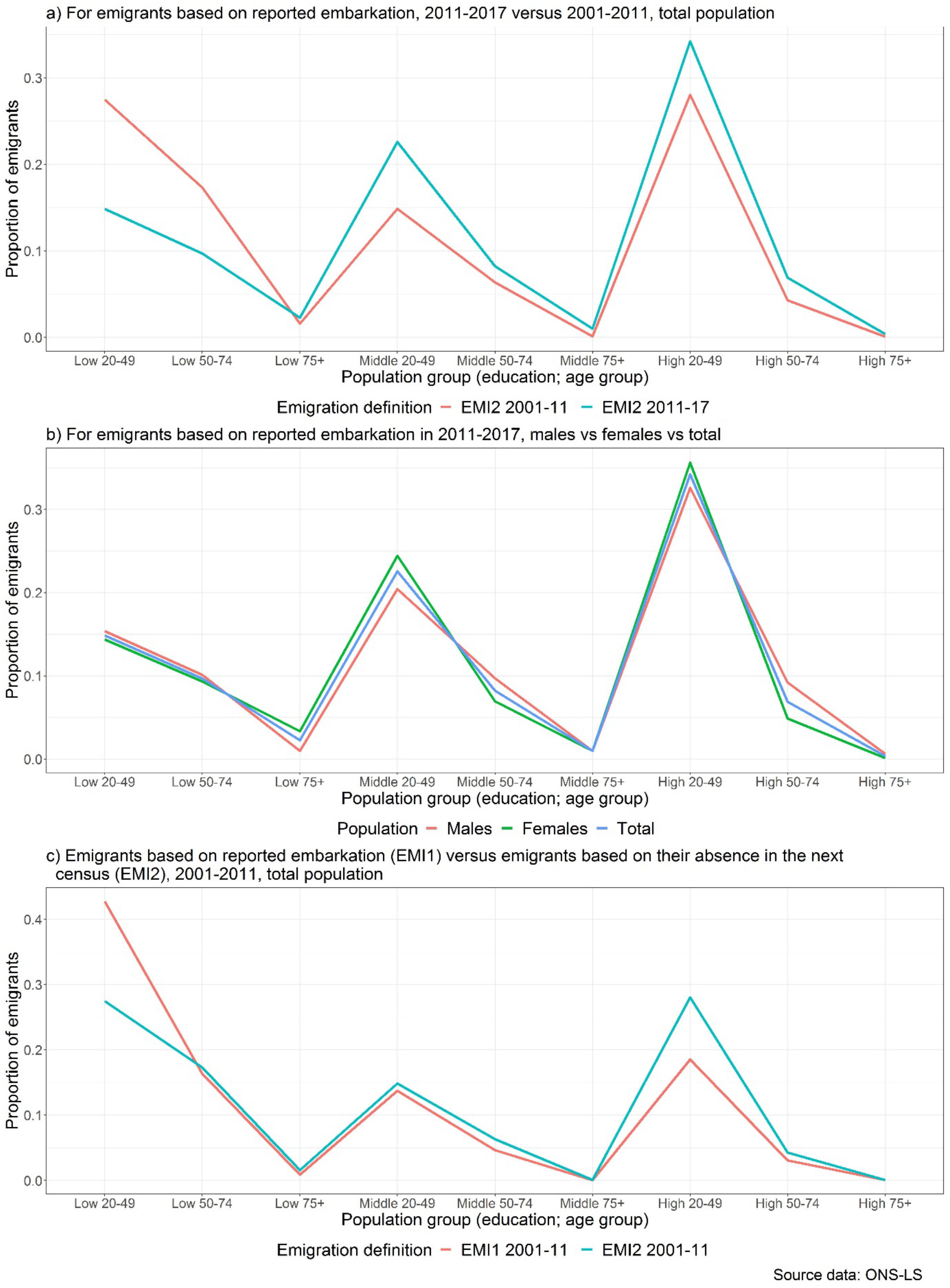

Figure S27. Trends in the number of personyears, the number of personyears by educational attainment group, and the share of personyears by educational attainment group (%), before and after adjusting for the inconsistent emigration definition from 2011 onwards, for individuals aged 30 and older, by sex, England and Wales, 1972-2017

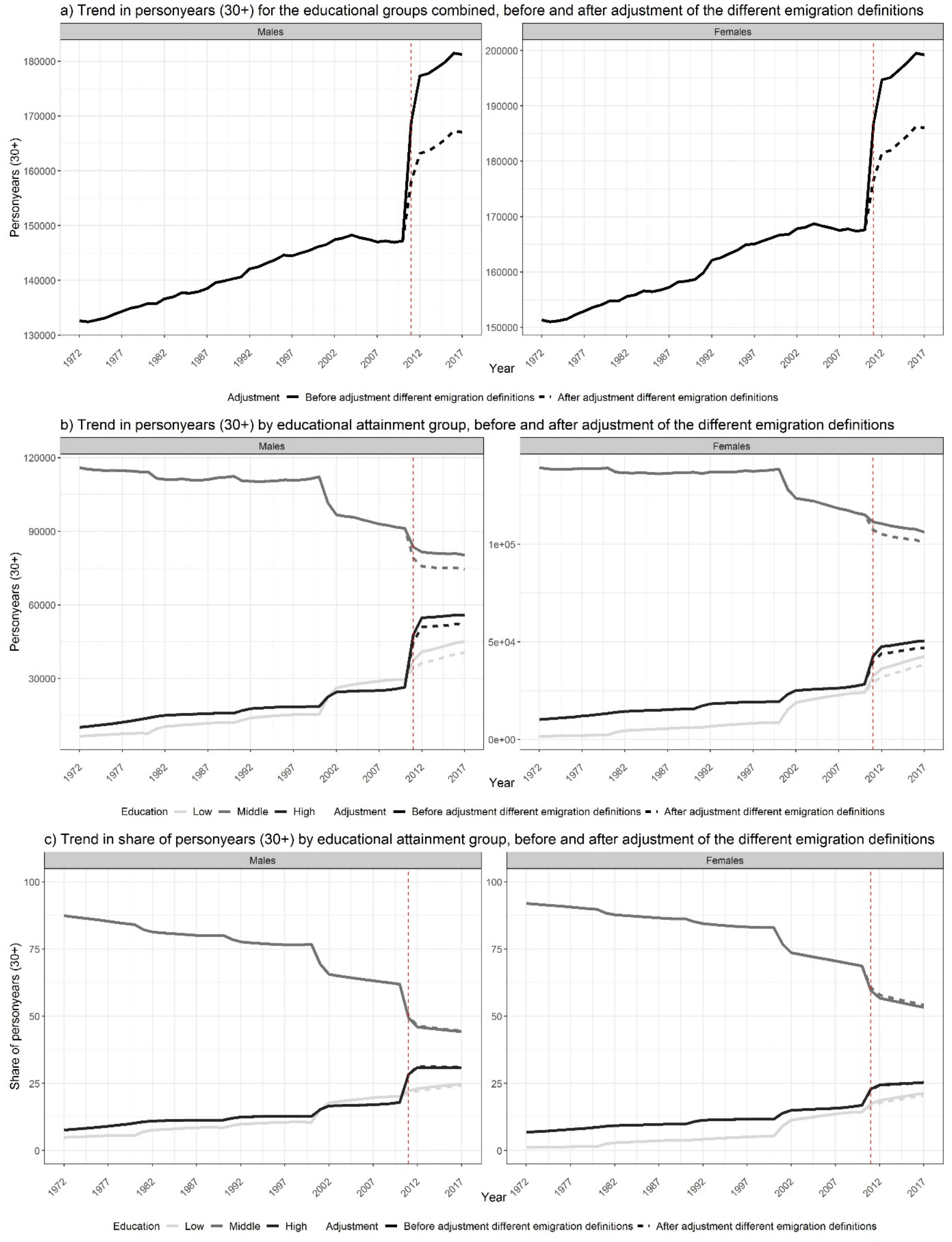

Source data: ONS-LS

Figure S28. Trends in the number of deaths, the number of deaths by educational attainment group, and the share of deaths by educational attainment group (%), before and after adjusting for the inconsistent emigration definition from 2011 onwards, for individuals aged 30 and older, by sex, England and Wales, 1972-2017

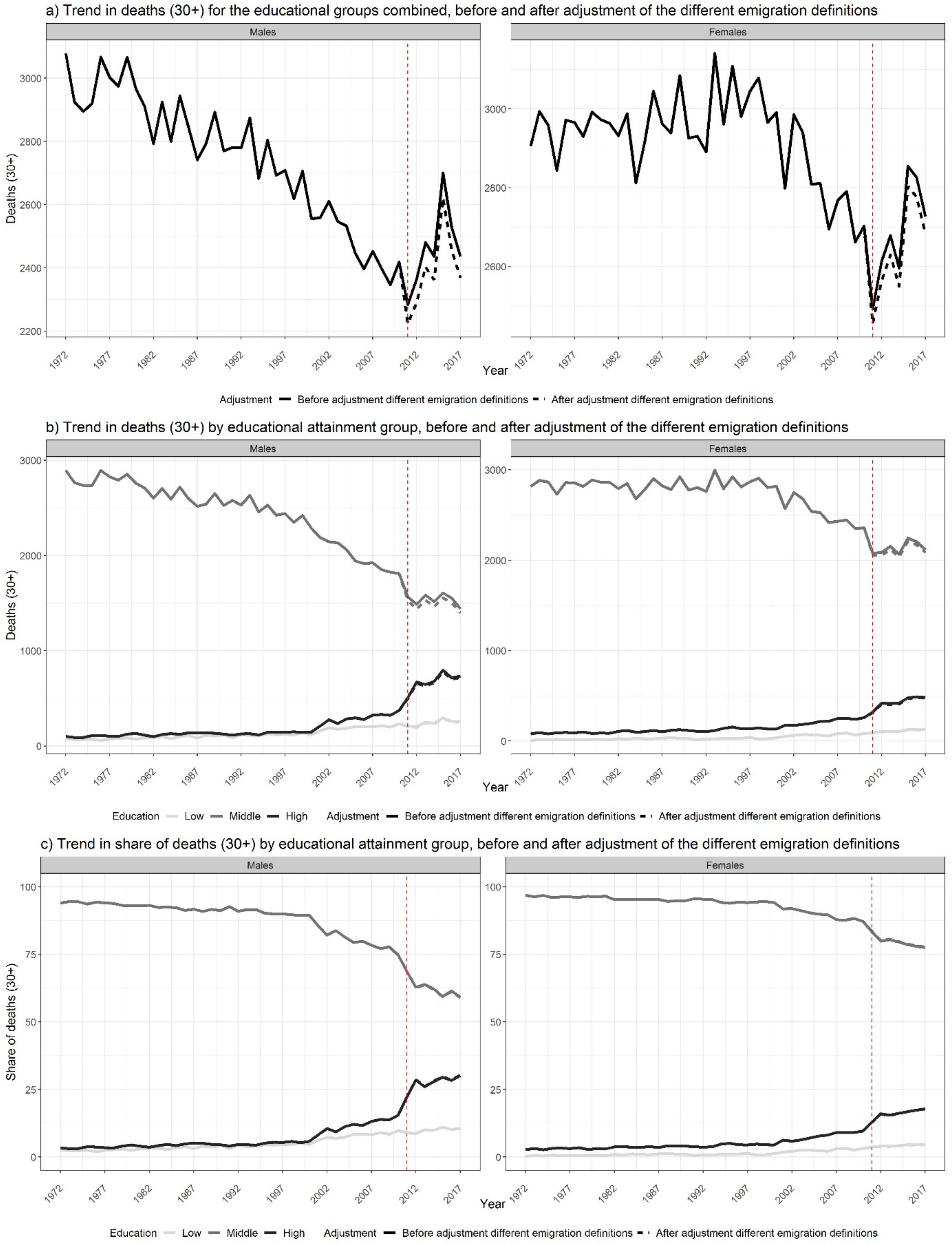

Source data: ONS-LS

Figure S29. Trends in the crude death rate (CDR) by educational attainment group before and after adjusting for the inconsistent emigration definition from 2011 onwards, for broad age groups (30+, 30-74, 75+), by sex, England and Wales, 1972-2017

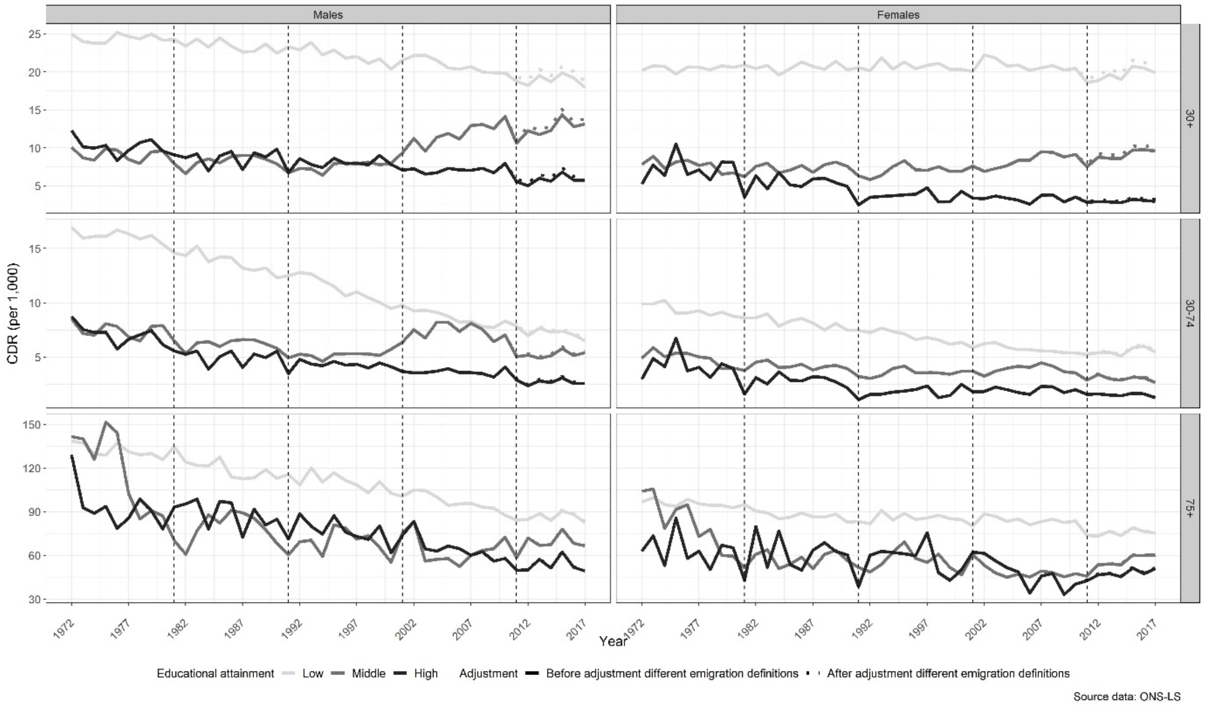

5.6 Alignment of our ONS-LS data selection with administrative country-level data

As a final step of the adjustment, we adjust for the imperfect and time-varying alignment of the population and mortality data for the three educational groups combined in our ONS-LS data selection with country-level administrative population and mortality data for England and Wales (i.e. the HMD data). To recap, especially between 1990 and 2010 we observed – in particular - higher CDR (and higher SDR for women) for the total ONS-LS population aged 30 and older compared to the country-level data. This is mainly because the increases in the number of personyears in our ONS-LS data selection have not been in line with increase in the size of the national population of E&W, based on administrative data. These inconsistencies are still visible after the previous two data adjustments (Figure S30). Figure S31 shows the current representativeness of the data.

To adjust for the imperfect and time-varying alignment of our ONS-LS data selection, we assumed similar age-, sex-, and year-specific death rates for the total ONS-LS population (= the three educational groups combined) and the national population, thereby maintaining the differences between educational groups. For this purpose we matched the age-, sex-, and year-specific deaths and personyears for the three educational groups combined in the ONS-LS with the respective administrative numbers divided by 100, in line with the ONS-LS being a 1% sample of the population of E&W. That is, either purely adjusting the deaths or purely adjusting the personyears to achieve similar death rates would require additional assumptions that could not be justified.

To maintain the education-specific differences observed in our adjusted ONS-LS data selection, we applied the existing age-, sex-, and year-specific shares of deaths by educational level to the adjusted age-, sex- and year-specific death numbers for the three educational groups combined, and – similarly – we applied the existing age-, sex-, and year-specific shares of personyears by educational level to the adjusted age-, sex- and year-specific personyears for the three educational groups combined.

Figure S30. Trends in the number of personyears, number of deaths, the crude death rate (CDR) and age-standardised mortality (SDR) compared between our ONS-LS data selection and national data from the Human Mortality Database (HMD, 2021), before the alignment with national data, for individuals aged 30 and older, by sex, England and Wales, 1972-2017

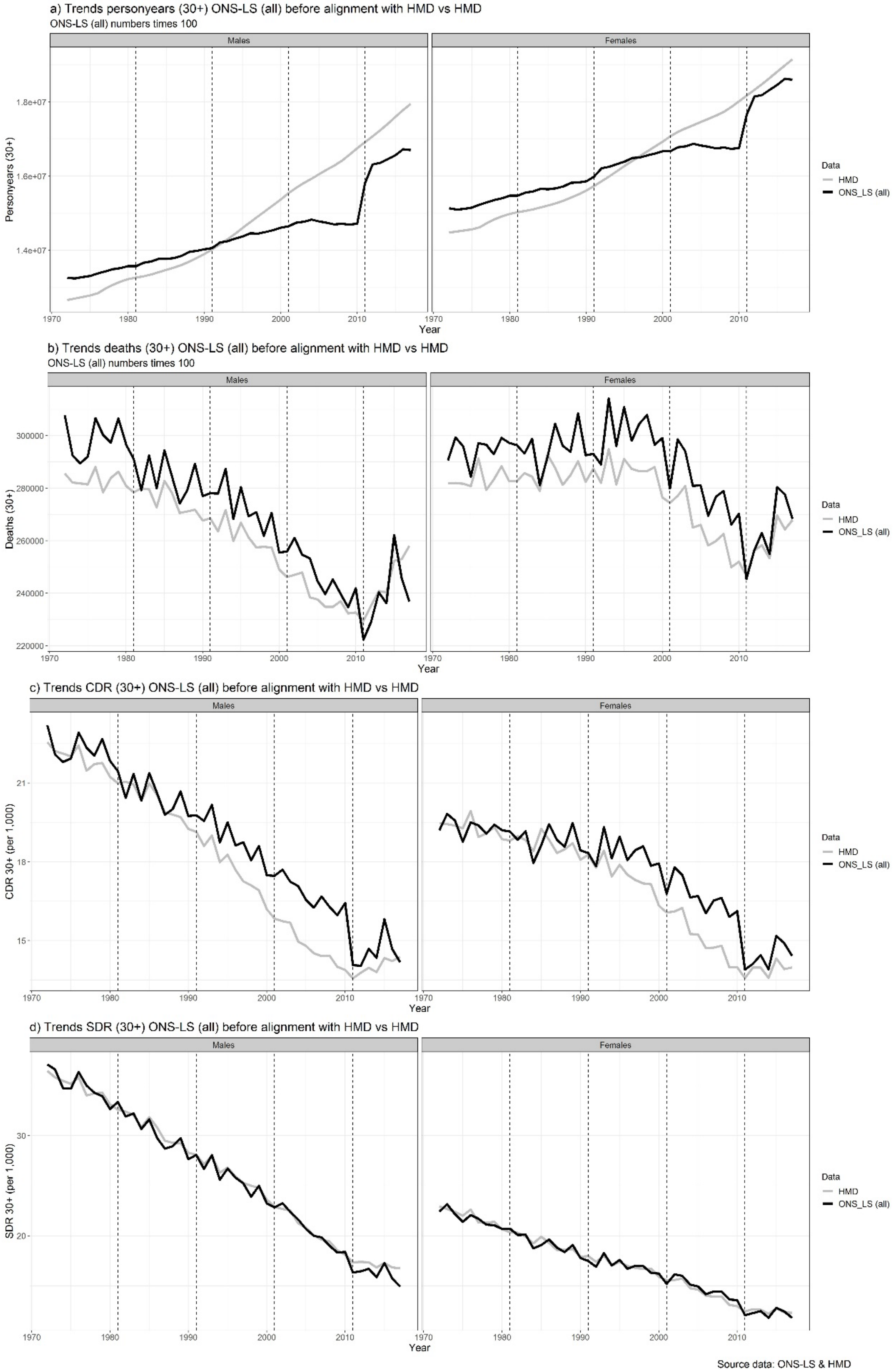

Figure S31. Trends in the representativeness of the deaths and personyears in our ONS-LS data selection for the national population, for individuals aged 30 and older, by sex, England and Wales, 1972-2017

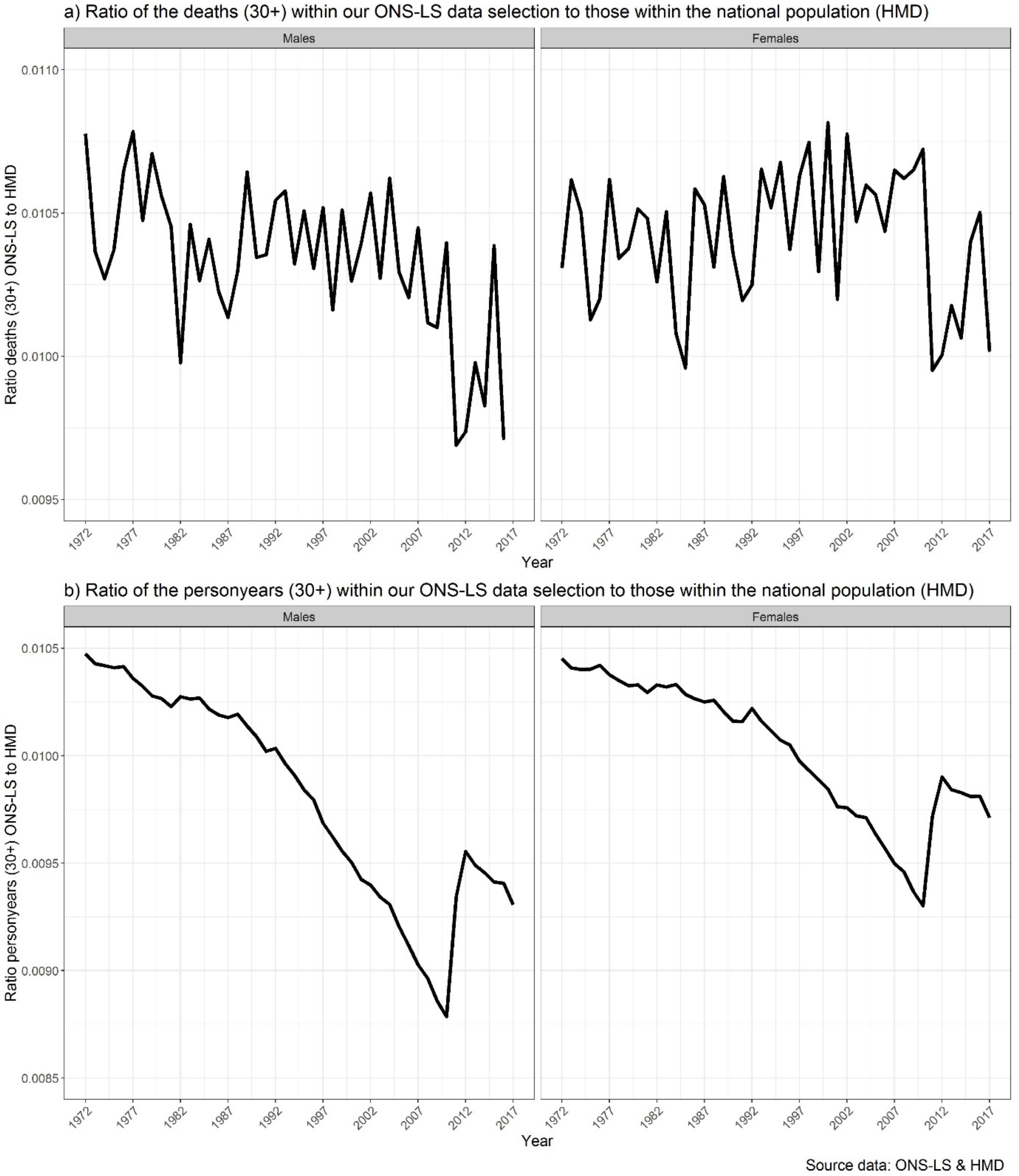

The effects of our alignment exercise is that, for the three educational groups combined, the age-specific deaths, age-specific personyears, age-specific mortality rates, and consequently CDR and SDR for the adjusted ONS-LS data are now in complete alignment with the national data from the HMD. Consequently, in line with the HMD data, we also no longer observe trend breaks in CDR and SDR for the total population (the three educational groups combined) in the adjusted ONS-LS data in 2011 (Figure S30).

For the different educational levels, we observe that the age pattern of deaths and personyears before and after the alignment exercise with the national data from HMD does not radically differ (Figures S32-S33). The alignment of the deaths and personyears with the national data resulted however in slightly elevated deaths and personyears at younger ages from 1991 onwards (Figures S32-S33). After alignment with the national data from HMD, the age-specific mortality rates fluctuate less and are more in line with the generally observed exponential increase in mortality rates with increasing age after age 30 (Gompertz, 1825)(Figure S34).

In terms of the CDR by educational level (Figure S35), we find that the alignment with HMD results in lower CDRs in the 1992-2011 period compared to before for both sexes. This is because, particularly in 1992-2011, the increase in the personyears for the overall ONS-LS population was not in par with the increase in personyears for the national population. The latter can be attributed to the ONS-LS sample size staying the same over this time period, and because we excluded emigrants, whose numbers were particularly large at that time. The effect in absolute terms is highest for the low educated because their numbers are higher compared to the middle and high educated.

In terms of the SDR by educational level (Figure S36), we hardly see an effect of the alignment with HMD for women. For men, however, the SDR levels become larger from 2011 onwards compared to before for all three educational groups. This is because, indeed, for men we saw – before the adjustment - lower SDR for the ONS-LS total population compared to the national population (HMD) (Figure S30).

Figure S32. Relative age pattern of deaths before and after the alignment with national data, by sex and educational group, England and Wales, selected years. *Age groups: 30 = 30-34, … , 90 = 90-94, 95 = 95+.*

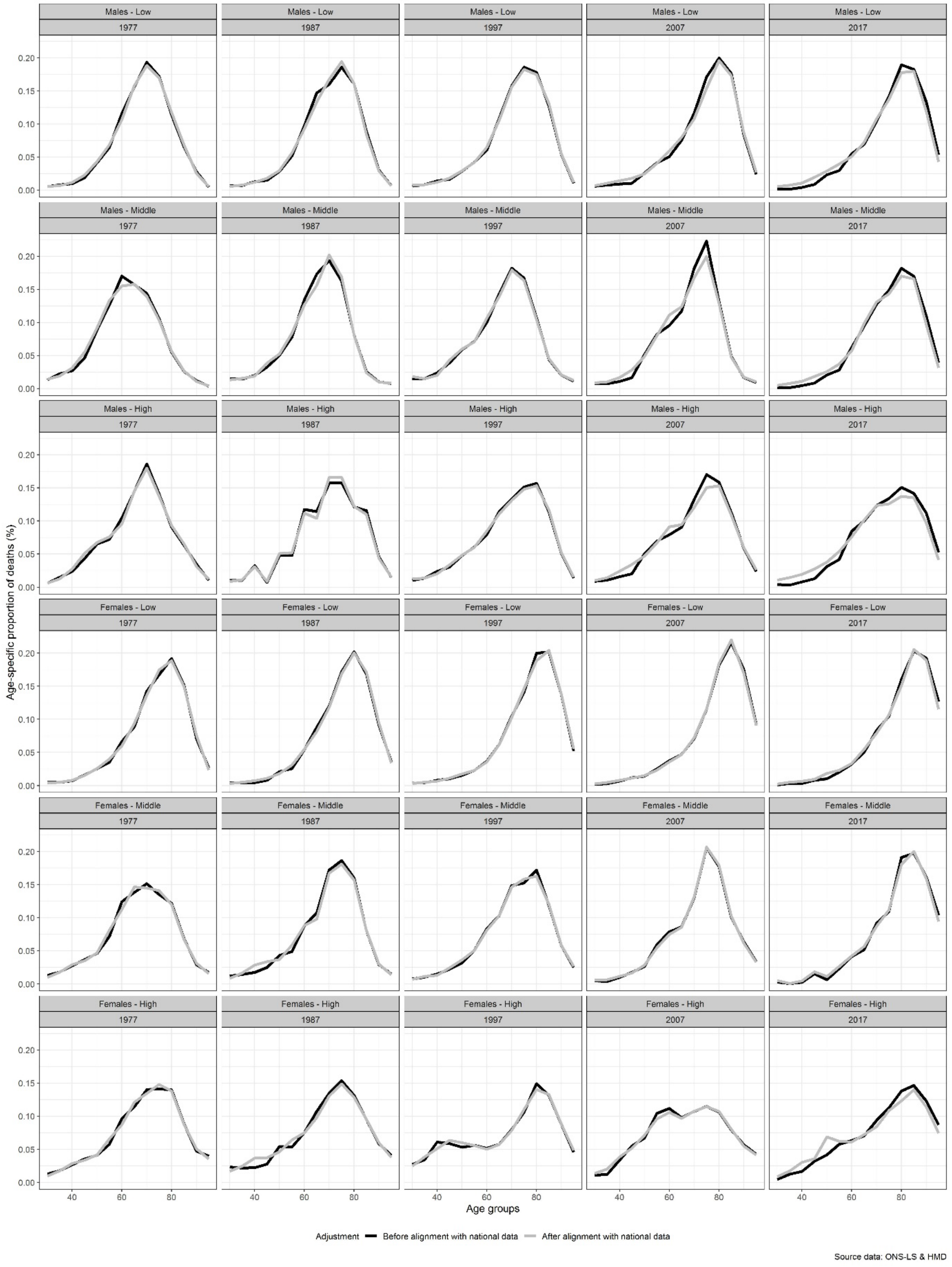

Figure S33. Relative age pattern of personyears before and after the alignment with national data, by sex and educational group, England and Wales, selected years. *Age groups: 30 = 30-34, … , 90 = 90-94, 95 = 95+.*

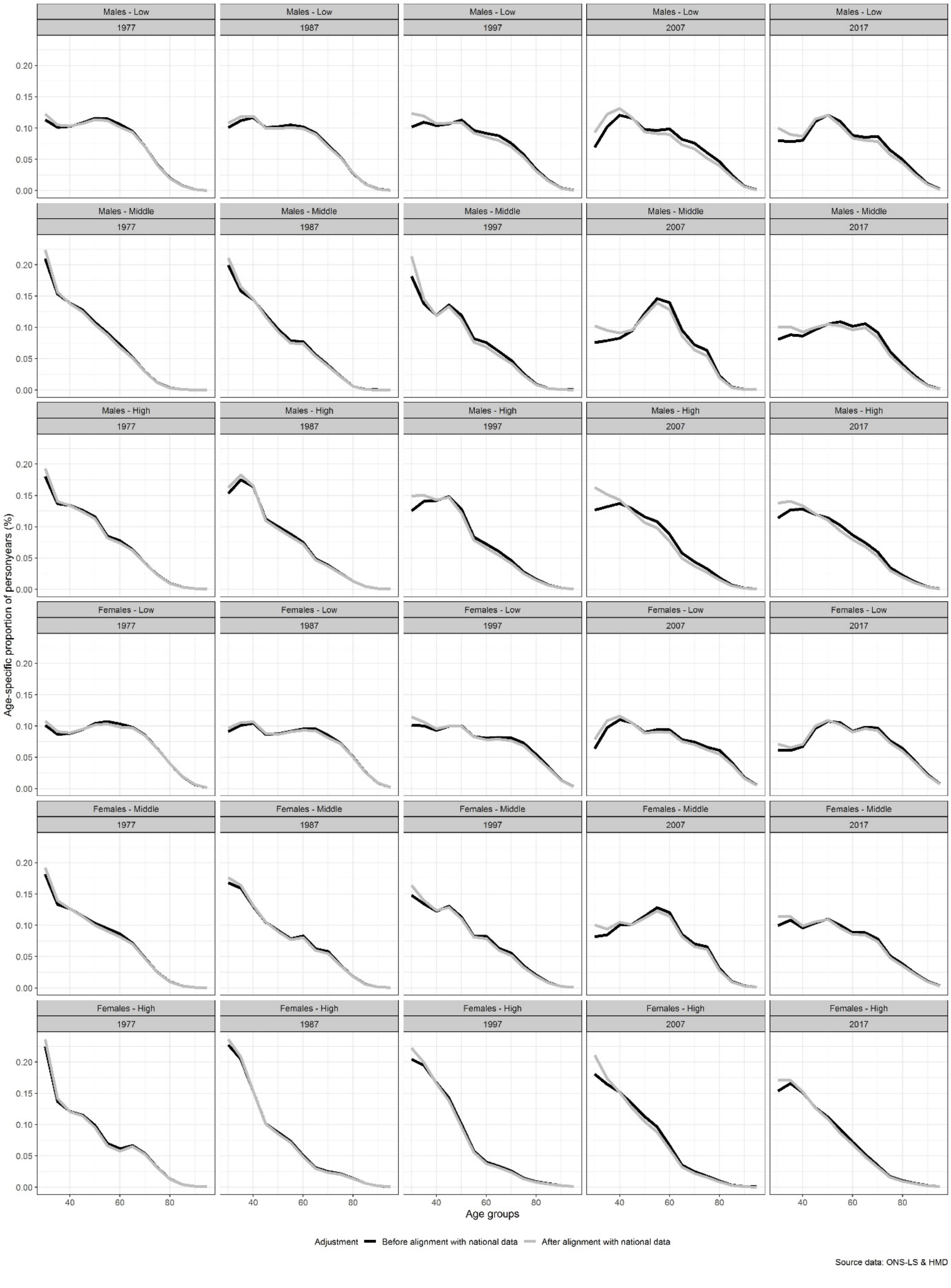

Figure S34. Age-specific mortality rates (log scale) before and after the alignment with national data, by sex and educational group, England and Wales, selected years. *Age groups: 30 = 30-34, … , 90 = 90-94, 95 = 95+.*

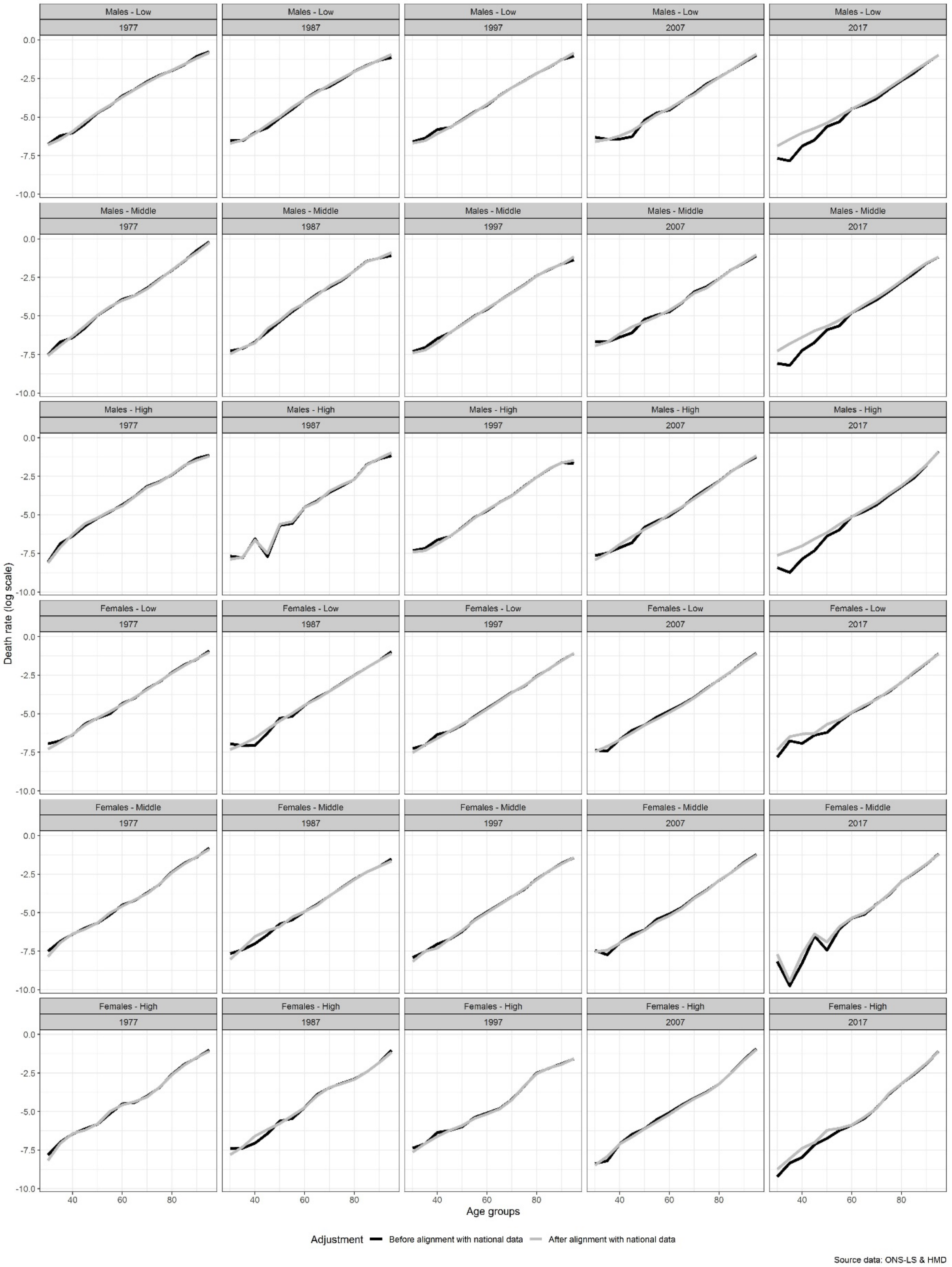

Figure S35. Trends in the crude death rate (CDR) by educational attainment group before and after alignment with national data, for broad age groups (30+, 30-74, 75+), by sex, England and Wales, 1972-2017

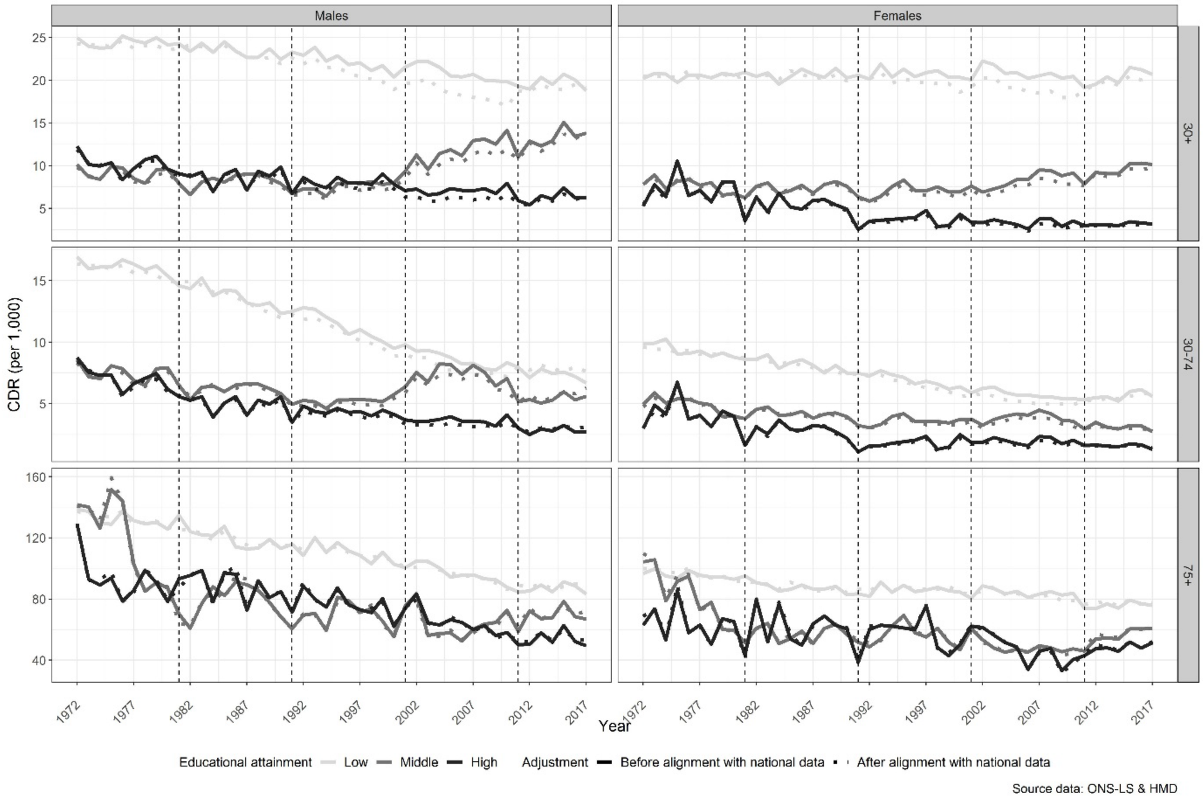

Figure S36. Trends in age-standardised mortality (SDR) by educational attainment group before and after alignment with administrative country-level data, for broad age groups (30+, 30-74, 75+), by sex, England and Wales, 1972-2017

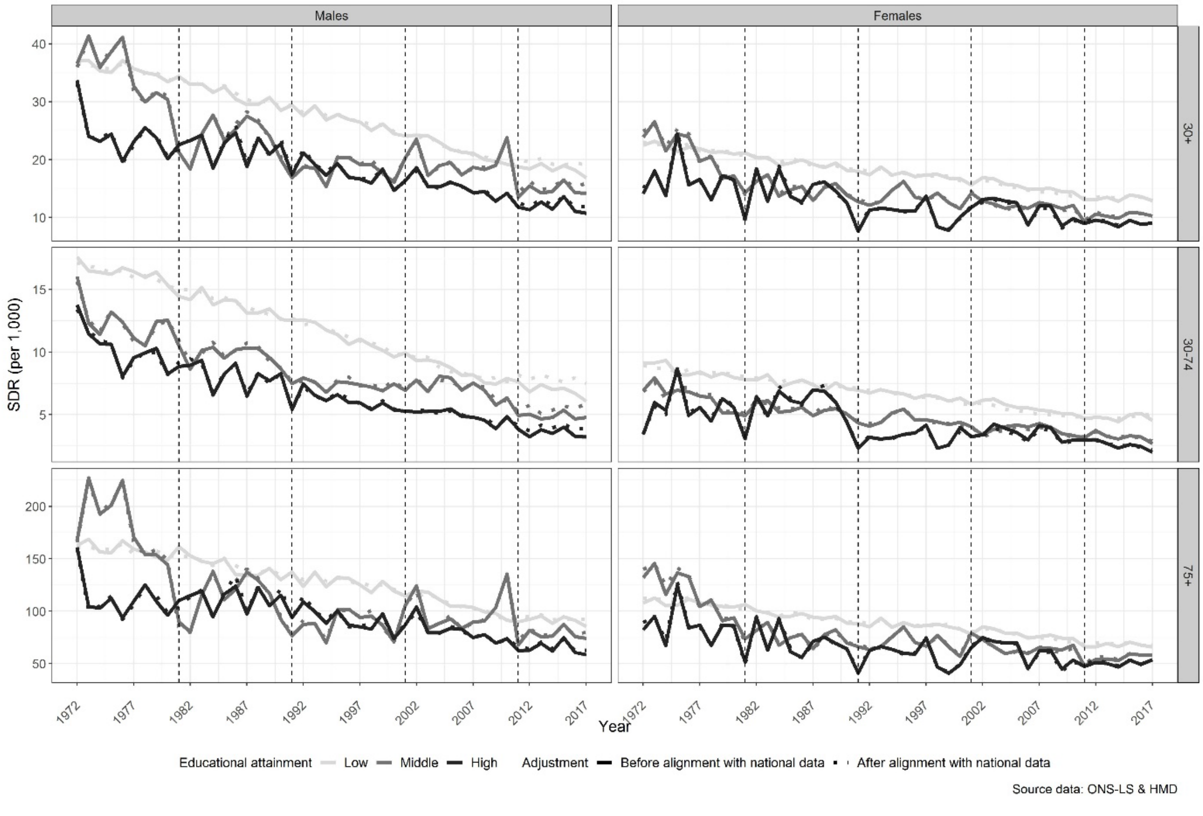

6. SYNTHESIS

6.1 Appraisal of the assumptions

To facilitate the adjustment of the data, it was necessary to make several assumptions, which we did after carefully evaluating different alternatives.
First, in optimising the available education information at the individual level, we assumed that those with other educational qualifications in 2001 and 2011 could be considered middle educated. This decision was based on the variable description, and supported by the results from a sensitivity analysis in which we compared the mortality levels and trends of those with other educational qualification with the low, middle and high educated group.
Second, we dealt with missing educational information as far as possible at the individual level, and performed additional necessary adjustments at the aggregate level, thereby assuming that those with missing education were missing at random. This might not be true given potential differences in underreporting (e.g. those aged 70+ in 1971 were only asked to complete the education question if they had been employed in the previous week) and a potential different population distribution for those aged 75+ in 2001 for whom we could not use the educational information from 1991 compared to those for whom we could. However, the same assumption is implicitly made in previous research that omitted those with missing educational information. We choose to implement this assumption directly into the data to obtain larger numbers, and to obtain complete period data for those aged 30 and over from 1972 onwards. Since this assumption particularly applies to the elderly, we recommend treating the results for those aged 70+ in 1972-1981 and – to a lesser extent - those aged 75+ in 2002-2011 with some caution.
Third, we assumed no sudden jumps in the (age-specific) trends in the share of personyears by educational level except those that could be the effect of educational expansion. In adjusting for the trend break in 1981 we assumed no upward jump in the share of personyears for the middle educated between 1980 and 1982 due to educational expansion. No jump due to educational expansion was safer to assume for the middle than the low educated, and safer to assume than trying to estimate its possible effect. We further assumed that for the personyears that were redistributed from the low to the middle educated the smoothed age-specific death rates of the middle educated applied. Given that for the share of deaths for the middle educated we can – for some age groups – still discern a small drop from 1980 to 1982, we believe that – if anything – our adjustment for the trend break in 1981 is rather conservative.
Fourth, in adjusting for the inconsistent emigration definition we assumed that the number of 'missed' emigrants can be estimated - separately for males and females - by the difference in personyears between 2012 and 2010 minus the difference in sample size between the 2011 and 2001 follow-up. We also assumed that these 'missed' emigrants have the same distribution – by age and educational level – as the emigrants based on "embarkations" in 2011-2017, after correcting for the difference in reporting between the high and the low educated. Moreover we assumed that the "missed" emigrants have the same risk of dying compared to their counterparts in the same strata who did not emigrate. Although the risk of dying of emigrants likely differs from non-emigrants in the same socio-economic strata, it is mostly younger individuals with low mortality risk who emigrate. Therefore, this assumption is unlikely to have a significant effect on mortality. Additionally, any potential bias due to this assumption is partly reversed in the final adjustment step where the age-specific mortality rates of ONS-LS are aligned to those observed for the national population.
Finally, we assumed the deaths and personyears by year, sex, and age in our ONS-LS selection to be equal to the respective deaths and personyears for the national England & Wales population, divided by 100, while maintaining the education-specific differences observed in our ONS-LS data selection. Thus essentially we assumed, and corrected for, a change in representativeness of the ONS-LS data selection while maintaining underlying education-specific differences in deaths and personyears.

6.2 After all adjustments

Our optimisation of the individual data regarding education level resulted in important changes in the educational distribution of ONS-LS members aged 20+ at the different censuses compared to the use of the main ONS-LS education variables (Table S6). First, the 352,355 LS-members with “missing or not applicable” in the summary education variable in 1991, were redistributed into 350,658 without qualifications (i.e., low educated), and 1,697 with truly missing educational information (i.e., missing). Second, respondents in 2001 and 2011 with professional qualifications but without a degree (11,125 in 2001 aged 20-74; 31,524 in 2011 aged 20+) were classified as middle instead of high educated. Third, 5,900 LS members with “other professional qualifications” in 2001 were moved from the “other” to the middle educated category, so that the “other” category in 2001 more closely resembled that in 2011. Finally, we treated the “other” educated (21,356 in 2010; 29,002 in 2011) as middle educated. In addition, of the 41,960 LS members aged 75 and older in 2001, who all had missing educational information, 91.6% were assigned to either the low (35,564), middle (1,557), or high (1,297) educated category based on their education information in 1991 (Table S7).

Figures S37-S40 show the final trends over time – by educational level - in the share of personyears, the share of deaths, the CDR and the SDR. No unrealistic trend breaks remain after the application of our different adjustment steps. Figure S41 illustrates that age-specific death rates by educational level have changed quite substantially as a result of our data adjustment at the aggregate level.

At the aggregate level, the largest effect of our adjustment proved to occur among the low educated (Figure S41; Table S14). First, those with missing educational information (which occurred especially among individuals aged 70 and over) were predominantly assigned to the low educated, in line with their larger shares in the population. Second, they were also affected by the misclassification of individuals with upper-level secondary education qualification to low educated instead of middle educated in the 1981 and 1991 censuses, resulting in slightly lowered mortality levels. Third, they experienced the largest emigration numbers and therefore also the highest underestimation of migrants from 2011 to 2017 due to their lower reporting rates in the NHSCR. Fourth, the alignment with country-level data had the largest effect on them because – again – they exhibit higher shares in terms of both personyears and deaths. They were, however, not affected by the smoothing over age that we employed.

The data for the high educated were predominantly affected by the smoothing over age because their death numbers were lowest. In addition, the alignment with the national data had a small effect for high educated men in the more recent years. The data for the middle educated were affected more than for the high educated, but less than for the low educated. That is, the smoothing over age affected them quite a bit, particularly in the 1981-2001 period. Due to the misclassification of individuals with upper-level secondary education qualification to low educated instead of middle educated in the 1981 and 1991 censuses, they acquired larger numbers after our adjustment, but age-specific death rates and consequently SDR remained largely the same. The effect of emigration and the alignment with the national data fell in between the effect for the low and the high educated. The larger effects of our adjustment at the aggregate level for the low compared to high educated – particularly from 1981 onwards - will likely impact (trends in) educational inequalities in mortality.

The overall outcome of our adjustment is the obtainment of time-consistent aggregate mortality data based on the internationally comparable categorization of highest educational attainment into the low, middle, and high educated. Based on these adjusted data, we could study the long-term trends in educational inequalities in mortality in E&W by means of established methods (see the main manuscript).

Figure S37. Trends in the share of personyears by educational attainment group (%) after all adjustments, for broad age groups (30+, 30-74, 75+), by sex, England and Wales, 1972-2017

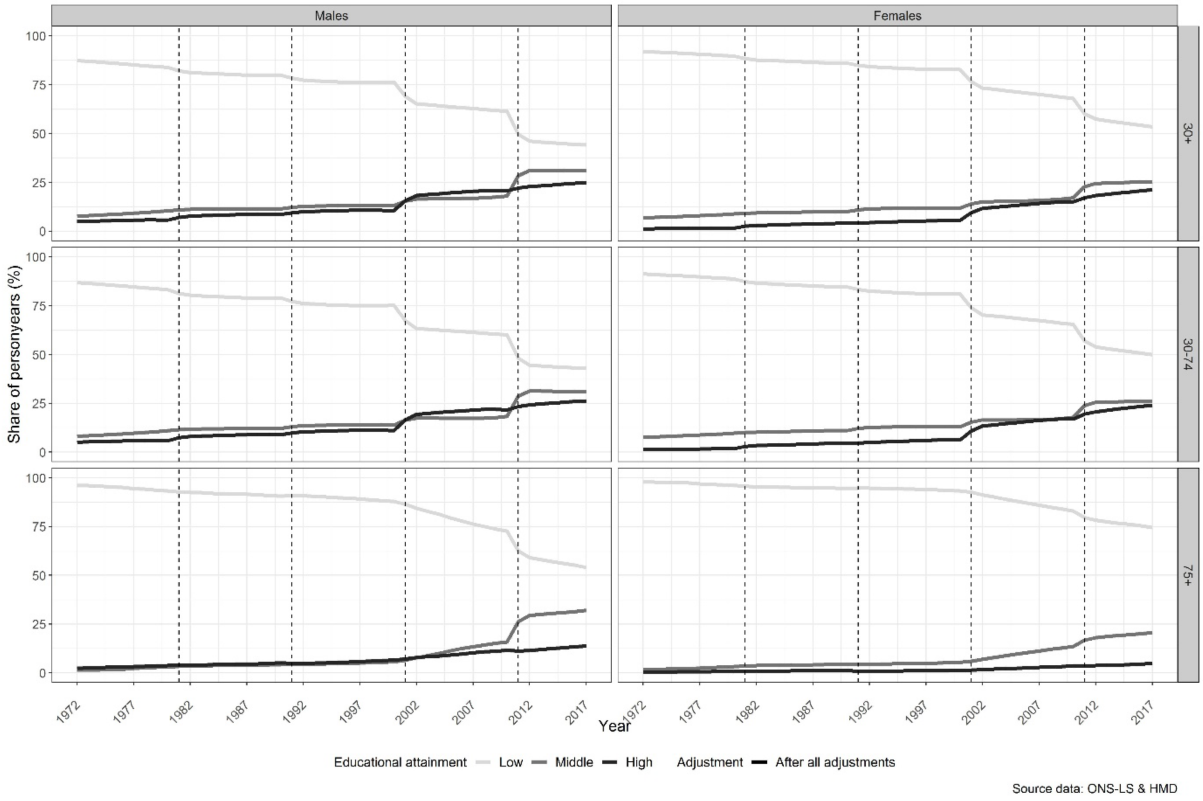

Figure S38. Trends in the share of deaths by educational attainment group (%) after all adjustments, for broad age groups (30+, 30-74, 75+), by sex, England and Wales, 1972-2017

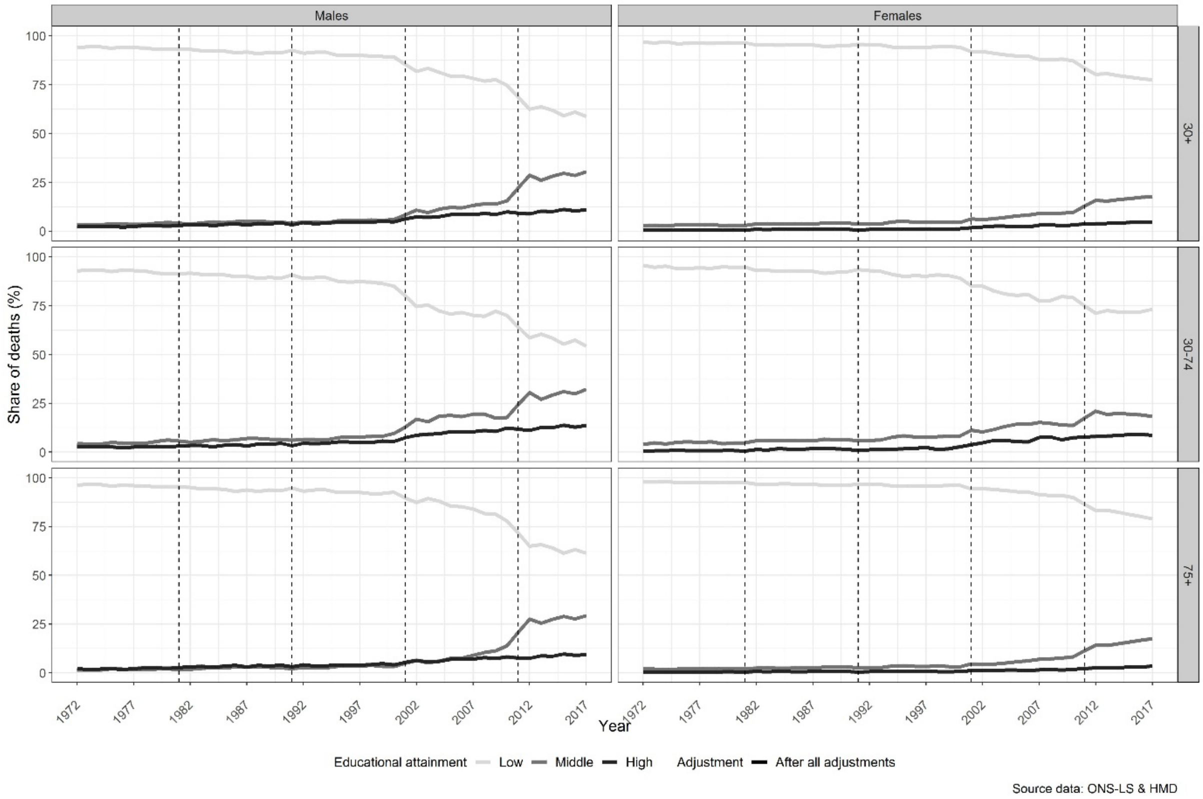

Figure S39. Trends in the crude death rate (CDR) by educational attainment group after all adjustments, for broad age groups (30+, 30-74, 75+), by sex, England and Wales, 1972-2017

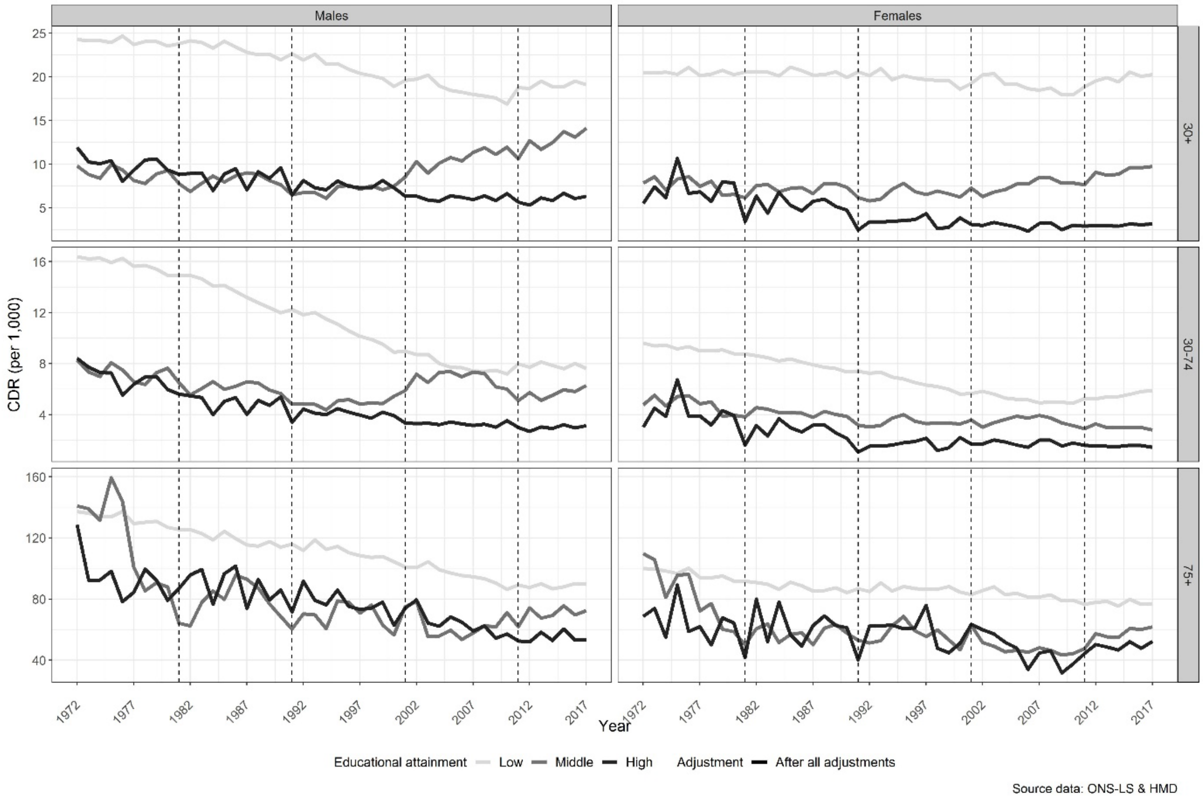

Figure S40. Trends in the age-standardised mortality (SDR) by educational attainment group after all adjustments, for broad age groups (30+, 30-74, 75+), by sex, England and Wales, 1972-2017

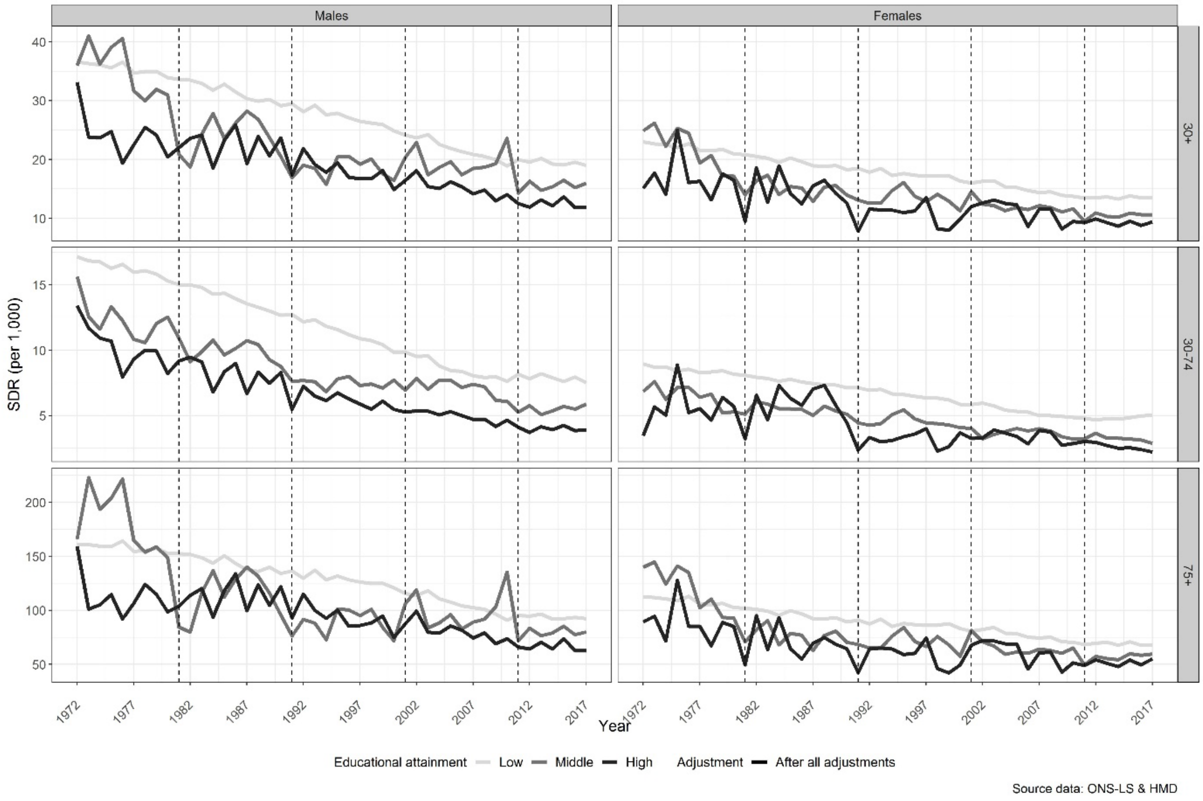

Figure S41. Age-specific mortality rates (log scale) before and after all adjustments, by sex and educational group, England and Wales, selected years

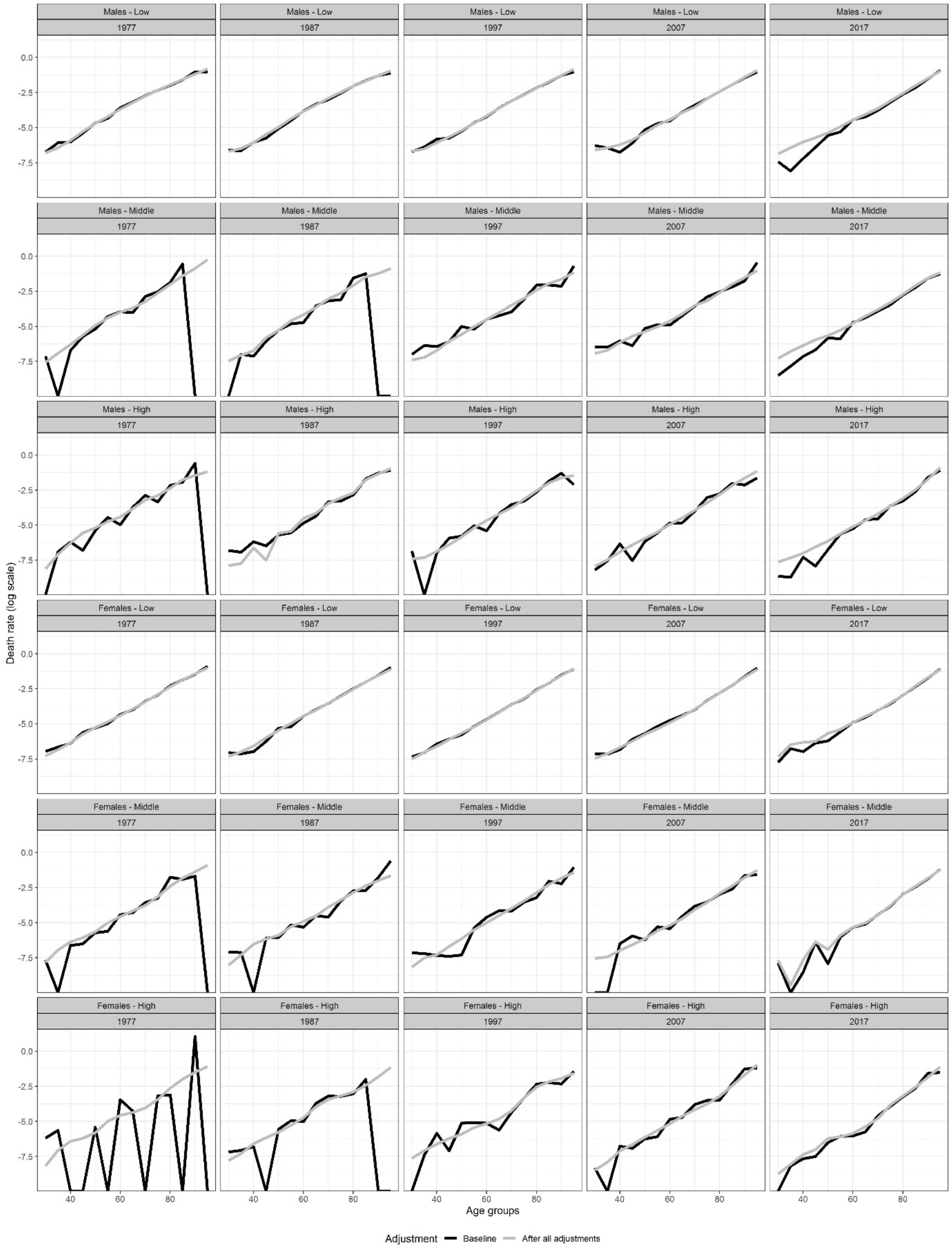

Source data: ONS-LS & HMD

Table S14 – Number of personyears (PY), number of deaths, and age-standardised mortality (SDR) at baseline and after the different adjustments, for individuals aged 30 and older, by educational attainment group, sex and period, England and Wales, 1972-2017

Period	Educational attainment								
	Low (ISCED1997 0-2)			Middle (ISCED1997 3-4)			High (ISCED1997 5-6)		
	PY	Deaths	SDR	PY	Deaths	SDR	PY	Deaths	SDR
**Men - 1972-1981**									
Baseline	1101785	24267	34.90	111375	950	30.28	72132	662	22.95
Smoothing	1101785	24267	34.90	111375	950	33.23	72132	662	23.59
Redistribution missings	1151582	28016	35.49	114591	1038	33.95	74543	751	24.01
Trend break 1981	1145119	27964	35.52	121054	1090	33.96	74543	751	24.01
Emigration	1145119	27964	35.52	121054	1090	33.96	74543	751	24.01
Alignment national data	1103431	26520	35.34	118306	1038	33.84	72489	715	23.93
**Men - 1982-1991**									
Baseline	1200748	26721	30.59	68879	560	23.52	115813	986	22.03
Smoothing	1200748	26721	30.62	68879	560	22.81	115813	986	21.72
Redistribution missings	1201210	26739	30.63	68906	560	22.82	115859	987	21.72
Trend break 1981	1113259	26023	30.85	156856	1276	23.38	115859	987	21.72
Emigration	1113259	26023	30.85	156856	1276	23.38	115859	987	21.72
Alignment national data	1090487	25226	31.16	155857	1247	23.68	114838	961	22.03
**Men - 1992-2001**									
Baseline	1178852	24674	26.21	102598	908	18.51	155895	1241	17.75
Smoothing	1178852	24674	26.24	102598	908	18.63	155895	1241	17.57
Redistribution missings	1184175	24820	26.25	103022	914	18.65	156581	1249	17.59
Trend break 1981	1099871	24264	26.48	187326	1470	18.55	156581	1249	17.59
Emigration	1099871	24264	26.48	187326	1470	18.55	156581	1249	17.59
Alignment national data	1124091	23277	26.76	196692	1431	18.78	163425	1206	17.80
**Men - 2002-2011**									
Baseline	926634	18685	21.20	271427	3034	17.71	291273	1972	14.80
Smoothing	926634	18685	21.20	271427	3034	18.94	291273	1972	14.75
Redistribution missings	931972	19342	21.28	272035	3073	19.02	292076	2021	14.81
Trend break 1981	929117	19178	21.31	274890	3237	18.97	292076	2021	14.81
Emigration	924655	19137	21.31	271974	3225	18.97	288615	2014	14.81
Alignment national data	1005417	18557	21.38	294947	3177	19.03	326107	1989	14.85
**Men – 2012-2017**									
Baseline	485558	9188	18.29	332230	4247	14.87	258225	1509	11.73
Smoothing	485558	9188	18.29	332230	4247	14.85	258225	1509	11.79
Redistribution missings	485771	9191	18.29	332381	4248	14.85	258378	1509	11.79
Trend break 1981	485771	9191	18.29	332381	4248	14.85	258378	1509	11.79
Emigration	450747	8892	18.29	309495	4150	14.85	231213	1461	11.79
Alignment national data	473536	9044	19.42	326191	4233	15.66	250953	1524	12.41

Table S14 - continued

Period	Educational attainment								
	Low (ISCED 0-2)			Middle (ISCED 3-4)			High (ISCED 5-6)		
	PY	Deaths	SDR	PY	Deaths	SDR	PY	Deaths	SDR
**Women - 1972-1981**									
Baseline	1300075	22506	21.25	110047	726	20.04	21675	125	15.95
Smoothing	1300075	22506	21.25	110047	726	20.07	21675	125	15.41
Redistribution missings	1390678	28488	21.81	114483	862	20.77	22550	150	15.88
Trend break 1981	1385311	28448	21.81	119850	902	20.87	22550	150	15.88
Emigration	1385311	28448	21.81	119850	902	20.87	22550	150	15.88
Alignment national data	1333816	27334	21.93	116536	865	21.14	22031	145	16.08
**Women - 1982-1991**									
Baseline	1437686	28622	19.11	80376	619	15.14	55353	288	14.75
Smoothing	1437686	28622	19.13	80376	619	14.75	55353	288	14.24
Redistribution missings	1437984	28631	19.13	80396	619	14.75	55367	288	14.24
Trend break 1981	1364806	28120	19.23	153574	1130	14.79	55367	288	14.24
Emigration	1364806	28120	19.23	153574	1130	14.79	55367	288	14.24
Alignment national data	1328374	27191	19.35	151121	1093	14.91	54994	280	14.34
**Women - 1992-2001**									
Baseline	1431573	28531	17.12	122615	939	13.62	87279	319	11.22
Smoothing	1431573	28531	17.12	122615	939	13.34	87279	319	10.78
Redistribution missings	1436630	28697	17.13	122990	945	13.35	87572	321	10.79
Trend break 1981	1365148	28252	17.22	194472	1390	13.48	87572	321	10.79
Emigration	1365148	28252	17.22	194472	1390	13.48	87572	321	10.79
Alignment national data	1359986	26857	17.24	196923	1327	13.52	90409	306	10.8
**Women - 2002-2011**									
Baseline	1180939	23525	15.08	275667	2112	11.42	229635	737	11.17
Smoothing	1180939	23525	15.09	275667	2112	11.77	229635	737	11.15
Redistribution missings	1191368	24737	15.12	276494	2166	11.79	230109	754	11.17
Trend break 1981	1187461	24581	15.14	280402	2322	11.79	230109	754	11.17
Emigration	1183312	24557	15.14	277691	2315	11.79	226891	749	11.17
Alignment national data	1226109	23251	14.78	290643	2211	11.52	246486	725	10.9
**Women – 2012-2017**									
Baseline	650628	12869	13.27	294771	2709	10.39	237026	710	9.14
Smoothing	650628	12869	13.27	294771	2709	10.40	237026	710	9.06
Redistribution missings	650821	12876	13.27	294877	2710	10.40	237140	710	9.06
Trend break 1981	650821	12876	13.27	294877	2710	10.40	237140	710	9.06
Emigration	618259	12681	13.27	273600	2649	10.40	211886	675	9.06
Alignment national data	622551	12425	13.52	279938	2596	10.58	221868	676	9.23

Source data: ONS-LS & HMD

REFERENCES

CALLS Hub (2022). *Census forms – CALLS-HUB*, The Census & Administrative data LongitudinaL Studies Hub (CALLS Hub). Retrieved December 1, 2022 from http://calls.ac.uk/guides-resources/census-forms/

CeLSIUS (2021). *Events Guide*, Centre for Longitudinal Study Information and User Support (CeLSIUS). Retrieved November 9, 2021 from https://www.ucl.ac.uk/epidemiology-health-care/research/epidemiology-and-public-health/research/health-and-social-surveys-research-group/studies-21

CeLSIUS (2022a). *Data dictionary*, Centre for Longitudinal Study Information and User Support (CeLSIUS). Retrieved December 1, 2022 from https://www.ucl.ac.uk/infostudies/silva-php-resources/researchProjects/celsius/standalone//index.php

CeLSIUS (2022b). *Data dictionary – LS variable metadata HLQP11*, Centre for Longitudinal Study Information and User Support (CeLSIUS). Retrieved December 1, 2022 from https://www.ucl.ac.uk/infostudies/silva-php-resources/researchProjects/celsius/standalone/varDetail.php?tabid=ME11&varid=HLQP11&backurl=unknown

Coleman, L. (2021). A Nurse's View: Changes To Nurse Training And Policy 1967 – 2021. *Nurses.co.uk. https://www.nurses.co.uk/blog/a-nurses-view-changes-to-nurse-training-and-policy-1967---2021/*

de Gelder, R., Menvielle, G., Kovács, K., Martikainen, P., Strand, B., & Mackenbach, J. P. (2017). Long-term trends of inequalities in mortality in 6 European countries. *International Journal of Public Health,* 62(1), 127-141. doi: 10.1007/s00038-016-0922-9

European Commission (2013) Revision of the European Standard Population — Report of Eurostat's task force. *EUROSTAT Methodologies and Working Papers.* Luxembourg: Publications Office of the European Union.

Eurostat (2022) *International Standard Classification of Education (ISCED) – Correspondence between ISCED 2011 and ISCED 1997.* https://ec.europa.eu/eurostat/statistics-explained/index.php?title=International_Standard_Classification_of_Education_(ISCED).

Flanagan, L. & McCartney, G. (2015). How robust is the calculation of health inequality trends by educational attainment in England and Wales using the Longitudinal Study? *Public Health,* 129(6), 621-628. doi: 10.1016/j.puhe.2015.02.027

Gompertz B. (1825). On the Nature of the Function Expressive of the Law of Human Mortality, and on a New Mode of Determining the Value of Life Contingencies. *Philosophical Transitions of the Royal Society of London,* 115 (1825), 513–583.

Greenaway, D. & Haynes, H. (2003). Funding Higher Education in the UK: The Role of Fees and Loans. *The Economic Journal, 113,* F150-F167. doi: 10.1111/1468-0297.00102

Gregoraci, G., Van Lenthe, F. J., Artnik, B., Bopp, M., Deboosere, P., Kovács, K., Looman, C. W. N., Martikainen, P., Menvielle, G., Peters, F., Wojtyniak, B., de Gelder, R., & Mackenbach, J. P. (2017). Contribution of smoking to socioeconomic inequalities in mortality: a study of 14 European countries, 1990-2004. *Tobacco Control,* 26, 260-268. doi: 10.1136/tobaccocontrol-2015-052766

HMD (2021). *Human Mortality Database*. Max Planck Institute for Demographic Research (Germany), University of California, Berkeley (USA), and French Institute for Demographic Studies (France). Retrieved November 19, 2021.

Huisman, M. (2004). Socioeconomic inequalities in health in Europe: Studies of middle-aged and older populations with a special focus on the role of smoking. PhD dissertation, Erasmus MC, University Medical Center Rotterdam.

Ingleby, F. C., Woods, L. M., Atherton, I. M., Baker, M., Elliss-Brookes, L., & Belot, A. (2021). Describing socio-economic variation in life expectancy according to an individual's education, occupation and wage in England and Wales: An analysis of the ONS Longitudinal Study. *Social Science & Medicine - Population Health*, 14, 100815. doi: 10.1016/j.ssmph.2021.100815

Iveson, M., Dibben, D., Deary, I.J. (2020) Childhood socio-economic circumstances, cognitive function and education and later-life economic activity: linking the Scottish Mental Survey 1947 to administrative data. *Longitudinal and Life Course Studies,* 11(1), pp. 55-79.

Janssen, F., & Kunst, A. E. (2004). ICD coding changes and discontinuities in trends in cause-specific mortality in six European countries, 1950-99. *Bulletin of the World Health Organization*, 82(12), 904-913.

Mackenbach, J. P., & Kunst, A. E. (1997). Measuring the magnitude of socio-economic inequalities in health: An overview of available measures illustrated with two examples from Europe. *Social Science & Medicine,* 44(6), 757-771.

Monti, A., Drefahl, S., Mussino, E., & Härkönen, J. (2019). Over-coverage in population registers leads to bias in demographic estimates. *Population Studies,* 76(2), 451-469. doi: 10.1080/00324728.2019.1683219

Moreno-Betancur, M., Latouche, A., Menvielle, G., Kunst, A.E., & Rey, G. (2015). Relative index of inequality and slope index of inequality: a structured regression framework for estimation. *Epidemiology,* 26(4). 518-527. doi: 10.1097/EDE.0000000000000311.

OECD (2022), *Education GPS – United Kingdom.* Accessed April 11, 2022. https://gpseducation.oecd.org/CountryProfile?primaryCountry=GBR

Office for National Statistics (2019). *ONS Longitudinal Study – England and Wales*. Released 11 June 2019, ONS SRS Metadata Catalogue. doi: 10.57906/z9xn-ng05.

Office for National Statistics (2012a). *2011 Census item edit and imputation process.* 2011 Census: Methods and Quality Report December 2012. Titchfield, UK.

Office for National Statistics (2012b). *Response Rates in the 2011 Census*. 2011 Census: Methods and Quality Report December 2012. Titchfield, UK.

Pascariu, M. D., Dańko, M. J., Schöley, J., & Rizzi, S. (2018). Ungroup: An R package for efficient estimation of smooth distributions from coarsely binned data, *Journal of Open Source Software,* 3(29), 937. doi:10.21105/joss.00937

Paterson, L. (2022a) Social class and sex differences in higher-education attainment among adults in Scotland since the 1960s. *Longitudinal and Life Course Studies*, 13(1), 7-48.

Paterson, L. (2022b) Education and high-status occupations in the UK since the middle of the twentieth century, *British Journal of Sociology of Education*, 43(3,) 375-396.

Plewis, I. & Bartley, M. (2014). Intra-generational social mobility and educational qualifications. *Research in Social Stratification and Mobility,* 36, 1-11. doi: 10.1016/j.rssm.2013.10.001

Schneider, S.L. (2008), The application of the ISCED-97 to the UK's educational qualifications. In: S.L. Schneider (Ed.) *The International Standard Classification of Education (ISCED-97). An Evaluation of Content and Criterion Validity for 15 European Countries.* Mannheim: Mannheim Centre for European Social Research (MZES).

Shelton, N., Marshal, C.E. Stuchbury, R., Grundy, E., Dennett, A., Tomlinson, J., Duke-Williams, O., & Xun, W., (2019). Cohort Profile: the Office for National Statistics Longitudinal Study (the LS). *International Journal of Epidemiology,* 48(2), 383–384. doi: 10.1093/ije/dyy243

Shields, L. & Watson, R. (2009). The demise of nursing in the United Kingdom: a warning for medicine. *Journal of the Royal Society of Medicine,* 100(2), 70-74. doi: 10.1258/jrsm.100.2.70

Rizzi, S., Gampe, J., & Eilers, P.H. (2015). Efficient estimation of smooth distributions from coarsely grouped data. *American Journal of Epidemiology,* 182(2), 138-147. doi: 10.1093/aje/kwv020

UNECE (2009). Improving migration statistics by exchange of data between countries. 95th DGINS conference "Migration—Statistical mainstreaming", 1 October 2009, Malta. Retrieved from https://ec.europa.eu/eurostat/documents/1001617/4339944/UNECE.pdf/a94a5000-12af-474e-992d-921219c552a0

UNESCO (1997). *International Standard Classification of Education - ISCED 1997.* Paris: UNESCO Institute for Statistics. Retrieved from http://uis.unesco.org/sites/default/files/documents/international-standard-classification-of-education-1997-en_0.pdf

van Baal, P., Peters, F., Mackenbach, J. P., & Nusselder, W. J. (2016). Forecasting differences in life expectancy by education. *Population Studies*, 70(2), 201-216. doi: 10.1080/00324728.2016.1159718.

Van der Stegen, R.H.M., Koren, L.P.H., Harteloh, P.P.M., Kardaun, W.P.F. & Janssen, F. (2014). A novel time series approach to bridge coding changes with a consistent solution across causes of death. *European Journal of Population*, 30(3), 317-335. doi: 10.1007/s10680-013-9307-4.

APPENDIX I

Screenshots of the questions pertaining to educational level in the different censuses (1971, 1981, 1991, 2001, 2011) in England & Wales

Census 1971

*The remaining questions (13–24) do not apply to children under 15 years of age. If you are an overseas visitor (that is, if you normally reside overseas, are not working or seeking work in England, Scotland or Wales and are visiting this country for less than 1 month) you need not answer these questions. If you are over 70 see special note * below.*

13
Have you obtained any of the following?
G.C.E. **'A' level**
Higher School Certificate (HSC)
Higher grade of Scottish Certificate of Education (SCE)
Higher grade of Scottish Leaving Certificate (SLC)
Ordinary National Certificate (ONC)
Ordinary National Diploma (OND)

1 ☐ GCE 'A' level or HSC
2 ☐ SCE higher or SLC higher
3 ☐ ONC or OND
4 ☐ None of these

14
Have you obtained **any** of the following qualifications since reaching the age of 18?
a H.N.C. or H.N.D.
b Nursing qualifications
c Teaching qualifications
d Degrees, diplomas or other educational qualifications
e Graduate or corporate membership of professional institutions
f Any other professional or vocational qualifications
If so, give full details of **all** such qualifications in the order in which they were obtained, even if not relevant to your present job or if you are not working. If **none,** write 'NONE'.

Qualification	Major Subject or Subjects	Awarding Institution

Answer questions 15-17 in respect of your main employment last week, or of your most recent job if you are retired or out of work.
If you have never had a job or if you are a housewife who did not have a job last week write 'NONE' at 15.

15
What was the **name and business** of your employer (if self-employed, the name and nature of your business)? (see note 15)
Give the trading name if one was used.
a Name of business
b Nature of business

16
a What was your **occupation?** Give full details. (see note 16)
b Describe the **actual work** done in that occupation.
a Occupation
b Description of work

17
Were you an employee, or self-employed employing others (see note 17), or self-employed without employees?
1 ☐ An employee
2 ☐ Self-employed employing others (see note 17)
3 ☐ Self-employed without employees

***Persons over 70**
If you are over 70 and retired you need not answer any questions on this page except 15-17 (which refer to the last job you had).
But if you had a job last week all questions 13-22 should be answered.

Source: Calls-Hub (2022)

Census 1981

16 Degrees, professional and vocational qualifications
Have you obtained any qualifications after the age of 18 such as:
Degrees, Diplomas, HNC, HND,
Nursing qualifications, Teaching qualifications,
Graduate or corporate membership of professional institutions,
Other professional, educational or vocational qualifications?
Exclude qualifications normally obtained at school, such as GCE, CSE and School Certificates.
If box 2 is ticked write in all qualifications even if they are not relevant to your present job or if you are not working.
Please list the qualifications in the order in which they were obtained.
Write for each qualification:
the title
the major subject or subjects
the year obtained and
the awarding institution
If more than four, please enter in the space top right.

1 ☐ NO — none of these
2 ☐ YES — give details

Title ....
Subject(s) ....
Year ....
Institution ....

Title ....
Subject(s) ....
Year ....
Institution ....

Title ....
Subject(s) ....
Year ....
Institution ....

Title ....
Subject(s) ....
Year ....
Institution ....

Source: Calls-Hub (2022)

Census 1991

19 **Degrees, professional and vocational qualifications**

Have you obtained any qualifications after reaching the age of 18 such as:

- degrees, diplomas, HNC, HND,
- nursing qualifications,
- teaching qualifications (see * below),
- graduate or corporate membership of professional institutions,
- other professional, educational or vocational qualifications?

Do not count qualifications normally obtained at school such as GCE, CSE, GCSE, SCE and school certificates.

If box 2 is ticked, write in all qualifications even if they are not relevant to your present job or if you are not working.

Please list the qualifications in the order in which they were obtained.

* If you have **school teaching qualifications,** give the full title of the qualification, such as 'Certificate of Education' and the subject(s) which you are qualified to teach. The subject 'education' should then only be shown if the course had no other subject specialisation.

[illegible] mainly at home ☐ 0

NO - no such qualifications ☐ 1

YES - give details ☐ 2

1 Title	2 Title
Subject(s)	Subject(s)
Year	Year
Institution	Institution
3 Title	4 Title
Subject(s)	Subject(s)
Year	Year
Institution	Institution

Source: Calls-Hub (2022)

Census 2001

15 If you are aged 16 to 74 ► Go to **16**

If you are aged 15 and under, or 75 and over ► Go to **36**

16 Which of these qualifications do you have?

♦ *✓ all the qualifications that apply or, if not specified, the nearest equivalent.*

- ☐ 1+ O levels/CSEs/GCSEs (any grades)
- ☐ 5+ O levels, 5+ CSEs (grade 1), 5+ GCSEs (grades A-C), School Certificate
- ☐ 1+ A levels/AS levels
- ☐ 2+ A levels, 4+ AS levels, Higher School Certificate
- ☐ First Degree (eg BA, BSc)
- ☐ Higher Degree (eg MA, PhD, PGCE, post-graduate certificates/diplomas)
- ☐ NVQ Level 1, Foundation GNVQ
- ☐ NVQ Level 2, Intermediate GNVQ
- ☐ NVQ Level 3, Advanced GNVQ
- ☐ NVQ Levels 4-5, HNC, HND
- ☐ Other Qualifications (eg City and Guilds, RSA/OCR, BTEC/Edexcel)
- ☐ No Qualifications

17 Do you have any of the following professional qualifications?

♦ *✓ all the boxes that apply.*

- ☐ No Professional Qualifications
- ☐ Qualified Teacher Status (for schools)
- ☐ Qualified Medical Doctor
- ☐ Qualified Dentist
- ☐ Qualified Nurse, Midwife, Health Visitor
- ☐ Other Professional Qualifications

Source: Calls-Hub (2022)

Appendix II

Variables used in our educational classification beyond the ONS-LS summary education variables

- **More detailed ONS-LS education variables**

QMQUAL19 (1st qualification 1991) is based on the answers to question 19 of the 1991 census. The question asked informants to include any qualifications obtained after reaching the age of 18 years. Excluded are qualifications normally obtained at school. Informants were also asked to list their qualifications in the order in which they were obtained. QMQUAL19 contains the information on the first qualification listed, and distinguishes the codes 001-478 for different qualifications (See 1991 Census Appendix 10). In addition code -9 indicates “No qualifications obtained since the age of 18”.

QUP0 (Qualifications 2001) has twelve columns. Each column corresponds to one of the twelve boxes in question 16. A '1' indicates that the person ticked the box.
Column 1 - 1+O levels/CSEs/GCSEs (any grades)
Column 2 - 5+O levels,5+CSEs (grade 1), 5+GCSEs (grades A-C),School Certificate
Column 3 - 1+A levels/AS levels
Column 4 - 2+A levels,4+AS levels,Higher School Certificate
Column 5 - First Degree (eg BA,BSc)
Column 6 - Higher Degree (eg MA,PhD,PGCE, post-graduate certificates/diplomas)
Column 7 - NVQ Level 1,Foundation GNVQ
Column 8 - NVQ Level 2,Intermediate GNVQ
Column 9 - NVQ Level 3,Advanced GNVQ
Column 10 - NVQ Level 4-5,HNC,HND
Column 11 - Other Qualifications (eg City and Guilds,RSA/OCR,BTEC/Edexcel)
Column 12 - No qualifications.

PQUPO (Professional qualifications 2001) has six columns. Each column corresponds to one of the six boxes in question 17 of the 2001 census. A '1' in columns one to five indicates that the person ticked the box.
Column 1 - No Professional Qualifications.
Column 2 - Qualified Teacher Status (for schools)
Column 3 - Qualified Medical Doctor
Column 4 - Qualified Dentist
Column 5 - Qualified Nurse,Midwife,Health Visitor.
Column 6 – Other Professional Qualifications: 0 Has no other Professional Qualifications 1 Has other Professional Qualifications 2 Not applicable.

QUP11 (Qualifications 2011) is a 13-digit variable which corresponds with the 13 options outlined in 2011 census question P25. “which of these qualifications do you have?”. Respondents could tick the boxes that applied to them. A 1 in QUP11 indicates that the respondent had ticked the box.
Column 1 - 1-4 O levels/CSEs/GCSEs (any grades), Entry Level, Foundation Diploma
Column 2 - NVQ Level 1, Foundation GNVQ, Basic Skills
Column 3 – 5+ O levels (passes)/CSEs (grade 1)/GCSEs (grades A-C), School Certificate, 1 A level/2-3 AS levels/VCEs, Higher Diploma
Column 4 - NVQ Level 2, Intermediate GNVQ, Basic Skills
Column 5 – Apprenticeship
Column 6 - 2+ A levels/VCEs, 4+ AS levels, Higher School Certificate, Progression/Advanced Diploma
Column 7 - NVQ Level 3, Advanced GNVQ, City and Guilds, Advanced Craft, ONC, OND, BTEC National, RSA Advanced Diploma

Column 8 - Degree (for example BA, BSc), Higher degree (for example MA, PhD, PGCE)
Column 9 - NVQ Level 4-5, HNC, HND, RSA Higher Diploma, BTEC Higher Level
Column 10 - Professional qualifications (for example teaching, nursing, accountancy)
Column 11 - Other vocational/work-related qualifications
Column 12 - Foreign qualifications
Column 13 - No qualifications

- **Additional educational variables that we defined for 2001 based on PQUP0 and QUP0 to come to our final education variable:**

Qualprofdegree = Professional qualification with or without a degree =>
0: Professional qualification without a degree
1: Professional qualification with a degree
Based on highestprof2001 and highestacad2001

gen qualprofdegree =.
replace qualprofdegree = 0 if highestprof2001==1
replace qualprofdegree = 1 if highestprof2001==1 & highestacad2001==4

Highestprof2001 = Highest professional qualification in 2001 =>
0: None
1: Teacher up to nurse
2: Other professional qualifications

generate highestprof2001=.
replace highestprof2001=0 if pqup0_1_bin==1
replace highestprof2001=2 if pqup0_6_bin==1
replace highestprof2001=1 if pqup0_2upto5_bin==1

where *pqup0_[x]_bin* is the pqup0 variable with its x columns translated into a binary variable related to the x-th column.

Highestacad2001 = Highest academic qualification in 2001 =>
0: None
1: nvq1/ olevel any
2: nvq2/ olevelAC/ alevel1
3: nvq3/ alevel2
4: nvq4-5/degree+
5: other

generate highestacad2001=.
replace highestacad2001=0 if qup0_12_bin==1
replace highestacad2001=5 if qup0_11_bin==1
replace highestacad2001=1 if qup0_1_bin==1 | qup0_7_bin==1
replace highestacad2001=2 if qup0_2_bin==1 | qup0_3_bin==1 | qup0_8_bin==1
replace highestacad2001=3 if qup0_4_bin==1 | qup0_9_bin==1
replace highestacad2001=4 if qup0_5_bin==1 | qup0_6_bin==1 | qup0_10_bin==1

where *qup0_[x]_bin* is the qup0 variable with its x columns translated into a binary variable related to the x-th column.

Otherprofother = Other professional qualifications with or without “other academic qualification”=>
0 = highest qualification is other professional qualification, but no other academic qualification
1 = highest qualification is other professional qualification AND other academic qualification

generate otherprofother = .
replace otherprofother = 0 if highestprof2001==2
replace otherprofother = 1 if highestprof2001==2 & highestacad2001==5

APPENDIX III

A. **Trends in the share of personyears and deaths by educational attainment group (%), for individuals aged 30 and older, by sex, Italy (1972-2019).** The vertical dashed lines indicate the start of the different 10-year follow-up periods.

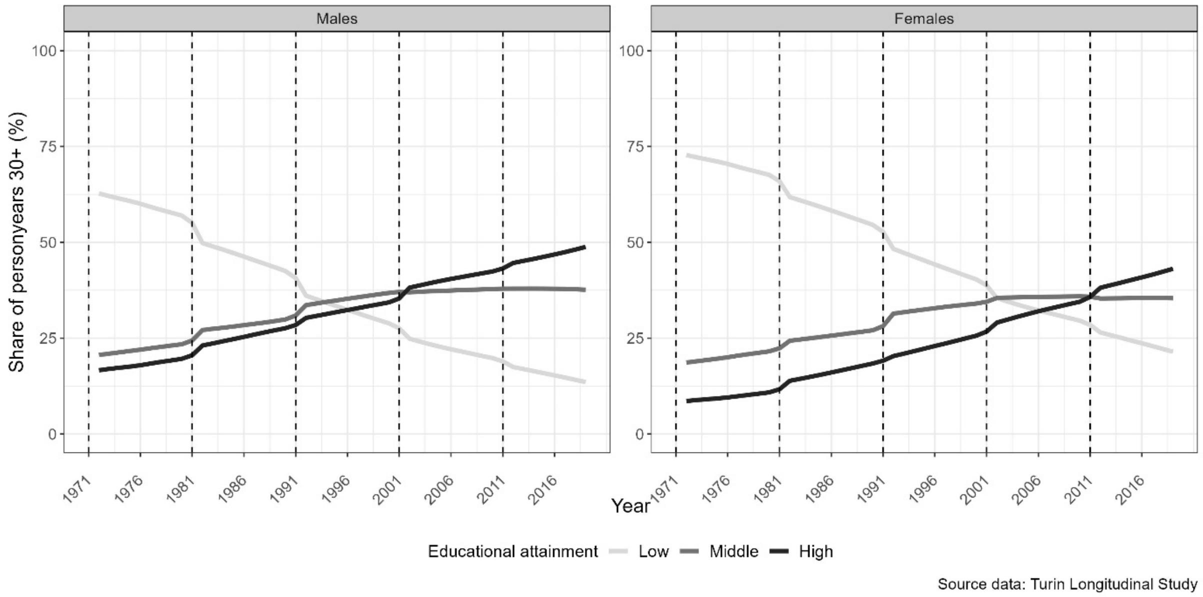

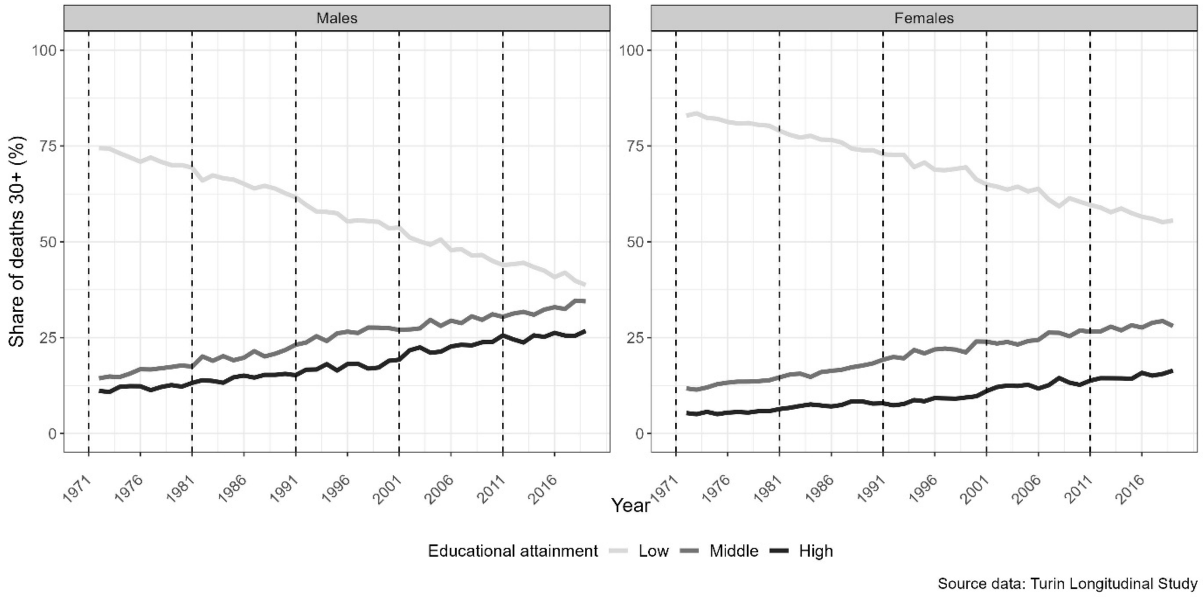

B. **Trends in the share of personyears and deaths by educational attainment group (%), for individuals aged 30 and older, by sex, Finland (1971-2017).** The vertical dashed lines indicate the start of the different 5-year follow-up periods.

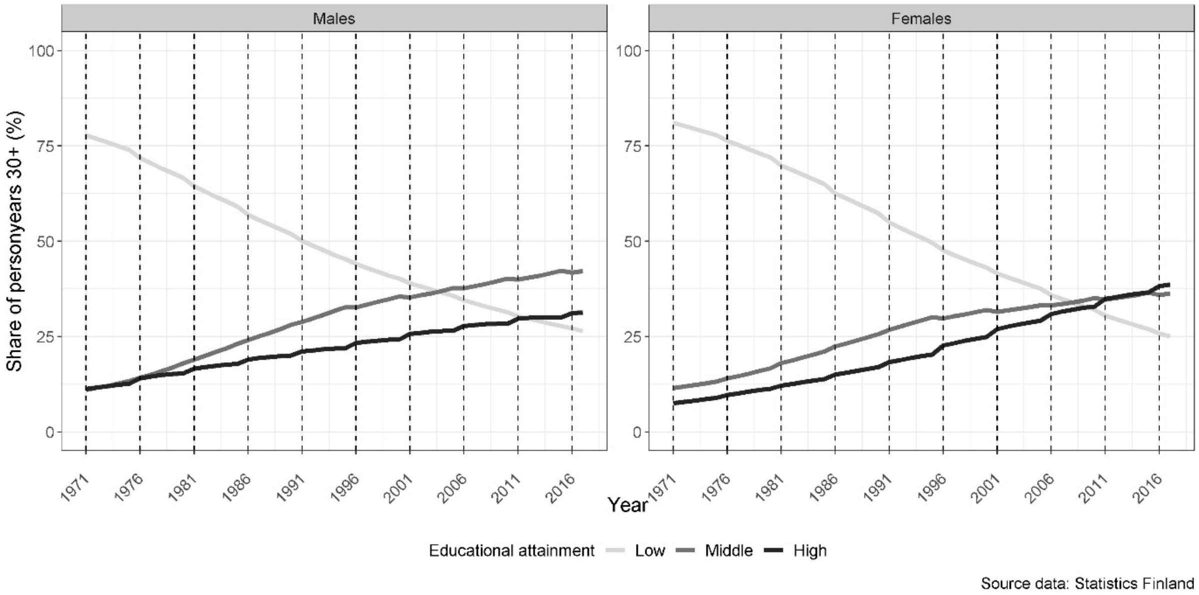

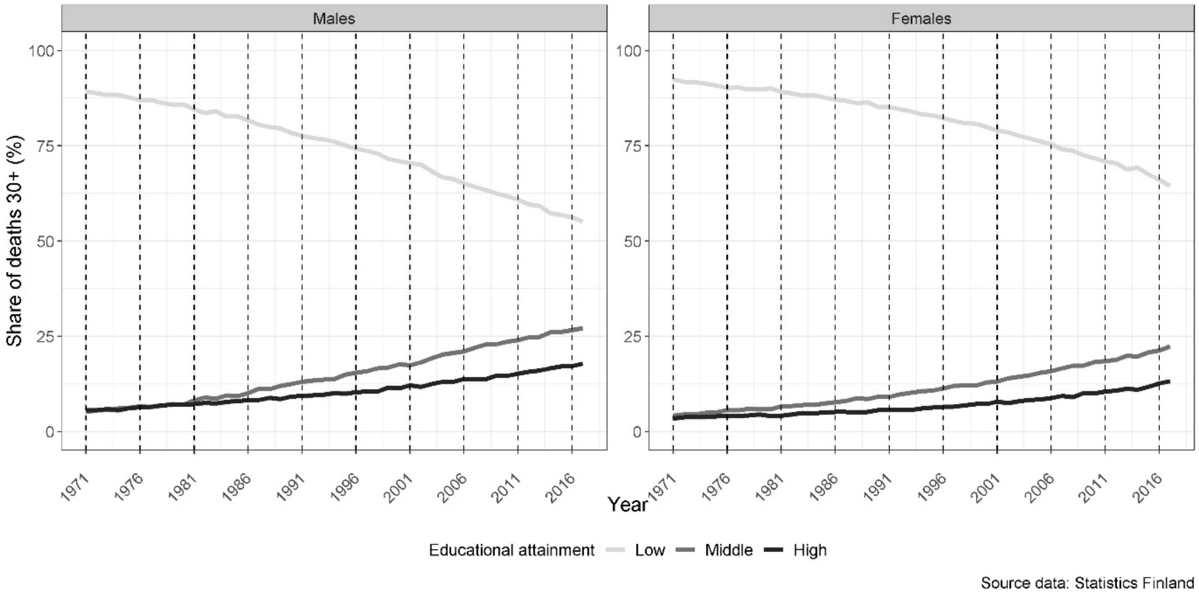

Supplement: Supplementary file 1 — Additional file 1. Supplementary data and methods. [file 12963_2024_324_MOESM1_ESM.pdf]
